# Grid-cell modules remain coordinated when neural activity is dissociated from external sensory cues

**DOI:** 10.1016/j.neuron.2022.03.011

**Published:** 2022-06-01

**Authors:** Torgeir Waaga, Haggai Agmon, Valentin A. Normand, Anne Nagelhus, Richard J. Gardner, May-Britt Moser, Edvard I. Moser, Yoram Burak

**Affiliations:** 1 Kavli Institute for Systems Neuroscience and Centre for Neural Computation, Norwegian University of Science and Technology, Trondheim, Norway; 2 Edmond and Lily Safra Center for Brain Sciences, The Hebrew University of Jerusalem, Jerusalem, Israel; 3 Racah Institute of Physics, The Hebrew University of Jerusalem, Jerusalem, Israel

**Keywords:** neural coding, neural decoding, grid cells, entorhinal cortex, spatial memory, spatial coding, population coding, neural circuits

## Abstract

The representation of an animal’s position in the medial entorhinal cortex (MEC) is distributed across several modules of grid cells, each characterized by a distinct spatial scale. The population activity within each module is tightly coordinated and preserved across environments and behavioral states. Little is known, however, about the coordination of activity patterns across modules. We analyzed the joint activity patterns of hundreds of grid cells simultaneously recorded in animals that were foraging either in the light, when sensory cues could stabilize the representation, or in darkness, when such stabilization was disrupted. We found that the states of different modules are tightly coordinated, even in darkness, when the internal representation of position within the MEC deviates substantially from the true position of the animal. These findings suggest that internal brain mechanisms dynamically coordinate the representation of position in different modules, ensuring that they jointly encode a coherent and smooth trajectory.

## Highlights

• Hundreds of grid cells were recorded simultaneously from multiple grid modules
• Coordination between grid modules was assessed in rats that foraged in darkness
• Coordination persists despite relative drift of the represented versus true position
• This suggests that internal network mechanisms maintain inter-module coordination

## Teaser

The position of an animal in its environment is represented in the entorhinal cortex by distinct modules of grid cells. Waaga, Agmon et al. show that in darkness, the internal representation of position drifts relative to the true position of the animal; yet, it remains tightly coordinated across the distinct modules.

## Introduction

Recently, techniques that enable simultaneous recording of activity in dozens to hundreds of neurons (Ghosh et al., 2011; Jun et al., 2017; Steinmetz et al., 2021; Zong et al., 2017) have enabled a shift from the measurement of single-cell activity in relationship to external correlates to the investigation of the joint population activity patterns in large neural ensembles. This change of perspective has led to various attempts to characterize neural activity patterns as residing within restricted, low-dimensional spaces using linear (Gallego et al., 2018; Mazor and Laurent, 2005; Stringer et al., 2019) or non-linear (Chaudhuri et al., 2019; Gardner et al., 2022; Rubin et al., 2019; Rybakken et al., 2019) dimensionality reduction techniques. One of the most striking outcomes of these attempts has emerged in neural circuits involved in the representation of an animal’s position relative to the environment. In several such circuits in flies and mammals, neural activity patterns have been shown to robustly reside in low-dimensional non-linear manifolds, even when the neural activity is dissociated from external inputs to the network (Chaudhuri et al., 2019; Gardner et al., 2022; Kim et al., 2017; Rybakken et al., 2019; Seelig and Jayaraman, 2015). This finding opens up the possibility to decode the low-dimensional variable that is represented within these circuits and to examine how the brain utilizes such representations across multiple sub-circuits to implement computational functions.

Here, we examine the dynamics of grid cells in the medial entorhinal cortex (MEC). Grid cells exhibit multiple firing fields as a function of an animal’s spatial location. The fields are arranged on a hexagonal lattice in open-field environments (Hafting et al., 2005). Within each individual animal, grid cells are allocated to discrete modules, each defined by a common grid spacing and angular orientation (Barry et al., 2007; Stensola et al., 2012). Jointly, the activity of grid cells across multiple modules implements a highly efficient population code for position (Burak, 2014; Fiete et al., 2008; Mathis et al., 2012; Sreenivasan and Fiete, 2011; Welinder et al., 2008).

The spatial tuning curves of individual grid cells indicate that grid-cell population activity within each module is confined to lie on a low-dimensional manifold with toroidal topology (Gardner et al., 2022). Accumulating evidence has suggested that this confinement is achieved through network mechanisms within the MEC, under diverse behavioral conditions and independently of inputs from other brain regions. Early evidence came from observing the correlation structure of activity in pairs of cells: phase relationships between grid cells within a module are tightly preserved over time and across environments (Fyhn et al., 2007; Yoon et al., 2013). The phase relationships are maintained also during sleep (Gardner et al., 2019; Trettel et al., 2019) and under hippocampal inactivation, despite the absence of a grid-like spatial response pattern (Almog et al., 2019). Very recently, simultaneous recordings of spiking activity in dozens of cells provided direct evidence that neural activity patterns are closely confined to two-dimensional manifolds with toroidal topology, which are tightly preserved across environments and in sleep (Gardner et al., 2022). Thus, grid cells within a module encode together a two-dimensional quantity, which, in some conditions, could be dissociated from the true position of the animal.

All of the above findings are in agreement with predictions made by continuous attractor network (CAN) theory (Burak and Fiete, 2009; Fuhs and Touretzky, 2006; Guanella et al., 2007; McNaughton et al., 2006). According to this theory, grid cells within each module are recurrently connected and thus form a sub-network within the MEC. The recurrent synaptic connectivity within each module constrains the joint activity of cells to a restricted, but continuous, repertoire of possible coactivation patterns that is stable across behavioral states and conditions, even in the absence of sensory inputs.

Single modules alone, however, cannot represent a unique position of an animal within a typical environment. It is necessary to consider the coordination of activity across modules to assess how grid cells encode the brain’s internal representation of position. The question of coordination becomes especially important under conditions in which sensory cues are poor or absent (Burak, 2014). Since population activity of an individual module lies on a two-dimensional manifold, the joint activity of *M* modules spans, at least in principle, a 2*M* dimensional space. However, during continuous motion in a given environment, and in the presence of salient sensory cues, the state of each module is faithfully mapped to the location of the animal in two-dimensional space. Hence, under continuous motion of the animal, the joint population activity patterns of multiple modules span a highly restricted two-dimensional subspace of the full *2M* dimensional space. This raises the question as to whether the states of different modules are updated in a similarly coordinated manner when the states of individual modules are dissociated from the true position of the animal, e.g., in the absence of salient sensory cues.

The coordination of activity across grid-cell modules is highly consequential from the perspectives of neural coding and dynamics. The modular structure of the grid-cell code for position confers it with large representational capacity (Burak, 2014; Fiete et al., 2008; Mosheiff and Burak, 2019; Sreenivasan and Fiete, 2011; Welinder et al., 2008). However, within a given environment, the modularity of the grid-cell code poses a significant challenge for the neural circuitry that maintains the representation and updates it based on self-motion. Under conditions in which sensory inputs are absent or poor, the representation of position in individual grid-cell modules might drift relative to the actual position of the animal. If these drifts are not identical in different modules, they would rapidly lead to combinations of spatial phases that do not represent any position in the vicinity of the animal, resulting in abrupt shifts in the represented position. Thus, independent drifts lead to catastrophic errors when activities are read out from multiple grid-cell modules and would therefore be highly detrimental for the coding of position by grid-cell activity. The difficulty arising from occurrence of such catastrophic readout errors has been identified in early works on grid-cell coding (Fiete et al., 2008). Since then, two solutions have been proposed. In one solution (Agmon and Burak, 2020; Sreenivasan and Fiete, 2011; Welinder et al., 2008), the hippocampal network reads out the position represented by grid cells, and feedback projections from hippocampus to the MEC correct small, incompatible drifts accrued in each of the modules. A second solution (Kang and Balasubramanian, 2019; Mosheiff and Burak, 2019) involves synaptic connectivity between modules.

Empirically, however, very little is known about the relationship between population activity patterns of grid cells across distinct modules. Previous research has focused on coactivation patterns within modules for two reasons: first, simultaneously recorded cells using tetrodes often belonged to the same module. Second, coactivation patterns of inter- and intra-module grid-cell pairs are fundamentally different. Grid cells within a module maintain strict relationships in their activities that can be probed by analyzing the joint activity in pairs of simultaneously recorded cells. On the other hand, the activity of two cells that belong to different modules might be correlated or anti-correlated depending on the animal’s position, even within a fairly small environment. Due to this lack of an expected rigid correlation (or anti-correlation), it is difficult to characterize inter-module coordination based on pair recording analysis. To identify higher-order dependencies across modules, it is necessary to decode activity from multiple cells within each module—requiring larger numbers of simultaneously recorded cells from multiple modules, which have only recently become available.

Here, using Neuropixels silicon probes (Jun et al., 2017; Steinmetz et al., 2021), we recorded the simultaneous activity of grid cells from multiple modules with dozens of units in each module. Rats were deprived of visual cues to test whether the internal representations of position in distinct modules remain coordinated even when dissociated from the animal’s true position. By decoding the simultaneous grid-cell activity, we demonstrated that grid-cell modules retain, to a high extent, coordination even when the mapping between grid-cell activity and position deteriorates. These results indicate that network mechanisms within the brain coordinate the activity of different modules, independently of external sensory inputs.

## Results

**Figure 1:**
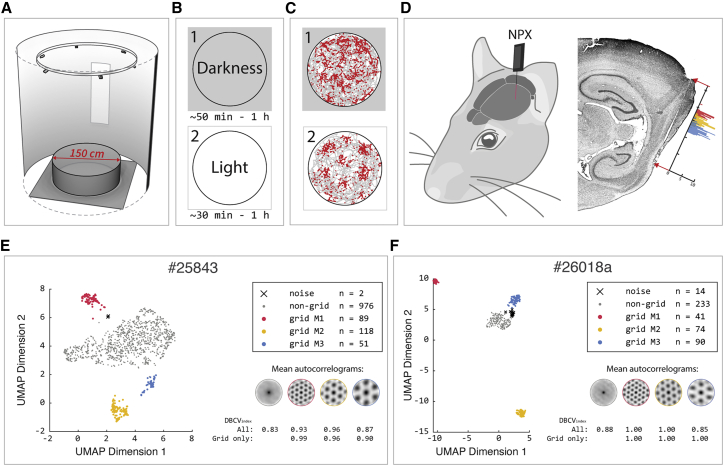
Experimental setup and module classification (A) Recording arena: electrophysiological recordings of the spiking activity in the MEC were collected while the rat ran freely in a 150 cm diameter cylindrical arena surrounded by a floor-to-ceiling blind and a single, fixed, and tactile inaccessible cue card. (B) Protocol: first the animals ran in darkness, then in the same arena with the lights on. (C) The spikes of a representative grid cell from the most dorsal module (red distribution in D) are superimposed in red on the traveled path of the rat in gray, in the dark (1) and light (2) trials. (D) Left: illustration of implantation site for Neuropixels probe. Right: sagittal section of the rat brain (#26018), showing the probe shank through the superficial layers of MEC. The histogram shows the grid-cell count across dorso-ventral recording depths from three modules. The distance between two adjacent ticks along the probe shank axis corresponds to 1 mm. (E and F) Module classification for the two recording sessions with the largest number of simultaneously recorded grid cells. Left: for each recording, scatterplots show the two-dimensional UMAP (McInnes et al., 2018) projection of all recorded units’ autocorrelograms. Each point is color coded by its DBSCAN cluster assignment (Ester et al., 1996). Right: mean autocorrelograms for each cluster and validity (DBCV) index (STAR Methods), including or excluding the non-grid cluster.

We recorded spiking activity from rats foraging in a circular arena with a diameter of 150 cm under light and complete dark conditions (Figure 1A). The arena was cue-less except for a single vertical cue card at a fixed location along its circumference, which was visible in light and completely invisible in darkness, and tactilely inaccessible. The circular arena was rotationally symmetric, thus minimizing the information about absolute position coming from encounters with the walls (Hardcastle et al., 2015; Keinath et al., 2018). The arena was surrounded by a floor-to-ceiling blockout blind to eliminate access to distal visual cues, and the experimental protocol was designed to minimize other positional cues (STAR Methods).

Data were collected from four animals and an overall of five recording sessions, each consisting of a 50–60 min recording in the dark immediately followed by a 30–60 min recording in the light (Figures 1B and 1C). Neuropixels probes were implanted in MEC (Figures 1D and S1). Out of 3,310 recorded cells with >500 spikes in the five light trials, 842 grid cells were identified and classified into modules as described in STAR Methods and in Gardner et al. (2022). Briefly, a non-linear dimensionally reduction technique (McInnes et al., 2018) was applied to feature vectors derived from the spatial autocorrelation of each cell-rate map, followed by clustering (Ester et al., 1996). The procedure ensures modular separation as grid cells with similar auto-correlograms, and thus similar spacing and orientation, are separated into distinct clusters, whereas cells lacking a spatially periodic tuning feature are separated from the grid-cell clusters. In addition to overcoming problems from traditional classification methods caused by skewed grid patterns, false positives would only occur in the unlikely event that the auto-correlogram of a non-grid cell randomly has a pattern with the same spacing and orientation as the real grid cells. Using this procedure, a clear and unambiguous clustering of grid cells into modules was observed in all the sessions (Figures 1E, 1F, and S2). In four out of five recording sessions, many simultaneously recorded grid cells (ranging from 31 to 118 in individual modules) were obtained from three distinct modules, and in one session, such data were obtained from two distinct modules (Table 1).

**Table 1:** Numbers of simultaneously recorded grid cells (allocated to modules) for each recording session

Recording session	Module 1	Module 2	Module 3
#25843	89	118	51
#26018a	41	74	90
#26018b	40	68	78
#26820	31	44	35
#26718	47	36	–

**Figure 2:**
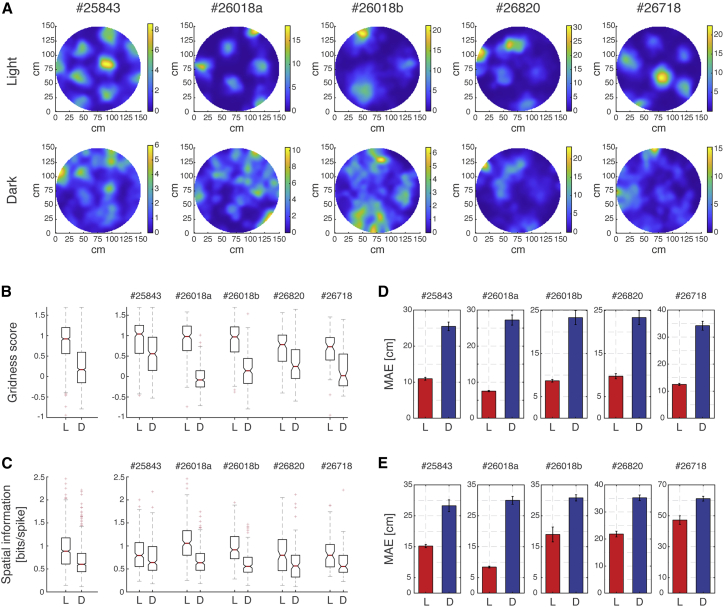
Grid-cell spatial responses deteriorate in darkness (A) Example of rate maps from each session for light and darkness conditions. Three more examples from each recording session are shown in Figure S3A. (B) Gridness scores in light and darkness for all cells across all recording sessions (left) and for all cells from single recording sessions (right). Red lines indicate median, and the lower and upper box limits indicate 1^st^ and 3^rd^ quartiles, respectively. Whisker lengths indicate 1.5 times the interquartile range. Red crosses show outliers that lie more than 1.5 times outside the interquartile range. Notches indicate 95% confidence interval of the median. (C) Spatial information in light and darkness for all cells across all recording sessions (left) and for all cells from single recording sessions (right). Box plots are plotted as in (B). (D) Mean absolute error (MAE) of the Markov decoder (STAR Methods) in light (red) and darkness (blue) for each recording session. Error bars are ±SEM. (E) Same as (D) but for the kernel decoder (STAR Methods).

Several measures indicated that the association between grid-cell activity and the position of the animal deteriorated in the sensory-deprived condition. The characteristic periodicity of grid cells was significantly disrupted in the dark trials (Figures 2A and S3A). Gridness score and information content were considerably reduced compared with the baseline light trials (Figures 2B and 2C). In addition, we decoded position from the population activity patterns and compared the magnitude of decoding errors in the light and dark trials. We have done so using two types of decoders that are used later in the manuscript and are described in STAR Methods. The mean absolute error (MAE) of the decoded position relative to the animal’s true position was substantially larger in dark trials than in baseline light trials (Figures 2D and 2E). This was consistent when the decoding of simultaneous spike trains from dark trials was performed using either light- or dark-generated rate maps (Figures S3B and S3C) and across the two decoders. For this reason, and since dark-generated rate maps are degraded, the rate maps used for decoding (both in light and dark trials) from here onward were extracted from the full extent of the light trials.

### Pairwise correlations

**Figure 3:**
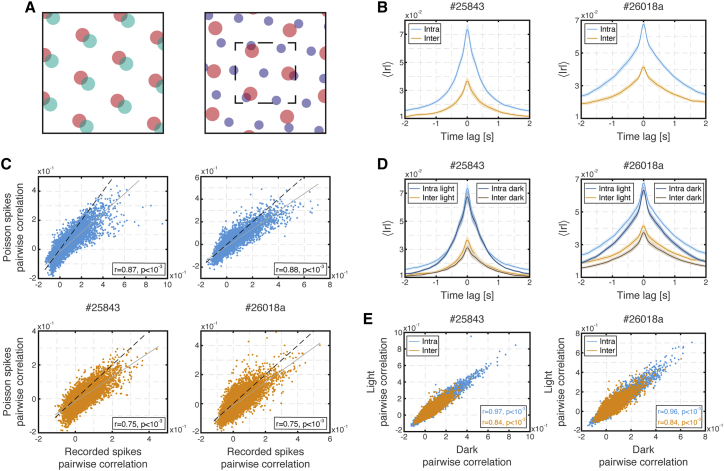
Spike-rate correlations of intra- and inter-module pairs (A) Schematic illustration showing spatial tuning curves of two intra-module (left, red, and turquoise) and two inter-module (right, blue, and red) grid cells. Firing fields of intra-module pairs (left) either overlap throughout the whole environment (as shown in the figure) causing temporally correlated firing or are disjoint throughout the whole environment causing anti-correlated firing. In contrast, in inter-module pairs (right), the degree of correlation (or anti-correlation) between firing fields varies in different parts of the environment: firing fields overlap inside the small dashed square and are disjoint elsewhere. Consequently, absolute spike-rate correlations tend to be weaker in inter-module pairs than in intra-module pairs. This effect is more pronounced in large environments compared with small environments (Figures S4A and S4B). (B) Absolute cross-correlation (Pearson coefficient) of inter- and intra-module light spiking activities, averaged over cell pairs, in the two recording sessions with the largest number of simultaneously recorded neurons (left and right panels). Shaded error bars are ±SEM. (C) Pairwise correlations of all possible intra- (top) and inter- (bottom) module pairs from recorded light spiking activity versus Poisson generated spikes using measured rate maps and the corresponding recorded light trajectory. Correlation coefficient and p value are specified in the inset. Fit shown in solid gray and the identity line is shown for reference (dashed black line). (D) Same as (B) but with superimposed inter- and intra-module spiking activities from darkness. (E) Pairwise correlations of all possible inter- and intra-module pairs from recorded light spiking activity versus recorded dark spiking activity.

As a first step to address the question of inter-module coordination, we considered pairwise correlations of the spiking activity, smoothed with a 50 ms Gaussian kernel (spike-rate correlations, STAR Methods), similar to previous works that were based on tetrode recordings (Almog et al., 2019; Chen et al., 2016; Fyhn et al., 2007; Gardner et al., 2019; Pérez-Escobar et al., 2016; Trettel et al., 2019; Yoon et al., 2013). Differences in the correlation structure of inter- versus intra-module pairs were expected under light conditions for the following reason: the activity of intra-module grid-cell pairs is either correlated or anti-correlated irrespective of the position of the animal, whereas the activity of inter-module grid-cell pairs can be correlated in some parts of the environment and uncorrelated in others (Figure 3A). Consequently, weaker absolute spike-rate correlations were expected on average in inter-module pairs compared with intra-module pairs. Indeed, under light conditions, intra-module pairs showed higher absolute spike-rate correlations compared with inter-module pairs (Figure 3B). The observed zero-lag correlations could be explained quite well by the cells’ rate maps and the animal’s trajectory, even in inter-module pairs (Figure 3C). Thus, the correlations observed in the spiking activity of inter-module pairs were weak but still driven, to a large extent, by their spatial selectivity.

Having established that spike-rate correlations are related to spatial selectivity both in intra-and inter-module pairs, we next compared pairwise spike-rate correlations in dark and light trials. We reasoned that if, in darkness, grid cells cease to consistently encode a unique spatial location, their spike-rate correlations would diminish. The absolute magnitude of the spike-rate correlations in *intra*-module pairs was similar, on average, to that observed in the light (Figure 3D), even when, in the dark, the spatial stability of single-grid cell representations was disrupted. This is in accordance with recordings performed in mice in dark environments (Chen et al., 2016; Pérez-Escobar et al., 2016), with results found in sleep (Gardner et al., 2019), and as expected based on CAN models. However, the average absolute magnitude of spike-rate correlations in *inter*-module pairs was similar in light and dark conditions as well. Furthermore, zero-lag correlations were preserved between light and dark conditions at the level of individual cell pairs both for inter- and intra-module pairs (Figure 3E).

The preservation of inter-module spike-rate correlations in the dark supports the hypothesis that modules maintain coordination even in the absence of sensory cues. However, it is difficult to interpret this result quantitatively, since inter-module spike-rate correlations are already low even in the light and may be influenced also from sources other than the correlation between their spatial receptive fields, such as co-fluctuation of firing rates in the entire population (Okun et al., 2015; Figures S4C and S4D). Analysis of our simultaneous recordings from dozens of grid cells per module (Table 1) could potentially overcome these limitations by revealing higher-order dependencies in the activity of cells from different modules that are not strongly evident in spike-rate correlations within pairs of cells. Therefore, we next analyzed the recorded simultaneous population activities using two complementary approaches.

### Likelihood of the simultaneous population spike trains

In the absence of sensory cues and under the hypothesis of inter-module coordination, a unique position should be coherently represented by the joint activity of grid cells in different modules, even when spatial firing patterns of individual grid cells seem disrupted. When the joint representation is read out under these conditions, it can, however, deviate substantially from the animal’s true position compared with baseline light trials. We therefore sought to identify a measure for the coherence of the joint simultaneous spike trains, which is *independent* of the animal’s true position.

In the first analysis approach, we derived the likelihood of the simultaneously recorded spike trains, summed over all possible trajectories (thus, independently of the actual trajectory) under simple assumptions that are outlined below. This likelihood is written as

(Equation 1) $$p\left(S_{t}\right)=\sum_{X_{t}}p\left(X_{t}\right)\cdot p\left(S_{t}|X_{t}\right)$$

where $S_{t}$ represents the simultaneous spike trains emitted by all the neurons in the population from the beginning of the experiment up to time t, and $X_{t}$ represents a particular trajectory of the animal. The likelihood of the spike trains conditioned on the trajectory, $p\left(S_{t}|X_{t}\right)$, is evaluated under the assumption of Poisson firing, with a rate that is determined by $X_{t}$ and by the tuning curves of all the neurons in the population. Finally, we assumed that the trajectories are continuous (following random walk statistics for simplicity), enforced through the prior $p\left(X_{t}\right)$ (see also STAR Methods).

On the right-hand side of Equation 1, a probability is assigned to each particular realizable trajectory irrespective of the spike trains, followed by a multiplication with the probability of the simultaneously recorded spike trains conditioned on this particular trajectory. This probability is subsequently averaged over all possible trajectories, weighted by the prior. The outcome $p\left(S_{t}\right)$ can be interpreted as a measure that describes the likelihood that the simultaneously recorded spike trains represent some continuous (yet unknown) trajectory, drawn from the prior distribution. Importantly, in a practical implementation there is no need to explicitly calculate the specific probabilities for each of the possible trajectories, which would be unfeasible. Instead, we derived a simpler, exact analytical expression for the average log likelihood per time bin, denoted by L (STAR Methods; Equation 3). The evaluation of L using this expression relies on the Markov property of the spiking model and the prior $p\left(X_{t}\right)$ and involves decoding of the spiking activity using a *Markov decoder* (STAR Methods; Methods S1).

**Figure 4:**
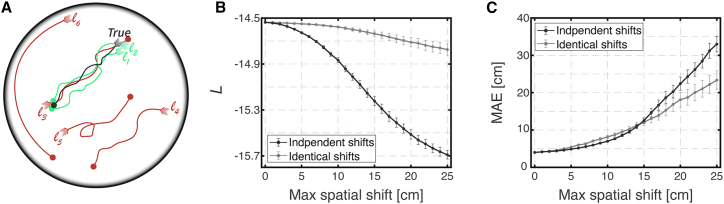
Likelihood of simultaneous spike trains can serve as a measure of coordination across modules (A) Schematic illustration demonstrating the likelihood approach with a few illustrated trajectories, each starting from an arrowhead and ending in a point. The true trajectory of the animal is the black trace. The likelihood of the simultaneous spike trains is evaluated independently for each specific trajectory and is averaged across all possible trajectories. A few possible representative trajectories are illustrated: trajectories $l_{1}$ and $l_{2}$ (turquoise) are two trajectories which are close to the true trajectory and thus have a high likelihood based on the spiking activity. Trajectory $l_{3}$ is very similar to trajectories $l_{1}$ and $l_{2}$ but has low likelihood (red) since it goes in the opposite direction, thus having reversed temporal structure. Trajectory $l_{4}$ is an identical copy of trajectory $l_{2}$ but at a different part of the arena, thus also having low likelihood (red). Trajectories $l_{5}$ and $l_{6}$ are two other trajectories with low likelihood (red). (B) Likelihood of simulated Poisson spikes using measured rate maps and the recorded light trajectory from recording session #26018b, evaluated versus varying magnitudes of spatial shifts. When shifts are applied independently in each module (black trace), the likelihood decreases significantly, but it decreases only slightly (due to boundary conditions) when these shifts are identical (gray trace). Error bars are ±SEM. (C) The corresponding mean absolute error (MAE) of the decoder. The MAE increases significantly both for independent and for identical spatial shifts as their magnitude increases. Error bars are ±SEM.

In the expression for the likelihood (Equation 1), it is assumed that all the neurons fire in response to the same trajectory $X_{t}$, regardless of the identity of the module to which they belong. If this assumption is correct, then this trajectory, as well as nearby trajectories, will make a large contribution to the likelihood (Figure 4A). However, if different modules accrue drifts independently in the dark and thus represent internally different trajectories, there will be no single trajectory in the sum within Equation 1 that makes a large contribution to the likelihood, and we expect the sum to be significantly smaller. Thus, the likelihood introduced above (Equation 1) can serve as a measure of coherence.

To validate that the likelihood can be used to distinguish between the scenarios of coordinated and uncoordinated drifts across modules, we first analyzed simulated spike trains. All neurons in the simulated data fired according to the same recorded trajectory and based on measured tuning curves, which were taken from one of our datasets. Therefore, the different modules were precisely coordinated in the simulation. To mimic the consequences of uncoordinated drifts across modules in a way that can be applied also to recorded datasets, we introduced artificial spatial shifts during the decoding process in the neuron’s rate maps (STAR Methods) that, for simplicity, were constant throughout each simulation. Such shifts were identical within each module but drawn randomly and independently in different modules (and were thus uncoordinated across modules).

We observed that applying independent spatial shifts reduced the likelihood (Figure 4B, black trace). As expected, such shifts also increased the MAE of the decoder with respect to the true position (Figure 4C, black trace). In contrast, the likelihood was nearly unaffected when the artificial spatial shifts were identical across all modules, even though the MAE with respect to the true position increased significantly (Figures 4B and 4C, gray traces). The small reduction in the likelihood seen in Figure 4B (gray trace) is due to boundary effects and vanishes when such effects are eliminated (Figure S4E; see also Figure S6B below). The application of spatial shifts to the neuron’s rate maps during decoding is analogous to application of shifts in the encoded position (Figure S4F; Note that the latter can be applied only to simulated data). The results shown in Figure 4 confirmed that the average log likelihood could be used as a measure of coherence of the simultaneous spike trains.

We next applied the likelihood-based approach to recorded datasets from the light and dark conditions (5 recordings sessions from 4 animals; Table 1) to assess whether modules remain coordinated in the dark. If, in the dark, the phases of individual modules accrued independent drifts relative to the animal’s true position, a reduction in the likelihood would be expected relative to the light. To faithfully compare likelihoods between light and dark conditions, it was necessary to take into account modifications in the mean firing rates of individual neurons across the two conditions: specifically, mean firing rates were more likely to reduce than increase in darkness, resembling previous results from mice (Pérez-Escobar et al., 2016). Thus, we evaluated *rate-adjusted* likelihoods of dark and light simultaneous spike trains, obtained by down-sampling spikes to match the mean firing rates between the two trials (STAR Methods; Methods S1), henceforth referred for convenience as likelihood.

**Figure 5:**
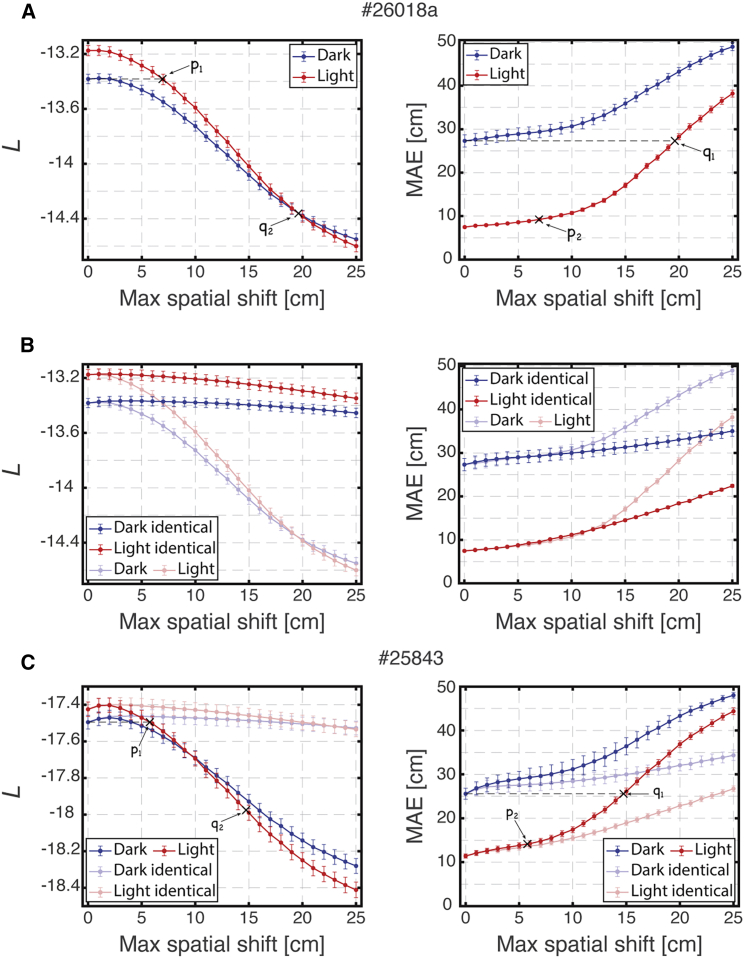
Analysis of likelihood of recorded simultaneous spike trains indicates coordination across modules (A) Likelihood of simultaneously recorded spike trains (left) and corresponding mean absolute error (MAE, right) from dark and light trials, shown for varying magnitudes of independent module-wise spatial shifts applied on a single recording session (#26018a). Applying a maximal spatial shift of 6.9 cm in the light recording achieves the same likelihood as that of the dark recording with zero spatial shift (point p_1_, left panel) but generates only a slight increase of less than 2 cm in the corresponding MAE of the light recording relative to its zero spatial shift value (point p_2_, right panel). Conversely, applying a maximal spatial shift of 19.6 cm in the light recording achieves the same MAE as that of the dark recording with zero spatial shift (point q_1_, right panel) but generates a dramatic decrease in the likelihood (point q_2_, left panel). The difference in the likelihood between point q_2_ and the zero spatial shift point of the dark recording is much larger than the difference between the likelihood values of light and dark zero spatial shift points. The MAEs under the null hypotheses (STAR Methods) for the light and dark trials are ∼ 68 and ∼ 66 cm, respectively. Error bars are ±SEM. (B) Same as (A) but with identical spatial shifts in all modules (full color traces; results for independent spatial shifts, same as in (A), are superimposed using faded colors for comparison). Under identical spatial shifts the dark and light likelihoods decrease only slightly (due to boundary conditions, left), whereas the corresponding MAE increases significantly in both cases (right). (C) Same as (A) but for another recording session (#25843) and with identical spatial shifts plotted in faded colors. The MAEs under the null hypotheses for the light and dark trials are ∼ 69 and ∼ 66 cm, respectively.

We found that the likelihood of simultaneously recorded spike trains in light trials was slightly higher than the likelihood in dark trials, although the MAE was much larger in darkness than in baseline light trials (Figure 5A, zero spatial shift). To demonstrate that the observed similarity in likelihoods was not simply an outcome of the rate-adjustment procedure, we also considered permuted, rate-adjusted spike trains, which preserved the mean firing rates. Under such a permutation, the evaluated likelihood decreased drastically and the MAE drastically increased (Figure S5A), demonstrating the importance of temporal structure of the simultaneous spike trains. To assess the significance of the small observed likelihood differences in relation to the question of module coordination, we evaluated the expected reduction in the likelihood under independent spatial shifts of varying magnitudes, as in Figures 4B and 4C. We found that the spatial shifts required to reduce the likelihood in the light to the value observed in the dark (point p_1_ in Figure 5A, left) would only generate a small increase in the MAE—of a few centimeters (point p_2_ in Figure 5A, right), whereas much larger spatial shifts would be required to match the actual MAE observed in the dark when using zero spatial shifts. Thus, the small reduction in the likelihood in the dark recordings at zero spatial shifts relative to the light is consistent with only small independent shifts across modules, of a few centimeters.

Conversely, the spatial shifts required to increase the MAE in the light to the value observed in the dark (point q_1_ in Figure 5A, right) would produce a substantial decrease in the likelihood, if applied in an uncoordinated manner to the spike trains from the light recordings (point q_2_ in Figure 5A, left), to a value that is much smaller than the zero-shift likelihood observed in the dark recordings. Thus, the increase in the zero-shift MAE observed in the dark recordings must arise mostly from coordinated drifts across the modules. To validate that coordinated drifts across modules can increase the MAE without significantly reducing the likelihood, we introduced identical spatial shifts across all modules as in Figures 4B and 4C. As expected, the likelihoods of dark and light trials were only slightly reduced, whereas the corresponding MAE’s increased substantially (Figure 5B). As in Figure 4B, the small reduction observed in the likelihoods with the introduction of coordinated spatial shifts is due to boundary conditions.

Results for additional datasets are shown in Figures 5C and S6A. Similar results were obtained when applying spatial rotations instead of spatial shifts (Figure S6B). Finally, we verified that differences in the motion statistics between light and dark trials are not expected to substantially affect the likelihood (Figure S5B). Movies showing typical examples of decoding from light and dark trials are shown in Videos S1 and S2.

Video S1. Markov decoding (light), related to Figure 5 and STAR Methods Posterior probability of position obtained using the Markov decoder (STAR Methods) during 30 representative seconds of the light trial from recording session #26018a. Time from trial onset is specified in the title. The animal’s true position is denoted with a black cross. The multi-module maximum likelihood estimate (MLE) is denoted with a red circle. Note that decoding performance is relatively good. Furthermore, the decoded position tends to slightly anticipate the animal’s movement, as expected from the prospective nature of the grid-cell position code (Kropff et al., 2015).

Video S2. Markov decoding (dark), related to Figure 5 and STAR Methods Same as Video S1 but for a corresponding darkness trial. Note that despite a relatively poorer multi-module decoding performance than in the light trial, the likelihood of the simultaneously recorded spike trains is similar to the likelihood evaluated in the light trial (Figure 5).

### Decoding from individual modules

The likelihood-based approach described above can be applied to datasets with moderate numbers of cells per module, where the decoded position is very noisy. In most of our datasets, the large number of simultaneously recorded cells enabled testing for inter-module coordination using a more direct approach, based on the decoding of position from individual modules.

**Figure 6:**
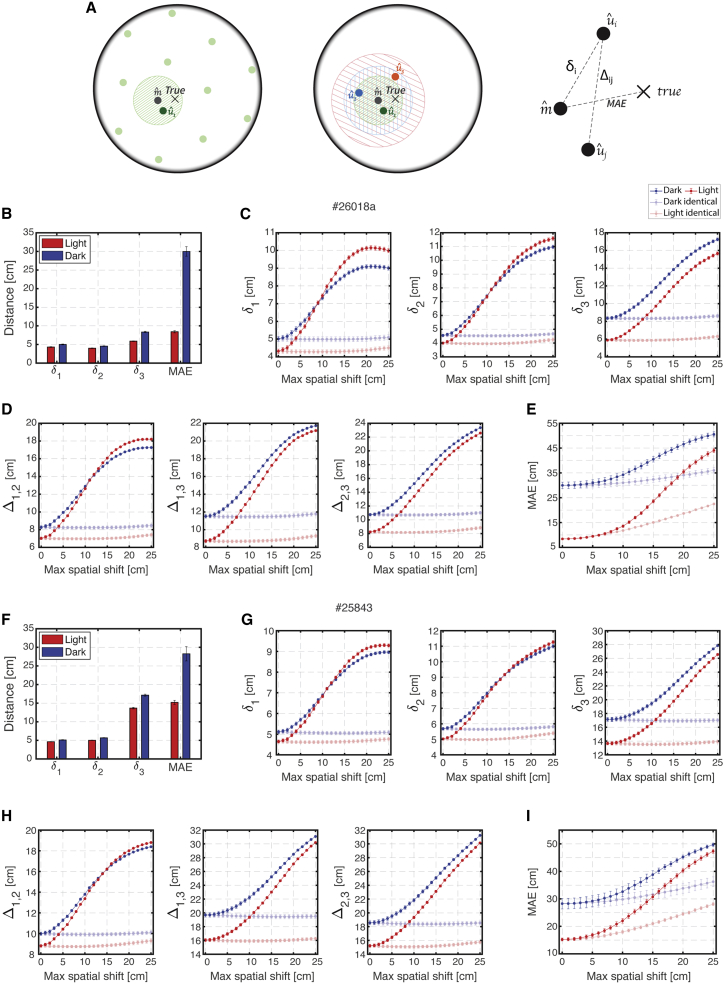
Decoding of population activity from individual modules reveals tight coordination of phases across modules in darkness (A) Schematic illustration of the uni-module decoding approach. Left: decoding the spiking activity from all grid cells typically produces a posterior with a unique blob in the arena, and the position of its maximum is chosen as the estimate of the multi-module internal representation (gray circle $\hat{m}$). This estimation can deviate from the true position of the animal (black X symbol). Decoding the spiking activity from a single module typically produces a periodic posterior (faded green circles). The blob that is nearest to position $\hat{m}$ is selected (dark green circle), and the position of its maximum is chosen as the estimate of the uni-module internal representation, denoted by ${\hat{u}}_{1}$. Middle: this procedure is repeated for each individual module (green, orange, and blue) producing an estimated uni-module position for each module (${\hat{u}}_{1}$, $\;{\hat{u}}_{2}$, and ${\hat{u}}_{3}$ ). Right: the distance from position $\hat{m}$ to the true position of the animal is averaged over time to produce the mean absolute error (MAE). The mean distance between position $\hat{m}$ to each position ${\hat{u}}_{i}$ is defined as $\delta_{i}$, and the mean distance between each pair of positions ${\hat{u}}_{i}$ and ${\hat{u}}_{j}$ is defined as $\Delta_{ij}$. (B) The measured MAE and distances $\delta_{i}$ for dark and light from a single recording session (#26018a). The distances $\delta_{i}$ in darkness are only ∼ 1 cm larger than those in baseline light trials. Error bars are ±SEM. (C) The distances $\delta_{i}$ for varying magnitudes of module-wise independent spatial shifts and for identical spatial shifts. Increasing the magnitude of module-wise independent spatial shifts increases $\delta_{i}$ dramatically, indicating that these measured distances $\delta_{i}$ could potentially be much higher. The same magnitude of identical spatial shifts has no effect. The $\delta_{i}$s under the null hypotheses (STAR Methods) for the light and dark trials are ∼ (13.7, 18.2, 25.5) cm and ∼ (13.8, 18.3, 25.7) cm, respectively. Error bars are ±SEM. (D) Same as (C) but for the corresponding distances $\Delta_{ij}$. (E) Similar as (C) but for the corresponding MAE. The MAE increases for both module-wise independent and identical spatial shifts. (F–I) Same as (B)–(E) but for another recording session (#25843). The $\delta_{i}$s under the null hypotheses for the light and dark trials are ∼ (16.2, 25.6, 32.7) cm and ∼ (16.4, 25.7, 32.8) cm, respectively.

In this approach, which is schematically illustrated in Figure 6A, an internal multi-module representation of position, denoted by $\hat{m}$, was first estimated by decoding spiking activity from all the grid cells. We used a *kernel decoder*, which estimates position based on spikes within a fixed time window, unlike the Markov decoder which has access to the entire spiking history (STAR Methods). As expected, the multi-module posterior typically exhibited a single prominent peak within the enclosure, which could potentially deviate from the true position of the animal (X in Figure 6A). Next, spiking activity from each module was decoded separately. As expected, single-module posteriors typically exhibited approximately periodic peaks. To resolve this ambiguity, the position which maximized the posterior within the local vicinity of the position $\hat{m}$ was selected as the corresponding decoded position ${\hat{u}}_{i}$ for each module i (STAR Methods). Finally, the distances between the position $\hat{m}$ to each position ${\hat{u}}_{i}$ (denoted by $\delta_{i}$) and between ${\hat{u}}_{i}$ pairs (denoted by $\Delta_{ij}$) were evaluated and compared between dark and baseline light trials (Figure 6A right). Typical examples of decoding from light and dark trials are shown in Videos S3 and S4.

Video S3. Uni-module decoding (light), related to Figure 6 and STAR Methods Log likelihood (posterior probability) of position obtained using the kernel decoder (STAR Methods) during 30 representative seconds of the light trial from recording session #26018a. Top left: multi-module decoding. Top right and bottom: uni-module decoding. Time from trial onset is specified in the title. The animal’s true position is denoted with a black cross. The multi-module maximum likelihood estimate (MLE) is denoted with a red circle. For each individual module (top right and bottom panels, see subtitles), the circular area (STAR Methods) around the multi-module MLE is denoted with a dashed gray circle, and the uni-module MLE within this circular area is denoted with a green star. Note that decoding performance is relatively good, and that the uni-module log likelihoods are dynamically coordinated. Furthermore, the decoded position may appear more temporally smooth than in Video S1, due to the choice of the kernel’s time scale. Nevertheless, the MAE of the Markov decoder is smaller than that of the kernel decoder (Figures 2D and 2E).

Video S4. Uni-module decoding (dark), related to Figure 6 and STAR Methods Same as Video S3 but for a corresponding darkness trial. Note that despite a relatively poorer multi-module decoding performance than in the light trial, the uni-module log likelihoods are dynamically coordinated in similarity to the light trial (Video S3).

The MAE of position $\hat{m}$, with respect to the animal’s true position, was higher in darkness than in baseline light trials (one example shown in Figure 6B), indicating that the internal representation of position in the dark drifted relative to the true position. The distances $\delta_{i}$ and $\Delta_{ij}$ were noisy and fluctuated in time both in dark and light conditions, but their mean was only slightly higher in darkness than in light (Figure 6B). This is consistent with the results from the previous approach (Figure 5), which demonstrated slightly higher likelihoods for simultaneously recorded light spike trains compared with corresponding dark trials.

Since the single-module readout positions ${\hat{u}}_{i}$ were restricted to a vicinity of the position $\hat{m}$, which, by definition, best agrees with activities from all modules, it was important to verify that the similarity of distances in light and dark trials was not an inevitable consequence of the methodology. Therefore, we introduced independent spatial shifts in the rate maps of all neurons that belong to the same module during the decoding process in a similar fashion as performed in the previous likelihood approach. We found that the mean distances $\delta_{i}$ and $\Delta_{ij}$ increased dramatically as the magnitude of spatial shifts increased, indicating that these measured distances could have potentially been much higher than observed and did not arise simply because of the selection of positions ${\hat{u}}_{i}$ in proximity to $\hat{m}$ (Figures 6C and 6D). Therefore, the preservation of these small distances in the dark, while the position $\hat{m}$ deviated substantially from the animal’s true position (Figure 6E), is an explicit indication of tight coordination between the modules. As expected, identical spatial shifts did not affect $\delta_{i}$ and $\Delta_{ij}$, although they dramatically increased the MAE (faded traces in Figures 6C–6E). Similar results from additional datasets are shown in Figures 6F–6I and S7.

**Figure 7:**
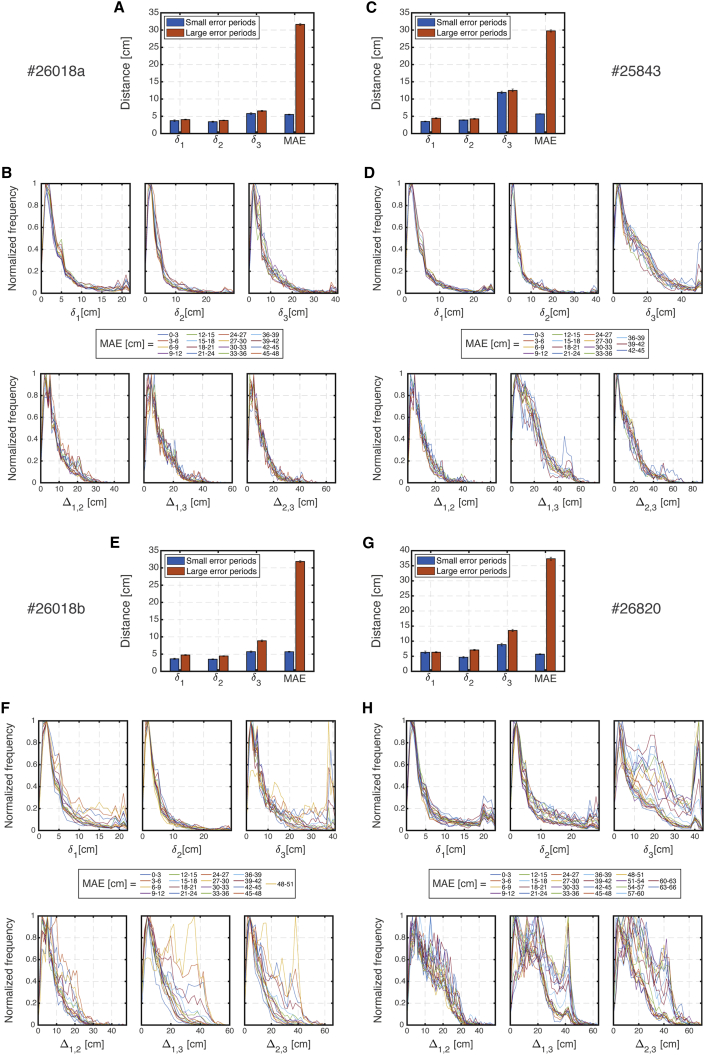
Module population activity patterns are tightly coordinated even during high-MAE periods (A) The measured mean absolute error (MAE) and distances $\delta_{i}$ during large- and small-MAE periods from a single recording session (#26018a) during darkness. Error bars are ±SEM. (B) Top: corresponding normalized distributions of the distances $\delta_{i}$ for varying values of the MAE. Bottom: same as top but for distances $\Delta_{ij}$. (C and D) Same as (A) and (B) but for another recording session (#25843). Note that outliers in (D) are traces with largest MAE. (E–H) Same as (A) and (B), for two additional recording sessions (#26018b and #26820) in which recordings were obtained from three modules. Note that, although recordings from three modules were available in these datasets, the numbers of simultaneously recorded grid cells were relatively small (especially in recording session #26820; Table 1), leading to inaccurate decoding.

We finally tested whether module coordination remained tight in darkness, specifically during periods in which the multi-module readout $\hat{m}$ deviated substantially from the true position of the animal. To address this question, we focused on non-overlapping continuous segments of the dark recordings in which the MAE was particularly high and on non-overlapping continuous segments within the same recording in which the MAE was particularly low (STAR Methods). Although the deviation of the internal representation from the true position was in the order of ∼ 30 cm in the high-MAE segments (compared with order of ∼ 5 cm in the low-MAE segments), the distances $\delta_{i}$ remained small as in the low-MAE segments (Figure 7A). Importantly, these distances $\delta_{i}$ could have potentially been much higher, as demonstrated above (Figures 6C and 6D). This result indicates that representations within individual modules did not accrue any additional, significant relative drifts even when the multi-module representation of position deviated substantially from the animal’s true position.

Furthermore, we considered all time points from dark trials and examined the joint distributions of $\delta_{i}$ and the MAE and the joint distributions of $\Delta_{ij}$ and the MAE. We expected that if modules are coordinated then the conditioned distributions of $\delta_{i}$ and $\Delta_{ij}$ will be nearly independent of the MAE and in particular remain narrowly distributed even when the MAE is large. Figure 7B demonstrates that, indeed, the distances $\delta_{i}$ and $\Delta_{ij}$ were distributed nearly identically for different values of the MAE. This is yet another explicit indication that coordination between modules remains tight, even when the internal representation of position deviates from the true position of the animal. Results for additional datasets are shown in Figures 7C–7H.

## Discussion

In contrast to the rigid relationships in activity of cells within a module, activity in different modules spans diverse phase combinations, allowing them to represent a large range of positions and environments. Nevertheless, here we showed that dynamically, the phases of different modules are coupled. Even when the internal representation of position in the MEC deviates substantially from the true position of the animal, updates to the phases remain coordinated across the different modules, thus maintaining a coherent representation of a two-dimensional trajectory.

The likelihood-based approach and the uni-module decoding approach both led to the conclusion, consistently across different animals and sessions, that the inter-module phases are dynamically coupled. Both methods also pointed to a small increase in the mismatch between modules in darkness compared with light conditions, indicating that sensory inputs help coordinate the states of different modules. The picture that emerges from these results is that when sensory inputs are poor or absent, small mismatches can develop in the phases of different modules, but internal brain mechanisms prevent these mismatches from accruing over time, thus maintaining a coordinated and coherent representation across the full grid-cell population. It has been previously hypothesized that such internal mechanisms may exist (Burak, 2014; Welinder et al., 2008), possibly supported by recurrent synaptic connectivity within the MEC (Kang and Balasubramanian, 2019; Mosheiff and Burak, 2019) or by the reciprocal synaptic connectivity of the MEC with the hippocampus (Agmon and Burak, 2020; Sreenivasan and Fiete, 2011; Welinder et al., 2008). It will be of great interest to explore these underlying mechanisms in future studies, for example, by testing whether inputs from the hippocampus are required to maintain phase coordination.

The analyses of the recorded spike trains relied on simplifying assumptions. Grid cells are modeled as independent units, which emit Poisson spike trains that are solely dictated by their spatial selectivity, alongside a random walk prior (in the likelihood approach). However, the actual mechanisms that govern grid-cell activities are more complex. Therefore, the decoders are not optimal (or tailored) for the actual recorded data. Note, however, that we do not compare decoding performance of simulated and recorded spike trains, but instead compare the decoding performance of recorded light and dark spike trains. This enables us to reliably quantify and compare the coherence of recorded population activities across the dark and light conditions. Our spatial shift controls, which induce inter-module incoordination, are directly generated from the recorded data and thus do not require a comparison of the quantified coherence from the recorded data with that obtained from simulated spike trains. Altogether, these analyses indicate that internal brain mechanisms enforce coordination between distinct grid-cell modules.

Despite a major reduction of sensory cues, their elimination was probably not complete. Although the arena was carefully cleaned during and between trials, leftover olfactory cues may have provided some spatial information. The rare encounters with the walls could also provide limited spatial information (Hardcastle et al., 2015); yet, it is unlikely that absolute position could be inferred from such encounters, as the arena was rotationally symmetric. Although the elimination of sensory cues was probably imperfect, the disruption of the spatial rate maps and the increased MAE of decoded population activities in darkness (Figure 2) demonstrated that a large mismatch between internal representation and true position was typical under these conditions. Importantly, the typical mismatch was in the order of tens of centimeters, whereas the misalignment between modules was in the order of a few centimeters, even during periods in which the MAE was particularly large (Figure 7). The likelihood approach (Figure 5) points to similar conclusions because uncoordinated spatial shifts of more than a few centimeters applied onto the dark recording have led to a large decrease in its likelihood, relative to the baseline difference between the light and dark likelihoods.

Previous studies that examined grid-cell activity in mice in dark environments (Chen et al., 2016; Pérez-Escobar et al., 2016) have shown that even when the periodic firing patterns of grid cells lose their spatial stability, grid cells within a module preserve the pairwise spiking correlations that they exhibited in the light. However, these works did not test whether the coactivation patterns of distinct modules are coordinated during darkness. Due to the recording technique (classical tetrodes), simultaneously recorded cell pairs from distinct modules were rare, and the data collected did not enable analysis based on population decoding as performed in the present work.

The spatial response patterns of individual grid cells in the mouse studies (Chen et al., 2016; Pérez-Escobar et al., 2016) were more strongly degraded in darkness than those observed in rats in our study (Figures 2B and 2C). These differences might possibly emerge from different dead-reckoning capabilities of rats and mice: unlike mice, rats have been shown to exhibit stable grid fields in darkness (Hafting et al., 2005). Consequentially, it was necessary in our study to take extreme measures (large circular arena, removing odors) to obtain strong degradation of the grid response patterns. The decoding results (Figures 2D and 2E) confirmed that our rats were indeed disoriented in darkness, even when the dark data were decoded from dark-generated rate maps (Figures S3B and S3C). Previous work in the hippocampus (Bjerknes et al., 2018) has demonstrated excellent ability of place cells to express precisely localized fields on a linear track in darkness, under conditions that ensured absence of any relevant external sensory information. Therefore, it is possible that with scarce remaining sensory cues, rats can maintain spatial maps—in hippocampus and MEC—more accurately than mice. Another possibility is that differences in the degradation of grid responses result from subtle differences in the environments or training protocols used by the mouse and rat studies. However, the origin of these differences in the precision of spatial representation during darkness is not the subject of this study. What is crucial for the study of coordination between grid-cell modules is that substantial disorientation was achieved.

Previous work (Stensola et al., 2012) pointed to a functional independence in the response of modules to an abrupt environmental deformation (Barry et al., 2007) in which the enclosure was compressed by moving one of the walls. A key finding was that this manipulation resulted in compression of the rate maps that occurred in some modules but not in others. Thus, the distinct responses of different modules to the environmental deformation were indicative of functional independence in their dynamics. Nevertheless, the implications of this result for the dynamics of module coordination are not yet sufficiently clear: one possibility is that even shortly after the environmental deformation, grid-cell firing remains anchored to position. In this case, the rates of phase updates, as a function of position, are modified compared with baseline conditions. Under this interpretation of the experiment, the dynamical coordination of modules is disrupted everywhere within the enclosure, possibly transiently. Alternatively, it has been suggested (Keinath et al., 2018; Ocko et al., 2018) that module phases are updated abruptly upon encounters with the walls due to interactions with border cells. Under this interpretation of the experiment, rate maps are altered due to spatial shifts of the grid firing fields that depend on recent encounters with the walls. Yet, the phase update rates remain largely unmodified within the interior of the environment, and dynamical module coordination remains intact between encounters with the walls. Based on the tetrode recordings that were available in the deformation experiment (Stensola et al., 2012), it is difficult to conclusively distinguish between these possibilities. To do so, it will be beneficial to decode module phases from population activity patterns and analyze their joint dynamics, utilizing large numbers of simultaneously recorded grid cells from different modules.

The analysis in this work relied on the ability to simultaneously record spikes from dozens to hundreds of grid cells and our results demonstrate the power of this technique in elucidating dynamics within large neural circuits (Chaudhuri et al., 2019; Gallego et al., 2018; Gardner et al., 2022; Pfeiffer and Foster, 2013). With several dozens of cells from each module, decoding of phases from single modules was sufficient to obtain strong measures of coordination between the modules, based on statistics that were collected across long periods of motion. Future studies, with even larger numbers of simultaneously recorded cells, may enable more precise dynamical tracking of the states of individual modules over single trials. Such analysis may include extraction of toroidal coordinates from the joint activity in each module independently of the spatial selectivity (Gardner et al., 2022), which requires more simultaneously recorded cells than was available for most modules in the present study. With such finer temporal and spatial resolution, it may be possible to characterize more precisely how the small mismatch that does exist between modules evolves over time, in relation to behavior or external stimuli. Such analysis may further elucidate the mechanisms that underlie coordination between attractor networks in the entorhinal cortex and the hippocampus.

## STAR★Methods

### Key resources table

**Table:** 

REAGENT or RESOURCE	SOURCE	IDENTIFIER
**Experimental Models: Organisms/Strains**		

Rat: Long-Evans	Bred inhouse at KISN	Kavli Institute for Systems Neuroscience

**Software and Algorithms**		

Matlab 2020a	MathWorks	https://mathworks.com/products/matlab.html RRID: SCR_001622
UMAP	MathWorks	https://www.mathworks.com/matlabcentral/fileexchange/71902
DBSCAN	MathWorks	https://mathworks.com/help/stats/dbscan.html
SpikeGLX	Jun et al., 2017	https://billkarsh.github.io/SpikeGLX/
Kilosort	Steinmetz et al., 2021	https://github.com/MouseLand/Kilosort
Phy	Jun et al., 2017	https://github.com/cortex-lab/phy
DBCV	Moulavi et al., 2014	https://imada.sdu.dk/∼zimek/publications/SDM2014/
OptiTrack Motive	OptiTrack	https://optitrack.com/software/motive/

**Other**		

Neuropixels Probes	Neuropixels	https://www.neuropixels.org/
Neuropixels Control System	Neuropixels	https://www.neuropixels.org/control-system
Kintex-7 FPGA board	Xilinx	https://www.xilinx.com/products/boards-and-kits/device-family/nav-kintex-7.html
Optitrack Flex 13 USB cameras	OptiTrack	https://optitrack.com/cameras/
Zeiss AxioImager	Zeiss	N/A

**Deposited Data**		

Datasets	This paper	https://doi.org/10.5281/zenodo.6200517

### Resource availability

#### Lead contact

Further information and requests for resources should be directed to and will be fulfilled by the lead contact, Prof. Yoram Burak (yoram.burak@elsc.huji.ac.il).

#### Materials availability

This study did not generate new unique reagents.

### Method details

#### Subjects

Experimental testing took place at the Kavli Institute for Systems Neuroscience, NTNU, Norway. Data were obtained from 4 male Long Evans rats (300-500 grams when implanted, at ages P 73-107 days old at day of recording). After weaning at three weeks, the rats were group-housed with their siblings until the implantation date. After implantation, each rat was housed alone in a large two-story enriched metal cage (95 x 63 x 61 cm). The rats were kept in temperature and humidity controlled rooms on a 12 hr light / 12 hr dark schedule. Experiments took place in the dark phase of the schedule. All procedures were performed in accordance with the Norwegian Animal Welfare Act and the European Convention for the Protection of Vertebrate Animals used for Experimental and Other Scientific Purposes.

#### Electrode implantation surgery

The rats were implanted with single-shank 384-site Neuropixels probes (Jun et al., 2017) targeting the medial entorhinal cortex (MEC) in either one or both hemispheres. Rat #26018 was implanted only in the right hemisphere, while rats #25843 and #26820 were implanted bilaterally with prototype Neuropixels 'phase 3A' probes. Rat #26718 was implanted with a Neuropixels 1.0 probe in the right hemisphere. Before implantation, the rats were anaesthetized with isoflurane in an induction chamber and given subcutaneous injections of buprenorphine (Temgesic) and Meloxicam (Metacam). They were then fixed in a Kopf stereotaxic frame with continuous isoflurane administered through a mask. Local analgesic bupivacaine (Marcaine) was injected subcutaneously before making the incision. Craniotomies were drilled above the MEC area. The probes were inserted at a maximum depth of 5-6 mm from the brain surface, 4.4-4.6 mm lateral to the midline suture, 0.1-0.3 mm anterior to the transverse sinus, at angles between 25-26 degrees from the vertical plane, with the tip of the probe pointing in the anterior direction. A single jewellers screw was secured through the skull above the cerebellum and connected to the probe ground with an insulated silver wire. The implants were secured in place with dental adhesive (Optibond from Kerr) and Venus composite (Kulzer) and protected by fitting a modified falcon tube. Postoperative analgesia (meloxicam and buprenorphine) was administered during the surgical recovery period.

#### Electrophysiological recordings

Electrophysiological signals were recorded with a Neuropixels acquisition system as described previously (Gardner et al., 2022; Jun et al., 2017). The spike band signal was recorded and amplified with a gain of 500, filtered to keep a bandwidth from 0.3 to 10 kHz and then digitized at 30 kHz on the probe circuit board. The signal was further multiplexed and transmitted to a Xilinx Kintex 7 FPGA board (‘phase 3A’) or a Neuropixels PXIe acquisition module (1.0) via a 5 m tether cable before being streamed via ethernet connection to a local computer. Rat #26018 had two recording sessions: recording session #26018a was performed 4 days before recording session #26018b, with partial overlap of recorded cells between the two sessions.

#### Behavioural tracking

During recording, a rigid body with five retroreflective markers was attached to the rat’s implant and tracked with a 3D motion capture system (six OptiTrack Flex 13 cameras and Motive software) at ∼ 120 Hz. To synchronise the timestamps of the two recording systems, randomized sequences of digital pulses generated by an Arduino microcontroller were sent to both the Neuropixels acquisition system as direct TTL input and to the OptiTrack system via infrared LEDs placed on the edge of the arena.

#### Behavioural procedures

The rat’s movement was tracked as it moved freely in a circular open field arena. The recording arena was a 150 cm diameter, matt black plastic cylinder with 50 cm high walls and a matt black hard rubber floor, surrounded by floor-to-ceiling dark blue blackout curtains on all sides ∼ 1m from the arena edge. Three additional layers of blackout curtains separated the recording arena from the part of the room with the recording computer. The same behavioural arena was used in both darkness and light recordings.

#### Open-field foraging trials in darkness

Complete darkness was ensured by turning off all potential sources of light in the recording room. Light sources which could not be turned off were masked with aluminium foil and electrical tape and/or blackout curtains. Before starting the experiment, the arena and floors were thoroughly cleaned with soap water and dried. The rat’s Neuropixels probe was connected to the recording system outside the closed curtains before the final lights were shut off, and the rat was introduced to the recording arena at an arbitrary position and direction. For 50-60 minutes, the rat was left to freely explore the arena and forage small pieces of corn foam snack thrown into the arena during the trial by an experimenter wearing night-vision goggles (Armasight Nyx-7 pro). To avoid delivery of systematic orientational cues, the experimenter accessed and left the ring of curtains from random locations, and quickly dropped food pellets and removed excrement and urine using a paper towel while the rat was in a different location in the arena. Light conditions were not changed during these times.

#### Open-field foraging task in light

After the open foraging task in darkness, while the rat was still foraging in the arena, or with a short break to untwist the Neuropixels tether cables, the experimenter turned on the light and continued the recording for another 30-60 minutes. During the light task, a single white textile cue card (∼ 45 cm wide, ∼ 150 cm high) hanging on the blue curtains outside the arena was visible from within the arena. The only light source in the light task was a single LED strip (6 m, 120 LEDs, 2800K color temp) placed as a uniform ∼ 2 m diameter ring directly above the arena at a height of ∼ 2.8 m, evenly illuminating the arena and ensuring no shadows were cast on the floor.

#### Perfusion and histology

The rats were anaesthetized with isoflurane in an induction box and given a lethal injection of pentobarbital. When unresponsive, the rats were perfused transcardially with 0.9% saline, followed by 4% Formalin solution. The brain was extracted and stored in 4% Formalin solution for at least 24 hours before being sliced in 30 μm sagittal sections on a cryostat. The brain sections were stained with Cresyl Violet, and photomicrographs were taken through a Zeiss Axio Imager.

#### Spike sorting and single-unit selection

Spike sorting was performed with a version of KiloSort 2.5 (Steinmetz et al., 2021), optimised for MEC/PaS recordings as described in Gardner et al. (2022), including manual supervision of cluster split and merge processes. Single units were excluded from further analysis if more than 1% of intervals in their interspike interval distribution were shorter than 2 ms or if they had less than 500 total spikes in the light task.

#### Module classification

Grid cell and module classification was done by vectorizing the spatial autocorrelation from the rate map of every cell and adding them as feature columns in a matrix used as input to the UMAP (Uniform Manifold Approximation and Projection) dimensionality reduction algorithm (McInnes et al., 2018), before DBSCAN (Ester et al., 1996) was used to assign cluster identities to the resulting 2D point clouds, as in Gardner et al. (2022) (Figures 1E, 1F, and S2A–S2C). Briefly, for each recorded cell, rate maps were generated by dividing the arena into 8-10 cm bins and counting the number of spikes within each bin divided by the time spent in that bin. Autocorrelograms of the rate maps were calculated, and values from the bins in a circular area within a 3-bin radius from the center bin were removed along with the bins outside a radius defined by the edge of the matrix. The autocorrelograms were then vectorized and used as features in UMAP used to project the values down to a point cloud in 2 dimensions. DBSCAN was used to cluster the points, which yielded a single large cluster with non-grid cells and single clusters for each module (identified by a clear grid pattern and high gridness score in the mean autocorrelogram of each cluster); grid modules could be ordered by the grid spacing and orientation calculated from the mean autocorrelogram of each cluster. Of all recorded cells, only those from clusters with a clear grid pattern in the mean autocorrelogram, and a validity index (see clustering validation section) close to 1.0 were used for further analysis. Grid pattern classification was defined as follows: for each cluster, we took the mean of the gridness scores of all cells in the cluster. We considered the clusters which had a high within-cluster mean gridness score of order 1, as grid clusters (M1, M2, M3), and clusters with a low within cluster mean gridness score (close to 0) as non-grid. The lowest mean gridness score in a cluster classified as a grid-cell cluster was 0.66 (M2 in session #26718) and the highest mean gridness score in a cluster classified as “non grid-cell” was 0.13 (session #26018b). The mean, SD, and SEM of the gridness scores for each cluster and for all recording sessions are shown in Figure S2F.

#### Clustering validation

The grid cell and module classification results were validated by calculating a density-based clustering validation (DBCV) index (Moulavi et al., 2014) for each DBSCAN-assigned cluster identity in the 2D UMAP point cloud (Figures 1E, 1F, and S2A–S2C). The DBCV index has a range of -1 to 1, where a cluster gets a positive value if the lowest density region inside the cluster is higher than the highest density in the region that separates it from other clusters (Figures S2G–S2K).

#### Rate map analysis

The firing rate $\lambda_{i}$ [Hz] of grid cell i at each position $\vec{x}$ in the arena was generated as follows:

$$\lambda_{i}\left(\vec{x}\right)=\frac{\sum_{j=1}^{\nu_{i}}g\left({\vec{x}}_{i}^{j}-\vec{x}\right)}{\Delta t\sum_{t=1}^{T}g\left({\vec{y}}_{t}-\vec{x}\right)}$$

where $\nu_{i}$ is the total number of spikes emitted by neuron i, and ${\vec{x}}_{i}^{j}$ is the animal’s position when spike j was emitted. The position of the animal at time t is denoted by ${\vec{y}}_{t}$, and g is a two-dimensional Gaussian kernel with diagonal covariance matrix $\sum_{ii}=25\;{\text{cm}}^{2}$. Spike trains and tracking data were binned at $\Delta t=\frac{1}{120}\;\text{s}$ resolution, and only time bins where the animal was moving at a speed greater or equal to $3\;\frac{\text{cm}}{\text{s}}$ were used for spatial analyses.

#### Gridness score

The gridness score was computed to measure the degree of hexagonal spatial periodicity, as in Langston et al. (2010). For each cell, an autocorrelogram was calculated from its rate map and rotated in five steps of 30 degrees, correlating each rotated matrix with the original autocorrelation in the following manner: First, the values correlated were restricted to a ring of bin indexes around the center peak of the autocorrelogram; then, this ring of bins was expanded stepwise until its outer edge reached the edge of the autocorrelogram matrix. For each step, a score was calculated as the difference between the lowest correlation at [60, 120] degrees and the highest correlation at [30, 90, 150] degrees. The gridness score was taken as the mean of the three scores surrounding and including the step with the maximum score, resulting in a theoretical score range of [-2, 2].

#### Information content

The spatial information content [bits/spike] (Skaggs et al., 1996) of neuron i is defined as

$$\sum_{i=1}^{N}p_{i}\;\frac{\lambda_{i}}{\lambda }\;\log_{2}\left(\frac{\lambda_{i}}{\lambda }\right)$$

where $\lambda_{i}$ is the unit's mean firing rate in the i-th bin of the rate map, λ is the overall mean firing rate and $p_{i}$ is the probability of the animal being in the i-th bin (time spent in the i-th bin divided by the duration of recording).

#### Pairwise correlations

Spike trains were binned at $\Delta t=\frac{1}{120}\;\text{s}$ resolution and smoothed using a Gaussian kernel with σ=50ms. Pearson correlation coefficients were then calculated for pairs of spike trains from simultaneously recorded grid cells.

In Figures 3B and 3D neurons were divided into ten equally sized groups with equal distributions of neurons from each module and cross-correlations were calculated across inter- and intra-module pairs that belong to the same group to obtain independent evaluations. Cross-correlations were calculated by iteratively lagging one of the spike trains relative to the other. To avoid cancellations of positive and negative contributions from intra-modular cell pairs with different phase relationships, we averaged the absolute magnitude of the correlations over pairs within each of the independent groups.

In Figures 3C, 3E, and S4B, the Pearson correlation coefficients were calculated for all possible inter- and intra-module pairs without lagging any of the spike trains.

#### Markov decoder

The Markov decoder updates its posterior likelihood for position r as follows:

(Equation 2) $$p\left(r;t+\text{Δ}t\right)=\frac{1}{Z\left(t\right)}\left[\int p\left(r^{\text{′}};t\right)p_{D}\left(r|r^{\text{′}}\right)\text{d}r^{\text{′}}\right]p_{S}\left(r;t\right)$$

where $p_{D}\left(r|r^{\prime }\right)$ describes the animal’s probability to run from location $r^{\prime }$ to location r during time interval Δt. The term $p_{S}\left(r;t\right)$ is the probability for all the neurons to emit the observed spikes within the time interval Δt, given the position r. The posterior likelihood is iteratively normalized by Z(t).

Explicitly, $p_{D}\left(r|r^{\prime }\right)$ is a two-dimensional Gaussian distribution centered around position $r^{\prime }$ with diagonal covariance matrix $\sum_{ii}=4\;{\text{cm}}^{2}$. The time step, $\Delta t=\frac{1}{120}\;\text{s}$, is equal to the sampling rate of the data. Thus, the diffusion coefficient is $D=480\;\frac{{\text{cm}}^{2}}{\text{s}}$, which was chosen to roughly minimize the MAE in the light recording sessions.

The posterior extracted from spiking activity was evaluated assuming independent Poisson firing, namely

$$p_{S}\left(r;t\right)=\prod_{i=1}^{N}\frac{1}{n_{i}!}{\left(f_{i}\text{Δ}t\right)}^{n_{i}}\text{exp}\left(-f_{i}\text{Δ}t\right)$$

where $f_{i}\left(r\right)$ is the tuning curve and $n_{i}$ is the spike count of the i'th neuron during the time interval Δt.

Finally, the estimate of position is the maximum likelihood estimate

$$\hat{r}=\underset{r}{\text{argmax}}\;p\left(r;t\right)$$

#### Likelihood of simultaneously recorded spike trains

The probability for observed spike trains $p\left(S_{t}\right)$ is given in Equation 1. By exploiting the Markov decoder’s properties, the likelihood for simultaneous observed spikes turns out to be simply proportional to the multiplication of its iterative normalization factors presented above,

$$p\left(S_{t}\right)=\prod_{i=1}^{t}Z_{i}$$

A detailed analytical derivation is given in Methods S1.

We present in Figures 4 and 5 the average log likelihood per time unit defined as

(Equation 3) $$L={\left\langle \log\left(Z\right)\right\rangle }_{t}$$

Since decoding was Markovian we decoded all the data, but only time bins where the animal was moving at a speed greater or equal to $3\;\frac{\text{cm}}{\text{s}}$ were used for further analyses.

#### Rate-adjusted likelihood

In order to evaluate the likelihood using the Markov decoder and faithfully compare this quantity across light and dark conditions, it is necessary to make concrete assumptions on the neural tuning curves. Since individual grid cells differed in their firing rates in these two conditions, it was necessary to analyze how the firing rate influences the likelihood, and then compensate for this influence. Counter intuitively, the evaluated likelihood decreases as the number of total spikes in the recording increases (see Methods S1 for an analytical derivation which elucidates this finding).

Our assumption is that neurons maintain the same underlying structure of tuning curves in light and dark conditions, up to a scaling factor that adjusts the firing rate (see also Methods S1; Figure S8). Therefore, we matched the mean firing rates by randomly omitting spikes in the condition in which the firing rate was higher, yielding spike trains from each neuron with the same mean firing rate in the dark and light conditions. This was done separately for time bins where the animal was moving at a speed smaller than $3\;\frac{\text{cm}}{\text{s}}$, and for time bins where the animal was moving at a speed greater or equal to $3\;\frac{\text{cm}}{\text{s}}$ since these time bins were used for further analysis.

#### Mean absolute error (MAE)

The MAE is defined as the average Euclidean distance between the decoded position and the true position of the animal.

#### Spatial shifts

For each module, a spatial shift was chosen at random from the range [−α,α], independently for each of the two spatial dimensions (horizontal and vertical). These shifts were fixed throughout the duration of each simulation. The variable α is plotted as the ‘Max spatial shift’ axis in the figures throughout this article. Rate maps of all neurons that belong to the same module were shifted according to their corresponding random shifts, thus producing shifted rate maps. Rate maps were set to zero in positions outside of the arena. Firing rates at new positions that were included within the arena boundaries only after the shift but were outside of the arena boundaries before the shift were set to zero. Identical spatial shifts were applied in a similar procedure, but only a single set of two-dimensional shifts were chosen at random and were applied to the rate maps of all neurons as described above, regardless of the module they belong to. Thirty such independent realizations were simulated for each ‘Max spatial shift’ value.

#### Rotational Shifts

For each module, a spatial rotation angle was chosen independently and at random from the range [−α,α]. These shifts were fixed throughout the duration of each simulation. The variable α is plotted as the ‘Max spatial rotation’ axis in Figure S6B. Rate maps of all neurons that belong to the same module were rotated with respect to the arena’s origin, according to their corresponding random angle, and thus producing rotated rate maps. Identical rotational shifts were applied in a similar procedure, but only a single angle was chosen at random and was used to rotate the rate maps of all the neurons as described above, regardless of the module they belong to. Thirty such independent realizations were simulated for each ‘Max rotational shift’ value.

#### Idealized grid cell tuning curves

The idealized grid cells had the same allocation to modules as in the recording sessions (Table 1) mentioned in Figures S4B and S4F captions, with corresponding grid spacings of $\vec{\lambda }=\left[45,65,95\right]\;\text{cm}$. Tuning curves were modeled as a sum of Gaussian blobs whose peaks lie on a perfect hexagonal lattice. In each module, the Gaussian blobs had a diagonal covariance matrix $\sum_{ii}=0.015\cdot \lambda^{2}\;{\text{cm}}^{2}$. The peak firing rate was 30 Hz. Phases of cells from the same module were uniformly distributed, and the angular orientation of each module was independently and randomly chosen.

#### Kernel decoder

The kernel decoder updates its posterior likelihood for position r based on recent emitted spikes, weighted exponentially (Mosheiff et al., 2017). It is straightforward to express the log likelihood of spike counts $\nu_{i}$, observed within a temporal window of duration Δt, as a function of the position r:

(Equation 4) $$\log\;p\left(\left\{\nu_{i}\right\}|r\right)=\sum_{i}f\left(r-r_{i}\right)\text{Δ}t+\sum_{i}\nu_{i}\;\log\left\{f\left(r-r_{i}\right)\text{Δ}t\right\}-\sum_{i}\nu_{i}!=c+\sum_{i}\nu_{i}\;\log\;f\left(r-r_{i}\right)$$

where c is a constant that does not depend on r. The term $\sum_{i}f\left(r-r_{i}\right)$ contributes only to this constant because of the assumption of dense, transnationally invariant receptive fields with uniform distribution. The index i runs over grid cells: either all the cells to produce the multi module posterior, or on all the cells within a module to produce the corresponding uni-module posterior. A maximum likelihood estimator for r (assuming uniform prior) will chose

$$\hat{r}\left(\left\{\nu_{i}\right\}\right)=\underset{r}{\text{argmax}}\sum_{i}\nu_{i}\;\log\;f\left(r-r_{i}\right).$$

The spikes from recent history are weighted with a temporal kernel h(t). Thus, we generalize $\nu_{i}$ to:

$$\nu_{i}=\int_{-\infty }^{t}h\left(t-t^{\prime }\right)\xi_{i}\left(t^{\prime }\right)\text{d}t^{\prime }.$$

Here, $\xi_{i}\left(t\right)$ is a series of delta functions that represents the spike from neuron i, and

$$h\left(t\right)=\text{exp}\left(-\frac{t}{\tau }\right)$$

where we used τ=100ms. In Equation 4 Δt is replaced by τ.

Only time bins where the animal was moving at a speed greater or equal to $3\;\frac{\text{cm}}{\text{s}}$ were used for analyses.

#### Uni-module decoding

As expected, the posterior was approximately periodic when activity was decoded from single modules. To remove ambiguity, the decoded position ${\hat{u}}_{i}$ of each module i was defined as the position that maximized the posterior within a circular area. The circular area had a diameter equal to ∼ 90% of the corresponding module spacing and was centered around the multi-module position $\hat{m}$ (Figure 6A). Thus, only a single blob was included within the circular area around the multi-module represented position $\hat{m}$.

#### Small and large dark error periods

In Figures 7A, 7C, 7E, and 7G the error between the joint kernel decoded position and true position during darkness was first temporally smoothed using a Gaussian kernel with σ=50ms. Periods with particularly small error are defined as continuous non-overlapping segments spanning at least 1 s with a maximal smoothed error (SE) of 10 cm. Periods with particularly large error are defined as continuous non-overlapping segments spanning at least 1 s with a minimal SE of 20 cm, and a maximal SE which was determined as follows: the maximal SE was chosen as the value corresponding to 80% of the cumulative distribution of the error between the joint kernel decoded position and true position. Time points with errors larger than this cutoff were discarded from the analysis in accordance with the cutoff used in Figures 7B, 7D, 7F, and 7H (largest MAE value shown in legend) due to sparseness of the joint distribution with the distance between ${\hat{u}}_{i}$ and $\hat{m}$ (= $\delta_{i}$). Recording session #25843 had 257 large-error segments and 305 small-error segments. Recording session #26018a had 426 large-error segments and 126 small-error segments. Recording session #26018b had 460 large-error segments and 156 small-error segments. Recording session #26820 had 384 large-error segments and 92 small-error segments.

#### Null hypotheses of MAEs and $\delta_{i}$s

The null hypothesis for the MAE was evaluated for the light and dark trajectories in each recording session (as specified in Figures 5 and S6A captions). It is defined as the mean of Euclidean distance between a randomly chosen position and the true position of the animal in the arena. Thirty realizations have been simulated for each recording session and illumination condition, yielding SEMs in the order of $10^{-2}$ cm.

The null hypotheses for the $\delta_{i}$ s (as specified in Figures 6 and S7 captions) are defined similarly, but the distances were calculated between the multi-module decoded position and a randomly chosen position within the corresponding module’s circular area as defined above (*Uni-module decoding*). Thirty realizations have been simulated for each recording session and illumination condition, yielding SEMs in the order of $10^{-3}$ cm.

#### SEM of correlated time series

Whenever the standard error of the mean (SEM) of a single time series signal (S) was evaluated, correlations were taken into account by updating the signal’s variance based on the auto-correlation function (ACF(S)).

For an independent signal the variance is simply $ACF_{0}$. However, for a temporally correlated stationary signal, the actual variance is written as

$$\text{Var}\left(S\right)=ACF_{0}+\sum_{i=1}^{\infty }2\cdot ACF_{i}$$

where in practice the cutoff point of the sum remains to be determined. We trimmed the sum at a point corresponding to an autocorrelation value satisfying $ACF_{i}\;\leq \;0.15\cdot ACF_{0}$. Finally, the SEM is defined as $\text{SEM}=\sqrt{\text{Var}\left(S\right)/n}$ where n is the total number of points in the signal.

## Data Availability

The data are available at Zenodo (https://doi.org/10.5281/zenodo.6200517). The code is available at Zenodo (https://doi.org/10.5281/zenodo.6208720).

## Supporting citations

The following reference appears in the supplemental information: Schmitzer-Torbert et al. (2005).

## Supplemental information

Document S1. Figures S1–S8 and Methods S1

Document S2. Article plus supplemental information
