# Supplementary material for: Grid-cell modules remain coordinated when neural activity is dissociated from external sensory cues
Source: Neuron. 2022 Jun 1;110(11):1843–1856.e6. doi: 10.1016/j.neuron.2022.03.011 (PMC9235855; doi:10.1016/j.neuron.2022.03.011)
Supplement: Document S1. Figures S1–S8 and Methods S1 [file mmc1.pdf]

Neuron, Volume *110*

Supplemental information

Grid-cell modules remain coordinated when neural activity is dissociated from external sensory cues

Torgeir Waaga, Haggai Agmon, Valentin A. Normand, Anne Nagelhus, Richard J. Gardner, May-Britt Moser, Edvard I. Moser, and Yoram Burak

Supplemental Information

Grid-cell modules remain coordinated when neural activity is dissociated from external sensory cues

Torgeir Waaga[†,1], Haggai Agmon[†,2,4], Valentin A. Normand[1], Anne Nagelhus[1], Richard J. Gardner[1], May-Britt Moser[1,5], Edvard I. Moser[1,4,5] & Yoram Burak[2,3,4,5,6]

[1] Kavli Institute for Systems Neuroscience and Centre for Neural Computation, Norwegian University of Science and Technology, Trondheim, Norway.

[2] Edmond and Lily Safra Center for Brain Sciences, The Hebrew University of Jerusalem, Jerusalem, Israel.

[3] Racah Institute of Physics, The Hebrew University of Jerusalem, Jerusalem, Israel.

[4] Corresponding author (haggai.agmon@mail.huji.ac.il, edvard.moser@ntnu.no, yoram.burak@elsc.huji.ac.il).

[5] Senior author.

[6] Lead contact.

[†] These authors contributed equally.

Supplementary Figures:

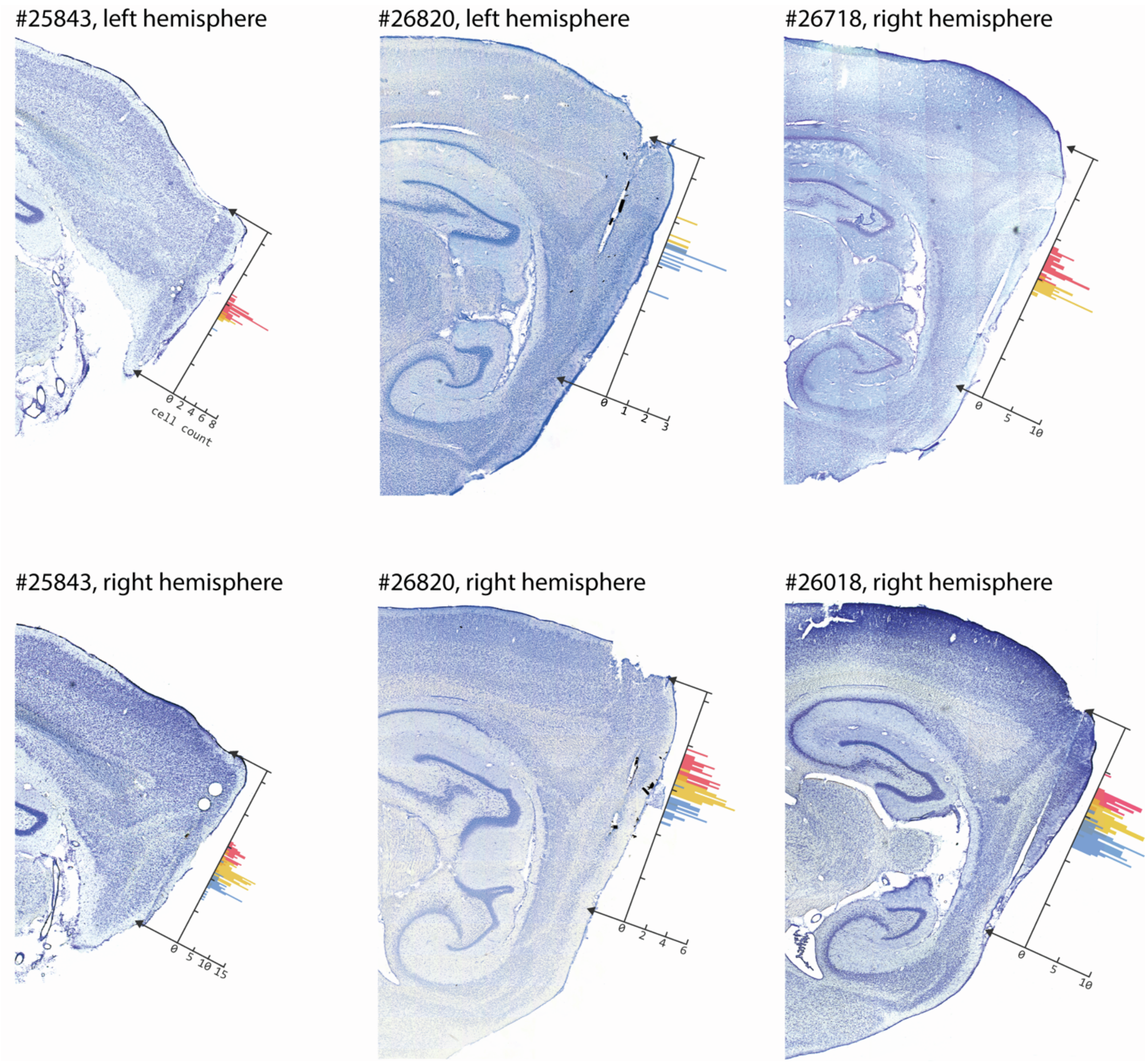

Figure S1. Histology and estimated recording sites. Related to Figure 1.
One cresyl violet-stained sagittal section is shown for each rat's neuropixels probe, showing the probe track left in the brain tissue. Estimated entering sites in the brain as well as probe tip locations are marked with arrows. The histogram shows the grid cell count across dorso-ventral recording depths from different modules (color coded). The distance between two adjacent ticks along the probe shank axis corresponds to 1 mm.

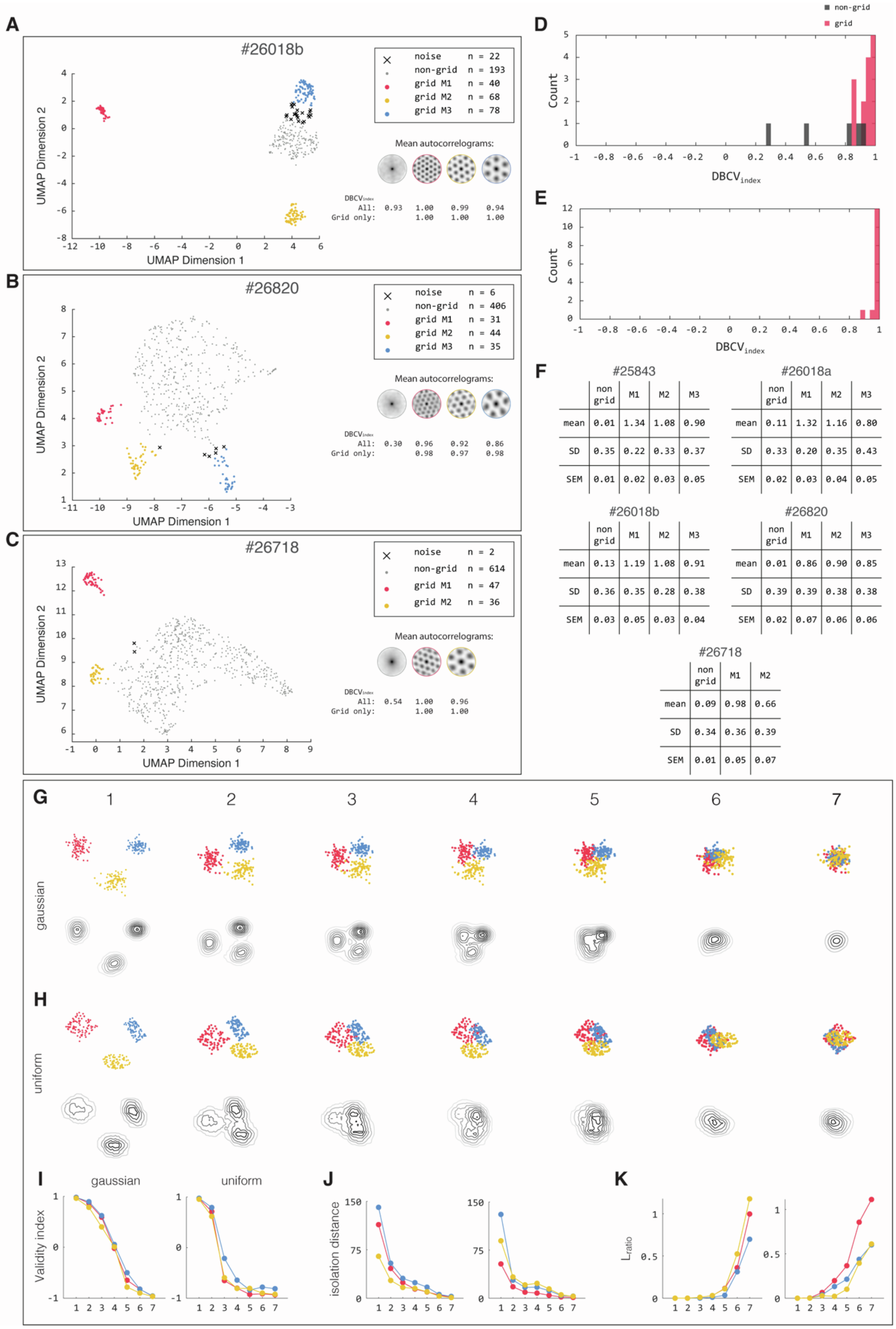
A
#26018b
noise n = 22
non-grid n = 193
grid M1 n = 40
grid M2 n = 68
grid M3 n = 78
Mean autocorrelograms:
DBCVindex
All: 0.93 1.00 0.99 0.94
Grid only: 1.00 1.00 1.00
UMAP Dimension 1
UMAP Dimension 2
B
#26820
noise n = 6
non-grid n = 406
grid M1 n = 31
grid M2 n = 44
grid M3 n = 35
Mean autocorrelograms:
DBCVindex
All: 0.30 0.96 0.92 0.86
Grid only: 0.98 0.97 0.98
C
#26718
noise n = 2
non-grid n = 614
grid M1 n = 47
grid M2 n = 36
Mean autocorrelograms:
DBCVindex
All: 0.54 1.00 0.96
Grid only: 1.00 1.00
D
non-grid
grid
Count
DBCVindex
E
Count
DBCVindex
F
#25843
non grid M1 M2 M3
mean 0.01 1.34 1.08 0.90
SD 0.35 0.22 0.33 0.37
SEM 0.01 0.02 0.03 0.05
#26018a
non grid M1 M2 M3
mean 0.11 1.32 1.16 0.80
SD 0.33 0.20 0.35 0.43
SEM 0.02 0.03 0.04 0.05
#26018b
non grid M1 M2 M3
mean 0.13 1.19 1.08 0.91
SD 0.36 0.35 0.28 0.38
SEM 0.03 0.05 0.03 0.04
#26820
non grid M1 M2 M3
mean 0.01 0.86 0.90 0.85
SD 0.39 0.39 0.38 0.38
SEM 0.02 0.07 0.06 0.06
#26718
non grid M1 M2
mean 0.09 0.98 0.66
SD 0.34 0.36 0.39
SEM 0.01 0.05 0.07
G
1 2 3 4 5 6 7
gaussian
H
uniform
I
gaussian
uniform
Validity index
J
isolation distance
K
Lratio

Figure S2. Grid cell classification by UMAP-DBSCAN. Related to Figure 1.
A-C, Same as Figure 1E-F but for three additional recording sessions. **D,** Distribution of validity index with non-grid cluster included for all rats and recordings. **E,** Distribution of validity index with non-grid cluster excluded for all rats and recordings. **F,** Tables of gridness scores (mean, SD, and SEM) for clusters corresponding to grid modules as well as non-grid clusters (UMAP-DBSCAN clusters for all recording sessions). **G-K,** Clustering quality validation: examples of DBCV index on varying clustering quality. Synthetic data points in clusters with varying degree of separation, either random two-dimensional gaussian distributions (G) or random uniform distributions with different shapes (H). All plots have the same scale. **G-H,** Top: synthetic data scatterplot with color coded cluster assignment. Bottom: contour plots illustrate the density. **I,** Density based clustering validity (DBCV) index of the clusters in (G, left) and (H, right), with color corresponding to cluster. Well separated clusters have a DBCV index above zero. **J-K,** We include the isolation distance and L-Ratio measures (Schmitzer-Torbert et al., 2005) commonly used for comparison of clusters of tetrode-recorded spikes from the hippocampus. The two measures were developed for measuring clustering quality for spike sorting tetrode data and assume the clusters form a gaussian distribution in feature space; they might not be ideal for quantifying density-based clustering results in UMAP space.

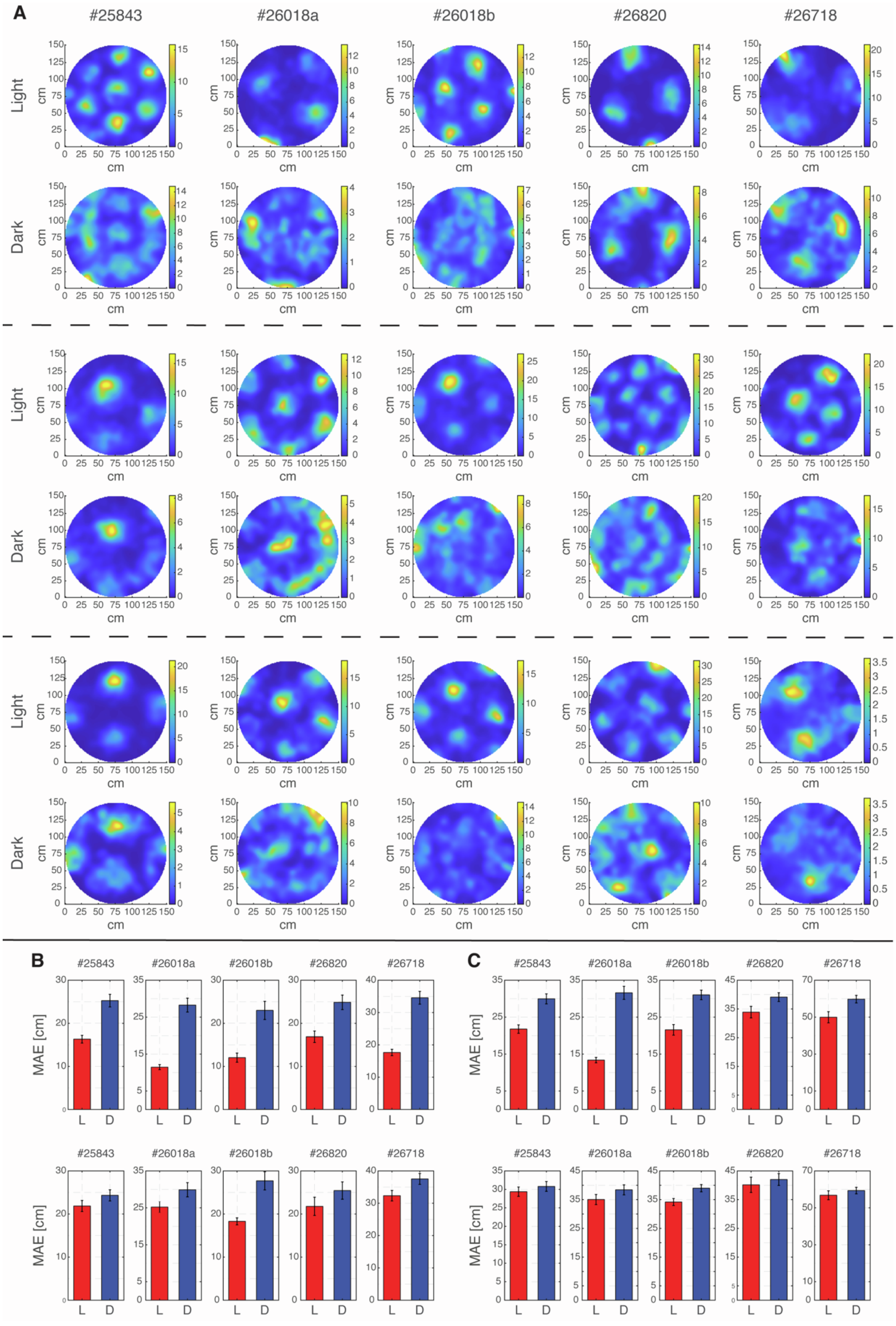
A
#25843
#26018a
#26018b
#26820
#26718
Light
Dark
cm
B
C
MAE [cm]
L
D

Figure S3. Additional individual grid cell rate maps examples from light and dark and unimproved decoding performance even when using dark generated rate maps. Related to Figure 2.

A, Same as Figure 2A, but for another three examples from each recording session. **B,** Top: Mean Absolute Error (MAE) of the Markov decoder applied on the light and dark recorded spiking activity for all cells from single recording sessions and when rate maps were constructed only from one half of the light data (and used to decode the other half of data). Bottom: same as top, but when rate maps were constructed only from one half of the dark data (and used to decode the other half of data). As expected, using the dark-generated rate maps leads to an increase of the MAE both in light and dark conditions, relative to decoding using light-generated rate maps. Importantly, the MAE of decoded position in the dark recording sessions is still larger than in light sessions, even when dark-generated maps are used. Error bars are ±SEM. **C,** Same as (B) but for the kernel decoder.

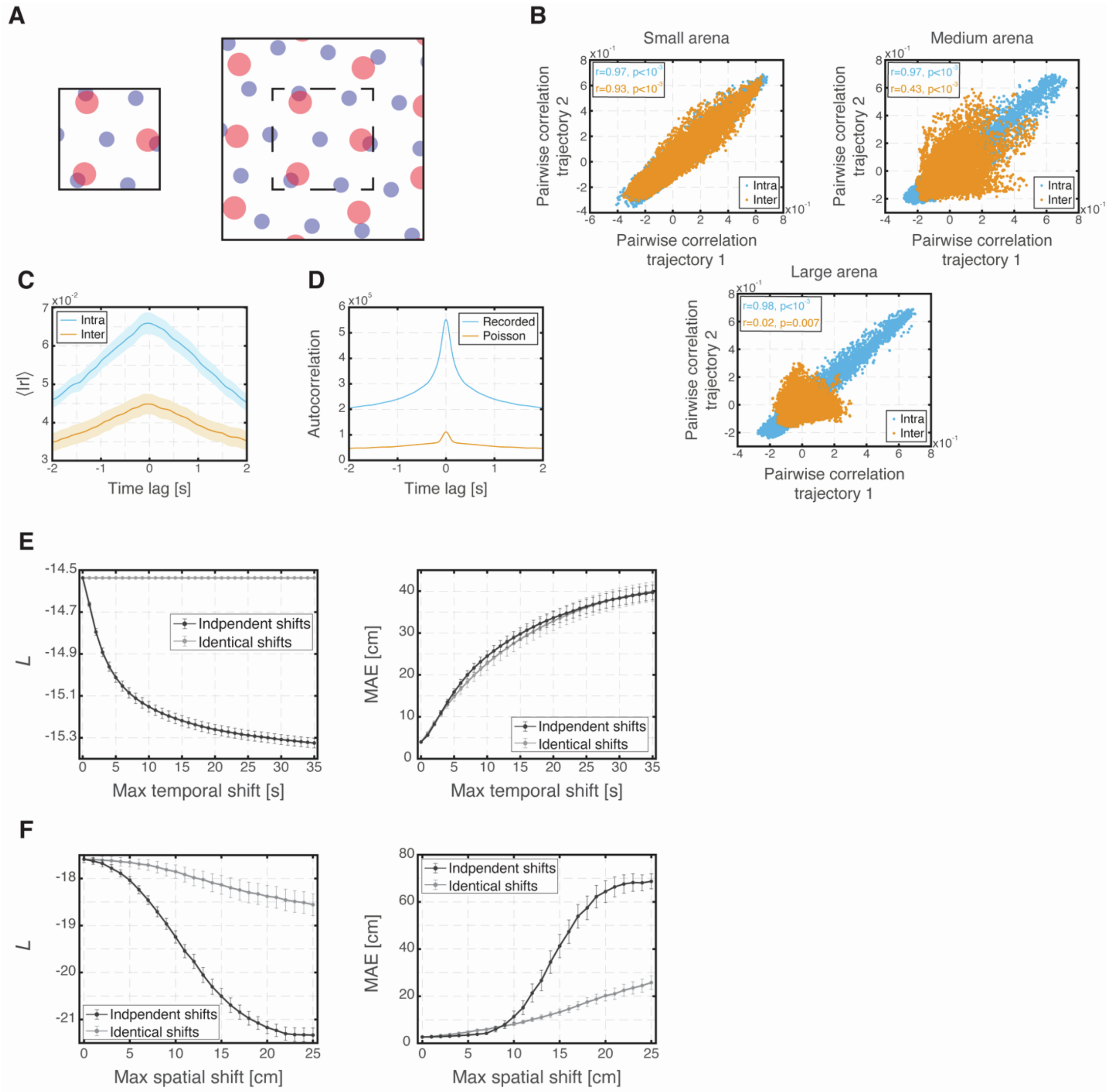

Figure S4. Additional pairwise correlation analyses and controls of the likelihood approach using simulated data. Related to Figure 3 and Figure 4.

A-B, *Inter-module pairwise correlations diminish with increased environment Size.* **A,** Schematic illustration showing spatial tuning curves of two inter-module grid cells (blue and red). The mean correlation between the tuning curves is higher when the environment is small (left) than when it is large (right). In larger environments, the spike rate correlations are expected to be more narrowly distributed around Zero. **B,** Pairwise correlations of all possible intra- (cyan) and inter- (orange) module pairs from two simulated trajectories in small (radius=30 cm), medium (radius=60 cm) and large (radius=150 cm) circular arenas. Correlation coefficients and p-values are specified in the insets. As expected, inter-module spike rate correlations became narrowly distributed around zero with an increase in the size of the environment, whereas intra-module spike rate correlations remained unaffected. Simulated grid cells share the spacings and module allocation as recorded in session #26018a and emit Poisson spikes which

are determined by their idealized tuning curves (*Methods*). **C-D,** *Sources of variability other than spatial selectivity, contribute to the spike rate correlations.* **C,** Absolute cross-correlation (Pearson coefficient) of inter- and intra-module spiking activities, averaged over cell pairs, obtained from Poisson spike trains which were simulated in response to the light trajectory from recording session #26018a. Shaded error bars are ±SEM. Note that the spatial selectivity on its own is predicted to generate cross correlations that decay slowly in time, but there are notable differences when compared with the actual data. First, the temporal structure of the decay predicted by the Poisson model differs from the structure observed in the data (Figure 3B). Second, the Poisson model does not fully explain the magnitude of cross correlations at the zero-lag (Figure 3C). **D,** Temporal autocorrelation of the population activity from the light trial in recording session #26018a (blue trace). The population activity signal was evaluated as a sum over all cells of their temporally smoothed spiking activity. The orange trace shows the predicted temporal autocorrelation obtained from Poisson spike trains which were simulated in response to the same trajectory. As expected, the autocorrelation predicted by the Poisson model is weak, because the summed firing rate of all the neurons is only weakly modulated by the position of the animal, and in the Poisson model this is the only determinant of the cell activity. The difference between the traces suggests that network-wide fluctuations in the activity of cells, of non-spatial origin, contribute to the correlations in addition to the spatial selectivity, but note that much of the zero-lag correlations is well predicted by the Poisson model (Figure 3C). **E,** *Likelihood slightly decreases under identical spatial shifts due to boundary conditions.* In order to demonstrate that the slight decrease in the likelihood when using identical spatial shifts is due to boundary conditions, temporal shifts were applied to the same data as in Figure 4B-C. Independent temporal shifts were applied by shifting the timing of spike trains of all neurons that belong to the same module, and identical temporal shifts were applied in a similar fashion but identically for all neurons regardless of the module they belong to. Rate maps were unaffected during this procedure thus their boundaries were not trimmed as in the spatial shift procedure (see also Figure S6B for a similar result using rotational shifts). **Left:** Likelihood of simulated Poisson spikes using measured rate maps and recorded light trajectory from session #26018b for varying magnitudes of temporal shifts. When shifts are applied independently for each module, the likelihood decreases significantly while remaining precisely fixed when temporal shifts are identical. Error bars are ±SEM. **Right:** The Mean Absolute Error (MAE) increases significantly both for independent and for identical temporal shifts as their magnitude increases. Error bars are ±SEM. **F,** *Applying spatial shifts during spike generation is equivalent to spatial shift implementation during decoding.* Similar analysis as in Figure 4. Instead of applying spatial shifts to the rate maps in the decoding process, Poisson spike trains were generated using idealized grid cell tuning curves (*Methods*) under varying magnitudes of spatial shifts that were applied directly to the position encoded by each module. This represents more closely the type of shifts that may occur in the recorded data. Note, however, that it is impossible to apply spatial shifts directly to the recorded spike trains. The goal of these simulations is to demonstrate that the two procedures yield similar outcomes. **Left:** Likelihood of simulated Poisson spikes using idealized grid cell tuning curves and recorded light trajectory from recording session #26018b, evaluated versus varying magnitudes of spatial shifts which were applied to the rate maps during the simulated spike generation. Compare with Figure 4B (values of the likelihood differ because of the idealized tuning curves used here, vs. the measured rate maps used in Figure 4B-C). Error bars are ±SEM. **Right:** The corresponding MAE of the decoder. The MAE increases significantly both for independent and for identical spatial shifts as their magnitude increases. It increases more rapidly for independent shifts due to global decoding errors. This effect is stronger than in Figure 4C due to the use of idealized tuning curves, which allows to complete the tuning curves outside the extent of the arena after applying the shift, whereas in Figure 4C rate maps were completed to zero in regions that were outside of the arena boundaries before the shift (*Methods*). Error bars are ±SEM.

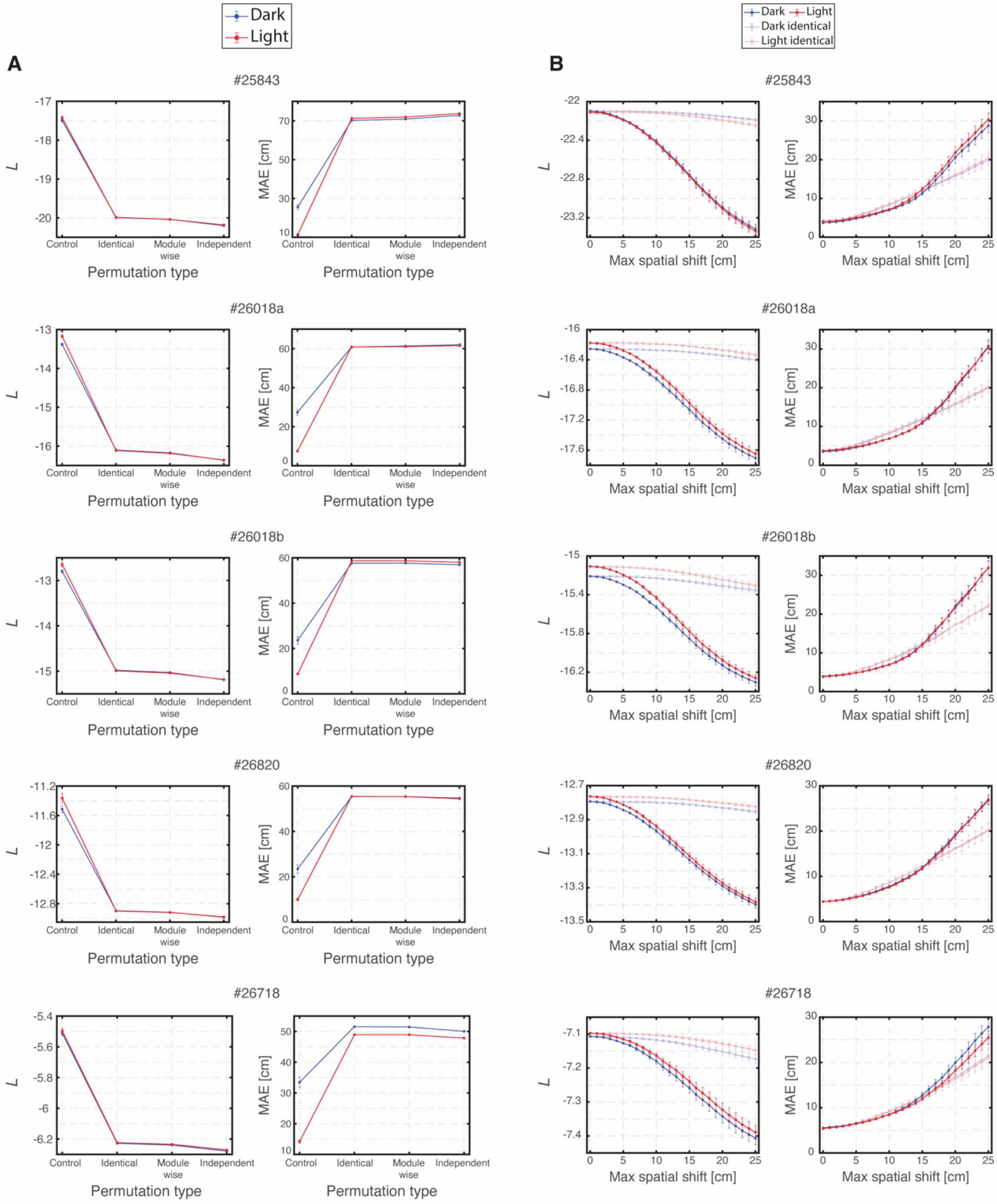

Figure S5. Additional controls of the likelihood approach using recorded data. Related to Figure 5.
A, *The temporal structure of simultaneously recorded spike trains, and not simply their mean firing rate, is necessary to account for the likelihood and MAE results.*

Three types of permutations, which preserve the mean firing rates, were applied to the simultaneous recorded spike trains. ‘Identical’ describes a permutation type in which the exact permutation was applied to all neurons, ‘Module-wise’ describes a permutation type in which identical permutations were applied but only to neurons that belong to the same module, and ‘Independent’ describes a permutation type in which an independent permutation was applied to each neuron. Likelihood of simultaneous recorded spike trains (left column) and corresponding Mean Absolute Error (MAE, right column) of dark and light trials from all recording sessions are shown for the different permutation types. Error bars are ±SEM.
B, *Motion statistics differences between the light and dark trajectories are not expected to substantially affect the likelihood.*
Simulated Poisson spikes were generated for trajectories taken either from the light or dark trials. Rate maps and all other parameters were identical in the two sets of simulations. Rate adjusted likelihood (left column) and Mean Absolute Error (MAE, right column) using the light and dark trajectories for all recording sessions are shown for varying magnitudes of independent module-wise spatial shifts. The differences between the zero-shift light and dark likelihoods are smaller than their corresponding differences shown in Figure 5B-C and Figure S6A, indicating that the difference in motion statistics between light and dark trajectories does not substantially affect the likelihood. As expected, the MAE is nearly identical for both light and dark trajectories. Identical spatial shifts in all modules are superimposed using faded colors for reference. Error bars are ±SEM.

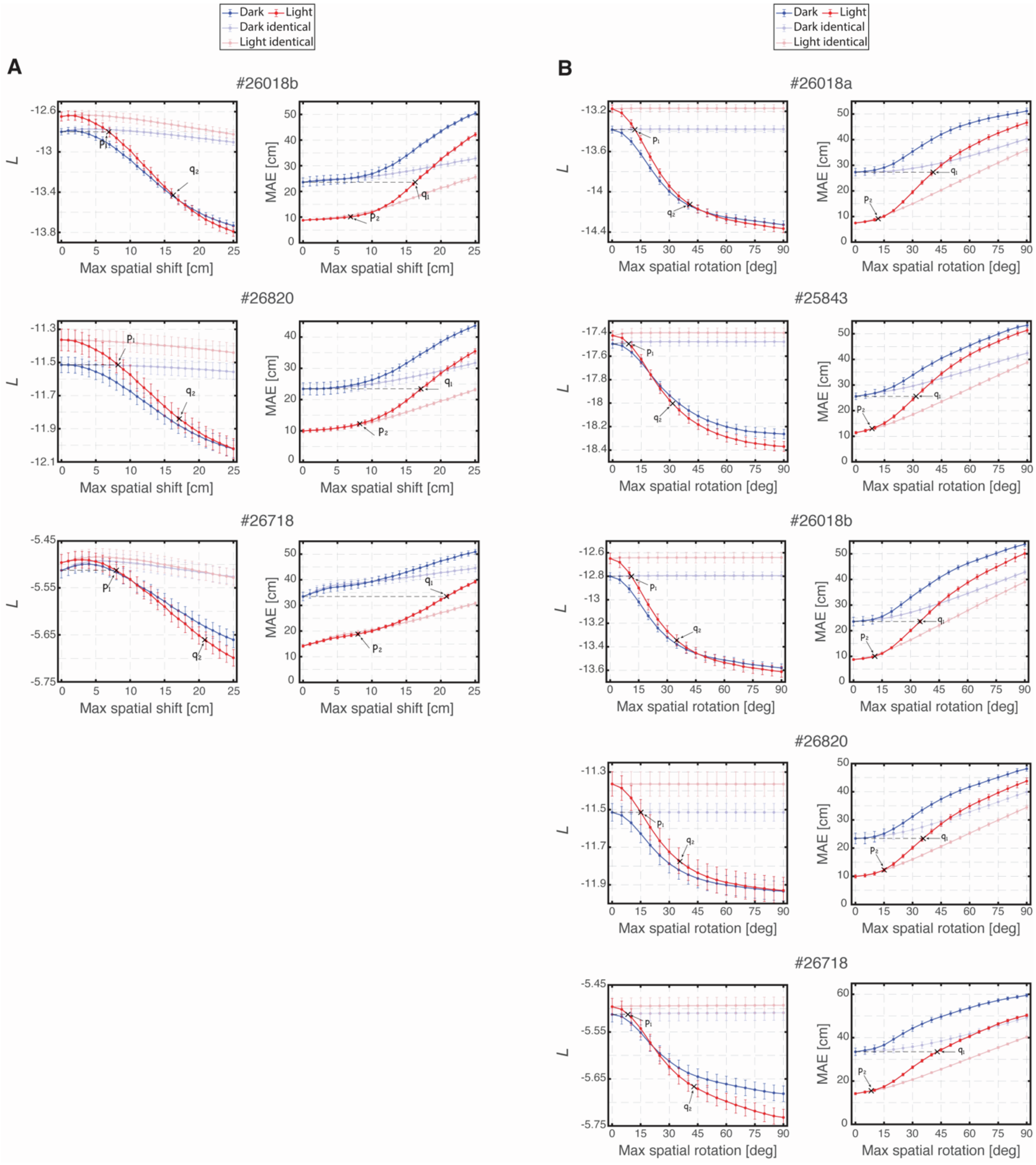

Figure S6. Additional recorded data analyses using the likelihood-based approach. Related to Figure 5.
A, Same as Figure 5C, for three additional recording sessions. The Mean Absolute Errors (MAEs) under the null hypotheses (*Methods*) for the light and dark trials are ~71 cm and ~70 cm correspondingly in recording session #26018b, ~66 cm and ~66 cm respectively in recording session #26820, and ~70 cm and ~72 cm respectively in recording session #26718. Error bars are ±SEM.
B, *Analysis of simultaneously recorded spike trains using rotational shifts.*

Same as Figure 5 but with application of rotational shifts (*Methods*) instead of spatial shifts. Likelihood of simultaneously recorded spike trains (left column) and corresponding Mean Absolute Error (MAE, right column) of dark and light trials from all recording sessions are shown for varying magnitudes of independent module-wise rotational shifts. Recording session #26018a results are explained as an example (top panels): applying a maximal spatial rotation of 12 degrees in the #26018a light recording achieves the same likelihood as that of the dark recording with zero spatial rotation (point p_1, top left panel), but generates only a slight increase of less than 2 cm in the corresponding MAE of the light recording relative to its zero spatial rotation value (point p_2, top right panel). Conversely, applying a maximal spatial rotation of 41 degrees in the #26018a light recording achieves the same MAE as that of the dark recording with zero spatial rotation (point q_1, top right panel), but generates a dramatic decrease in the likelihood (point q_2, top left panel). The difference in the likelihood between point q_2 and the zero spatial rotation point of the dark recording is much larger than the difference between the likelihood values of light and dark zero spatial rotation points. Identical rotational shifts in all modules are superimposed using faded colors. Under identical rotational shifts the dark and light likelihoods are completely unaffected (left) while the corresponding MAE increases significantly in both cases (right). Error bars are ±SEM.

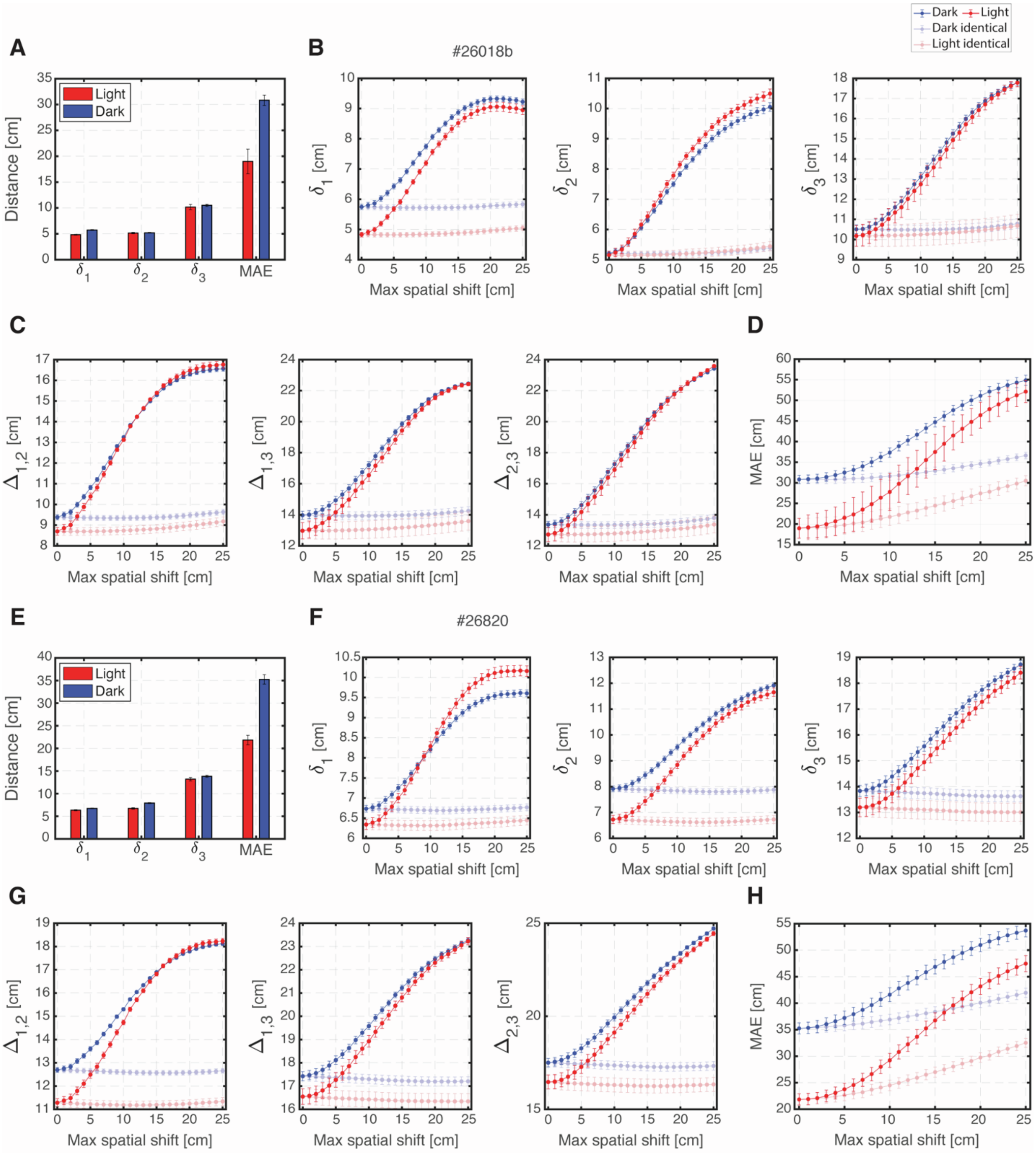

Figure S7. Results for additional datasets using the uni-module decoding approach. Related to Figure 6. A-D and **E-H,** Same as Figure 6B-E for two additional recording sessions (#26018b and #26820) in which recordings were obtained from three modules. Note that even though recordings from three modules were available in these datasets, the numbers of simultaneously recorded grid cells were relatively small (especially in recording session #26820; Table 1), leading to inaccurate decoding. The δ_is under the null hypotheses (*Methods*) for the light and dark trials are ~[13.7,18.2,25.7] cm and ~[13.8,18.2,25.6] cm respectively in (A), and ~[14.4,18.2,27.3] cm and ~[14.4,18.2,27.3] cm respectively in (E). Error bars are ±SEM.

Methods S1

Analytic derivations of the likelihood-based approach. Related to Figure 4, Figure 5, and STAR Methods.

a. Likelihood of simultaneously recorded spike trains

In this subsection we mathematically derive the likelihood of simultaneously recorded spike trains, independently from the animal's true position. To address the inter-module coordination question, we sought to derive a measurement which can quantify the coherence of the simultaneously recorded spike trains even if the represented position in the brain is dissociated from the animal's true position.

Denote by $\boldsymbol{X}_t \equiv \{\vec{x}_0, \vec{x}_1, \dots, \vec{x}_t\}$ a particular realizable two-dimensional trajectory up to time t, and by $p(\boldsymbol{X}_t)$ the probability for the trajectory $\boldsymbol{X}_t$ to be realized. Since the prior on the trajectory is Markovian, $p(\boldsymbol{X}_t)$ satisfies

$$p(\boldsymbol{X}_t) = p(\vec{x}_t|\vec{x}_{t-1}) \cdot p(\vec{x}_{t-1}\,|\vec{x}_{t-2}) \cdots p(\vec{x}_1|\vec{x}_0) \cdot p(\vec{x}_0) \tag{S1}$$

where $p(\vec{x}_0)$ is the probability of the initial position.

The probability to observe the simultaneous recorded spike trains, averaged over all possible trajectories up to time t, is written as

$$p(\boldsymbol{S}_t) = \sum_{\boldsymbol{X}_t} p(\boldsymbol{X}_t) \cdot p(\boldsymbol{S}_t|\boldsymbol{X}_t) \tag{S2}$$

where the sum is over all possible trajectories, weighted by their corresponding priors $p(\boldsymbol{X}_t)$.

Extending Eq. S2 for the consecutive time step $t+1$,

$$p(\boldsymbol{S}_{t+1}) = \sum_{\boldsymbol{X}_{t+1}} p(\boldsymbol{X}_{t+1}) \cdot p(\boldsymbol{S}_{t+1}|\boldsymbol{X}_{t+1}) \tag{S3}$$

Due to the Markov properties $p(\boldsymbol{X}_{t+1}) = p(\vec{x}_{t+1}|\vec{x}_t) \cdot p(\boldsymbol{X}_t)$, and due to the relationship $p(\boldsymbol{S}_{t+1}|\boldsymbol{X}_{t+1}) = p(\boldsymbol{S}_t|\boldsymbol{X}_t) \cdot p(s_{t+1}|\vec{x}_{t+1})$, Eq.S3 can be written as follows:

$$p(\boldsymbol{S}_{t+1}) = \sum_{\boldsymbol{X}_t} \sum_{x_{t+1}} p(\boldsymbol{X}_t) \cdot p(\boldsymbol{S}_t|\boldsymbol{X}_t) \cdot p(s_{t+1}|\vec{x}_{t+1}) \cdot p(\vec{x}_{t+1}|\vec{x}_t) \tag{S4}$$

Using Bayes law and rearranging yields

$$p(\boldsymbol{S}_{t+1}) = p(\boldsymbol{S}_t) \cdot \sum_{\boldsymbol{X}_t} \sum_{\vec{x}_{t+1}} p(\boldsymbol{X}_t|\boldsymbol{S}_t) \cdot p(s_{t+1}|\vec{x}_{t+1}) \cdot p(\vec{x}_{t+1}|\vec{x}_t) \tag{S5}$$

Re-writing the Markov decoder from the *Methods* (Eq. 2) using this notation,

(S6)

$$p(x_{t+1}|\boldsymbol{S}_{t+1}) = \frac{1}{Z_{t+1}} \cdot \sum_{\boldsymbol{X}_t} [p(\boldsymbol{X}_t|\boldsymbol{S}_t) \cdot p(\vec{x}_{t+1}|\vec{x}_t)] \cdot p(s_{t+1}|\vec{x}_{t+1})$$

where the normalization factor Z_{t+1} satisfies the demand

(S7)

$$\sum_{\vec{x}_{t+1}} p(\vec{x}_{t+1}|\boldsymbol{S}_{t+1}) \stackrel{!}{=} 1$$

Summing over $\sum_{\vec{x}_{t+1}}$ on both sides of Eq. S6 and rearranging we obtain

(S8)

$$Z_{t+1} = \sum_{\boldsymbol{X}_t} \sum_{\vec{x}_{t+1}} p(\boldsymbol{X}_t|\boldsymbol{S}_t) \cdot p(\vec{x}_{t+1}|\vec{x}_t) \cdot p(s_{t+1}|\vec{x}_{t+1})$$

which is identical to the expression in Eq. S5. Thus, and in recursion,

(S9)

$$p(\boldsymbol{S}_{t+1}) = p(\boldsymbol{S}_t) \cdot Z_{t+1} = p(\boldsymbol{S}_{t-1}) \cdot Z_t Z_{t+1} = \cdots = p(\boldsymbol{S}_{t_0}) \cdot \prod_{i=1}^{t+1} Z_i$$

Finally, applying natural logarithm to Eq. S9 while neglecting the border term $p(\boldsymbol{S}_{t_0})$, which is justified once sufficient iterations have been made, yields

(S10)

$$\log[p(\boldsymbol{S}_t)] = \sum_{i=1}^{t} \log(Z_i)$$

Qualitatively, the likelihood is accrued in a linear fashion over time, indicating that $\log(Z)$ was drawn from an approximately stationary distribution within each recording session (Figure S8A). Therefore, to compare results between light and dark trials, we define the likelihood of simultaneous recorded spike trains as the mean of $\log(Z)$ over time, which can be thought as the amount of likelihood per time unit,

(S11)

$$L \equiv \langle \log(Z) \rangle_t$$

b. *Rate-adjusted likelihood*

In this subsection we elucidate analytically why the posterior likelihood depends on the mean firing rate, and counter-intuitively decreases with increased spiking activity.

Denote by ξ a series of time binned spike trains which can take only $\{0,1\}$ values in each time bin Δt. Under the assumption of Poisson firing and in the limit of small Δt, the posterior likelihood is written as

(S12)

$$\xi_i \cdot \log[\lambda(x_i)\Delta t] + (1 - \xi_i) \log[1 - \lambda(x_i)\Delta t] \cong -\int \lambda(x_t)\mathrm{d}t + \sum_{i=1}^{T} \xi_i \cdot [\log[\lambda(x_i)\Delta t] + \lambda(x_i)\Delta t]$$

Since $0 < \lambda(x_i)\Delta t \ll 1, \forall\, i$, we conclude (counter intuitively) that the addition of each spike can only decrease the posterior likelihood. This can be interpreted as arising from the fact that in the limit of small

Δt, the update of the likelihood with the addition of each spike is proportional to the probability that this particular spike will fall exactly within the relevant time bin.

Thus, to faithfully compare likelihoods between light and dark trials, it was essential to take into account mean firing rates modifications. The underlying assumption in the likelihood approach is that emitted spikes in darkness were generated from the same tuning curves as observed in light up to a multiplicative scaling factor. We down-sampled spike trains by randomly omitting spikes until the mean firing rates matched between the two trials, thus producing rate-adjusted spike trains. To demonstrate that the likelihood indeed depends on the mean firing rate and to justify that such random down sampling is appropriate, we varied a multiplicative factor which controlled the firing rate which accounts for the generation of simulated Poisson spike trains. As expected, the evaluated likelihood decreased as the mean firing rate increased but was completely unaffected if spike trains were subsequently down-sampled randomly using the corresponding multiplicative factor (Figure S8B).

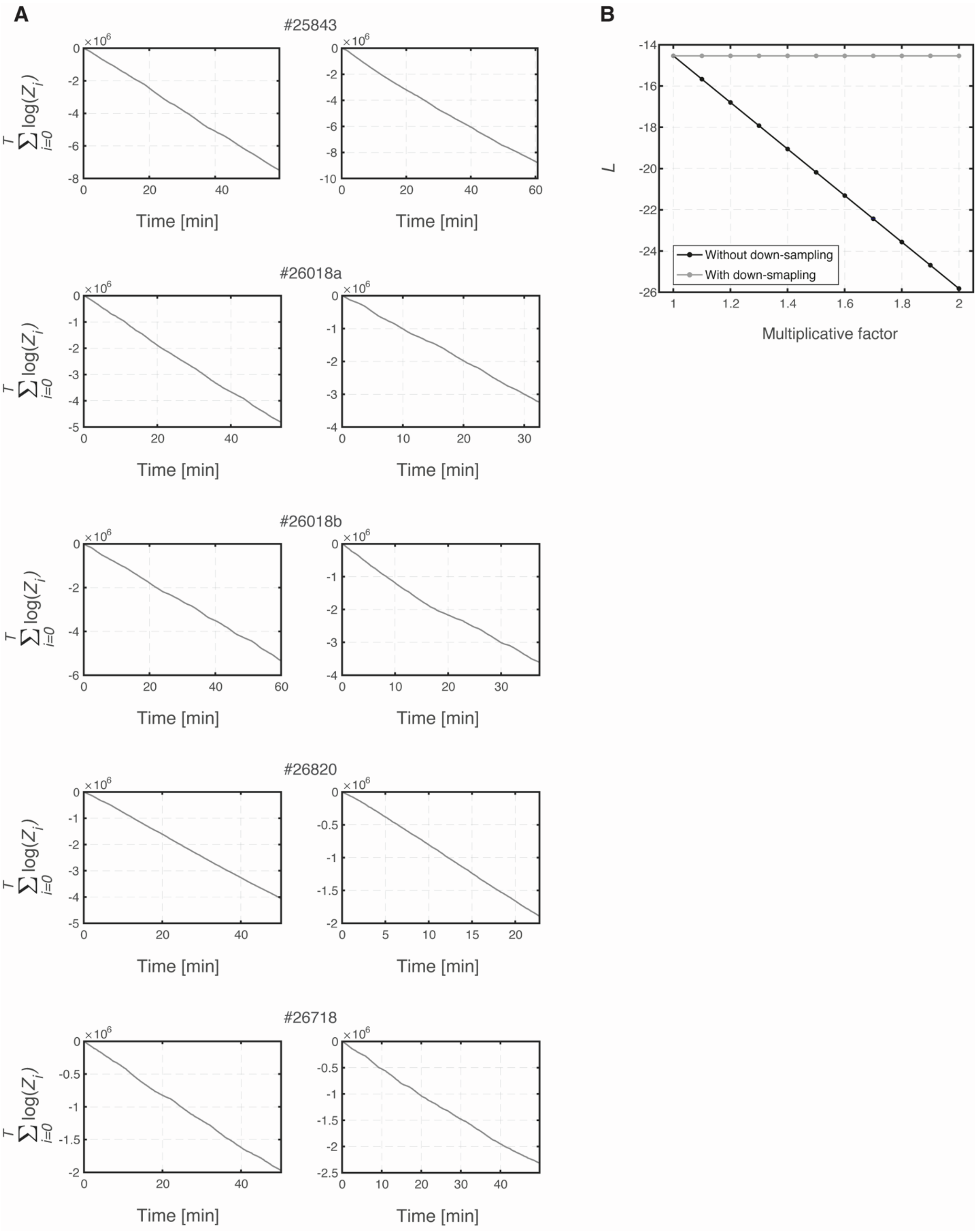
A
#25843
#26018a
#26018b
#26820
#26718
Time [min]
×10^6
B
Without down-sampling
With down-smapling
Multiplicative factor
L

Figure S8. Log likelihood versus time and the likelihood dependence on the mean firing rate. Related to STAR Methods and Methods S1.

A, Cumulative sum over time of $\log[p(\boldsymbol{S}_t)]$ in dark (left column) and in light (right column) trials from all recording sessions. Linear dependence (R^2>0.99 for both light and dark and across all sessions, fit not shown) is evident, indicating a steady accumulation of the log likelihood per time bin. **B,** The likelihood L was evaluated when using rate maps and light trajectory from recording session #26018b but with simulated Poisson spikes. Rate maps were first scaled by a multiplicative factor (x axis) thus leading to a constant decrease in the likelihood as the firing rate increases (black trace, as expected by Eq. S12). However, when the spike trains that were generated using the scaled rate maps were randomly down-sampled to match their original mean firing rate, the original likelihood value was precisely restored (gray trace).
