# Supplementary material for: Grid-cell modules remain coordinated when neural activity is dissociated from external sensory cues
Source: Neuron. 2022 Jun 1;110(11):1843–1856.e6. doi: 10.1016/j.neuron.2022.03.011 (PMC9235855; doi:10.1016/j.neuron.2022.03.011)
Supplement: Document S2. Article plus supplemental information [file mmc6.pdf]

Neuron

Article

Grid-cell modules remain coordinated when neural activity is dissociated from external sensory cues

Highlights

- Hundreds of grid cells were recorded simultaneously from multiple grid modules
- Coordination between grid modules was assessed in rats that foraged in darkness
- Coordination persists despite relative drift of the represented versus true position
- This suggests that internal network mechanisms maintain inter-module coordination

Authors

Torgeir Waaga, Haggai Agmon, Valentin A. Normand, ..., May-Britt Moser, Edvard I. Moser, Yoram Burak

Correspondence

haggai.agmon@mail.huji.ac.il (H.A.), edvard.moser@ntnu.no (E.I.M.), yoram.burak@elsc.huji.ac.il (Y.B.)

In brief

The position of an animal in its environment is represented in the entorhinal cortex by distinct modules of grid cells. Waaga, Agmon et al. show that in darkness, the internal representation of position drifts relative to the true position of the animal; yet, it remains tightly coordinated across the distinct modules.

Waaga et al., 2022, Neuron *110*, 1843–1856
June 1, 2022 © 2022 The Author(s). Published by Elsevier Inc.
https://doi.org/10.1016/j.neuron.2022.03.011

CellPress

Neuron

CellPress
OPEN ACCESS

Article

Grid-cell modules remain coordinated when neural activity is dissociated from external sensory cues

Torgeir Waaga,[1,4] Haggai Agmon,[2,4,*] Valentin A. Normand,[1] Anne Nagelhus,[1] Richard J. Gardner,[1] May-Britt Moser,[1,5] Edvard I. Moser,[1,5,*] and Yoram Burak[2,3,5,6,*]

[1]Kavli Institute for Systems Neuroscience and Centre for Neural Computation, Norwegian University of Science and Technology, Trondheim, Norway
[2]Edmond and Lily Safra Center for Brain Sciences, The Hebrew University of Jerusalem, Jerusalem, Israel
[3]Racah Institute of Physics, The Hebrew University of Jerusalem, Jerusalem, Israel
[4]These authors contributed equally
[5]Senior author
[6]Lead contact
*Correspondence: haggai.agmon@mail.huji.ac.il (H.A.), edvard.moser@ntnu.no (E.I.M.), yoram.burak@elsc.huji.ac.il (Y.B.)
https://doi.org/10.1016/j.neuron.2022.03.011

SUMMARY

The representation of an animal's position in the medial entorhinal cortex (MEC) is distributed across several modules of grid cells, each characterized by a distinct spatial scale. The population activity within each module is tightly coordinated and preserved across environments and behavioral states. Little is known, however, about the coordination of activity patterns across modules. We analyzed the joint activity patterns of hundreds of grid cells simultaneously recorded in animals that were foraging either in the light, when sensory cues could stabilize the representation, or in darkness, when such stabilization was disrupted. We found that the states of different modules are tightly coordinated, even in darkness, when the internal representation of position within the MEC deviates substantially from the true position of the animal. These findings suggest that internal brain mechanisms dynamically coordinate the representation of position in different modules, ensuring that they jointly encode a coherent and smooth trajectory.

INTRODUCTION

Recently, techniques that enable simultaneous recording of activity in dozens to hundreds of neurons (Ghosh et al., 2011; Jun et al., 2017; Steinmetz et al., 2021; Zong et al., 2017) have enabled a shift from the measurement of single-cell activity in relationship to external correlates to the investigation of the joint population activity patterns in large neural ensembles. This change of perspective has led to various attempts to characterize neural activity patterns as residing within restricted, low-dimensional spaces using linear (Gallego et al., 2018; Mazor and Laurent, 2005; Stringer et al., 2019) or non-linear (Chaudhuri et al., 2019; Gardner et al., 2022; Rubin et al., 2019; Rybakken et al., 2019) dimensionality reduction techniques. One of the most striking outcomes of these attempts has emerged in neural circuits involved in the representation of an animal's position relative to the environment. In several such circuits in flies and mammals, neural activity patterns have been shown to robustly reside in low-dimensional non-linear manifolds, even when the neural activity is dissociated from external inputs to the network (Chaudhuri et al., 2019; Gardner et al., 2022; Kim et al., 2017; Rybakken et al., 2019; Seelig and Jayaraman, 2015). This finding opens up the possibility to decode the low-dimensional variable that is represented within these circuits and to examine how the brain utilizes such representations across multiple sub-circuits to implement computational functions.

Here, we examine the dynamics of grid cells in the medial entorhinal cortex (MEC). Grid cells exhibit multiple firing fields as a function of an animal's spatial location. The fields are arranged on a hexagonal lattice in open-field environments (Hafting et al., 2005). Within each individual animal, grid cells are allocated to discrete modules, each defined by a common grid spacing and angular orientation (Barry et al., 2007; Stensola et al., 2012). Jointly, the activity of grid cells across multiple modules implements a highly efficient population code for position (Burak, 2014; Fiete et al., 2008; Mathis et al., 2012; Sreenivasan and Fiete, 2011; Welinder et al., 2008).

The spatial tuning curves of individual grid cells indicate that grid-cell population activity within each module is confined to lie on a low-dimensional manifold with toroidal topology (Gardner et al., 2022). Accumulating evidence has suggested that this confinement is achieved through network mechanisms within the MEC, under diverse behavioral conditions and independently of inputs from other brain regions. Early evidence

This is an open access article under the CC BY license (http://creativecommons.org/licenses/by/4.0/).

came from observing the correlation structure of activity in pairs of cells: phase relationships between grid cells within a module are tightly preserved over time and across environments (Fyhn et al., 2007; Yoon et al., 2013). The phase relationships are maintained also during sleep (Gardner et al., 2019; Trettel et al., 2019) and under hippocampal inactivation, despite the absence of a grid-like spatial response pattern (Almog et al., 2019). Very recently, simultaneous recordings of spiking activity in dozens of cells provided direct evidence that neural activity patterns are closely confined to two-dimensional manifolds with toroidal topology, which are tightly preserved across environments and in sleep (Gardner et al., 2022). Thus, grid cells within a module encode together a two-dimensional quantity, which, in some conditions, could be dissociated from the true position of the animal.

All of the above findings are in agreement with predictions made by continuous attractor network (CAN) theory (Burak and Fiete, 2009; Fuhs and Touretzky, 2006; Guanella et al., 2007; McNaughton et al., 2006). According to this theory, grid cells within each module are recurrently connected and thus form a sub-network within the MEC. The recurrent synaptic connectivity within each module constrains the joint activity of cells to a restricted, but continuous, repertoire of possible coactivation patterns that is stable across behavioral states and conditions, even in the absence of sensory inputs.

Single modules alone, however, cannot represent a unique position of an animal within a typical environment. It is necessary to consider the coordination of activity across modules to assess how grid cells encode the brain's internal representation of position. The question of coordination becomes especially important under conditions in which sensory cues are poor or absent (Burak, 2014). Since population activity of an individual module lies on a two-dimensional manifold, the joint activity of M modules spans, at least in principle, a $2M$ dimensional space. However, during continuous motion in a given environment, and in the presence of salient sensory cues, the state of each module is faithfully mapped to the location of the animal in two-dimensional space. Hence, under continuous motion of the animal, the joint population activity patterns of multiple modules span a highly restricted two-dimensional subspace of the full $2M$ dimensional space. This raises the question as to whether the states of different modules are updated in a similarly coordinated manner when the states of individual modules are dissociated from the true position of the animal, e.g., in the absence of salient sensory cues.

The coordination of activity across grid-cell modules is highly consequential from the perspectives of neural coding and dynamics. The modular structure of the grid-cell code for position confers it with large representational capacity (Burak, 2014; Fiete et al., 2008; Mosheiff and Burak, 2019; Sreenivasan and Fiete, 2011; Welinder et al., 2008). However, within a given environment, the modularity of the grid-cell code poses a significant challenge for the neural circuitry that maintains the representation and updates it based on self-motion. Under conditions in which sensory inputs are absent or poor, the representation of position in individual grid-cell modules might drift relative to the actual position of the animal. If these drifts are not identical in different modules, they would rapidly lead to combinations of spatial phases that do not represent any position in the vicinity of the animal, resulting in abrupt shifts in the represented position. Thus, independent drifts lead to catastrophic errors when activities are read out from multiple grid-cell modules and would therefore be highly detrimental for the coding of position by grid-cell activity. The difficulty arising from occurrence of such catastrophic readout errors has been identified in early works on grid-cell coding (Fiete et al., 2008). Since then, two solutions have been proposed. In one solution (Agmon and Burak, 2020; Sreenivasan and Fiete, 2011; Welinder et al., 2008), the hippocampal network reads out the position represented by grid cells, and feedback projections from hippocampus to the MEC correct small, incompatible drifts accrued in each of the modules. A second solution (Kang and Balasubramanian, 2019; Mosheiff and Burak, 2019) involves synaptic connectivity between modules.

Empirically, however, very little is known about the relationship between population activity patterns of grid cells across distinct modules. Previous research has focused on coactivation patterns within modules for two reasons: first, simultaneously recorded cells using tetrodes often belonged to the same module. Second, coactivation patterns of inter- and intra-module grid-cell pairs are fundamentally different. Grid cells within a module maintain strict relationships in their activities that can be probed by analyzing the joint activity in pairs of simultaneously recorded cells. On the other hand, the activity of two cells that belong to different modules might be correlated or anti-correlated depending on the animal's position, even within a fairly small environment. Due to this lack of an expected rigid correlation (or anti-correlation), it is difficult to characterize inter-module coordination based on pair recording analysis. To identify higher-order dependencies across modules, it is necessary to decode activity from multiple cells within each module—requiring larger numbers of simultaneously recorded cells from multiple modules, which have only recently become available.

Here, using Neuropixels silicon probes (Jun et al., 2017; Steinmetz et al., 2021), we recorded the simultaneous activity of grid cells from multiple modules with dozens of units in each module. Rats were deprived of visual cues to test whether the internal representations of position in distinct modules remain coordinated even when dissociated from the animal's true position. By decoding the simultaneous grid-cell activity, we demonstrated that grid-cell modules retain, to a high extent, coordination even when the mapping between grid-cell activity and position deteriorates. These results indicate that network mechanisms within the brain coordinate the activity of different modules, independently of external sensory inputs.

RESULTS

We recorded spiking activity from rats foraging in a circular arena with a diameter of 150 cm under light and complete dark conditions (Figure 1A). The arena was cue-less except for a single vertical cue card at a fixed location along its circumference, which was visible in light and completely invisible in darkness, and tactilely inaccessible. The circular arena was rotationally symmetric, thus minimizing the information about absolute position coming from encounters with the walls (Hardcastle et al., 2015; Keinath et al., 2018). The arena was surrounded by a floor-to-ceiling blockout

CellPress
OPEN ACCESS

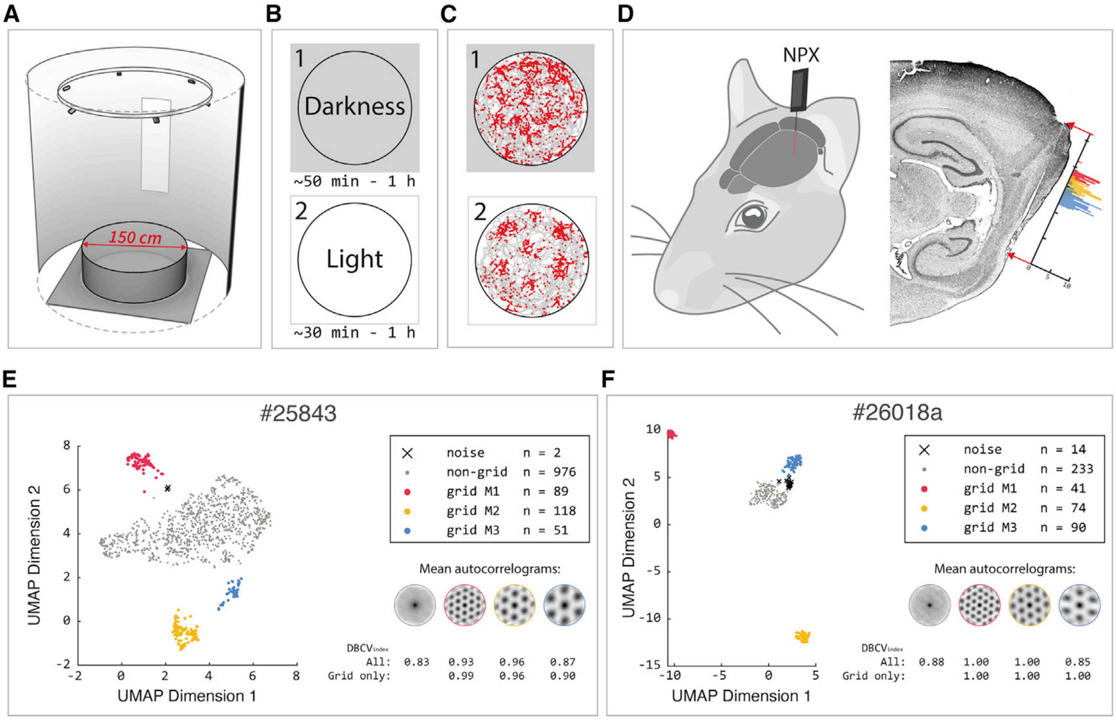

Figure 1. Experimental setup and module classification

(A) Recording arena: electrophysiological recordings of the spiking activity in the MEC were collected while the rat ran freely in a 150 cm diameter cylindrical arena surrounded by a floor-to-ceiling blind and a single, fixed, and tactile inaccessible cue card.

(B) Protocol: first the animals ran in darkness, then in the same arena with the lights on.

(C) The spikes of a representative grid cell from the most dorsal module (red distribution in D) are superimposed in red on the traveled path of the rat in gray, in the dark (1) and light (2) trials.

(D) Left: illustration of implantation site for Neuropixels probe. Right: sagittal section of the rat brain (#26018), showing the probe shank through the superficial layers of MEC. The histogram shows the grid-cell count across dorso-ventral recording depths from three modules. The distance between two adjacent ticks along the probe shank axis corresponds to 1 mm.

(E and F) Module classification for the two recording sessions with the largest number of simultaneously recorded grid cells. Left: for each recording, scatterplots show the two-dimensional UMAP (McInnes et al., 2018) projection of all recorded units' autocorrelograms. Each point is color coded by its DBSCAN cluster assignment (Ester et al., 1996). Right: mean autocorrelograms for each cluster and validity (DBCV) index (STAR Methods), including or excluding the non-grid cluster.

blind to eliminate access to distal visual cues, and the experimental protocol was designed to minimize other positional cues (STAR Methods).

Data were collected from four animals and an overall of five recording sessions, each consisting of a 50–60 min recording in the dark immediately followed by a 30–60 min recording in the light (Figures 1B and 1C). Neuropixels probes were implanted in MEC (Figures 1D and S1). Out of 3,310 recorded cells with >500 spikes in the five light trials, 842 grid cells were identified and classified into modules as described in STAR Methods and in Gardner et al. (2022). Briefly, a non-linear dimensionally reduction technique (McInnes et al., 2018) was applied to feature vectors derived from the spatial autocorrelation of each cell-rate map, followed by clustering (Ester et al., 1996). The procedure ensures modular separation as grid cells with similar auto-correlograms, and thus similar spacing and orientation, are separated into distinct clusters, whereas cells lacking a spatially periodic tuning feature are separated from the grid-cell clusters. In addition to overcoming problems from traditional classification methods caused by skewed grid patterns, false positives would only occur in the unlikely event that the auto-correlogram of a non-grid cell randomly has a pattern with the same spacing and orientation as the real grid cells. Using this procedure, a clear and unambiguous clustering of grid cells into modules was observed in all the sessions (Figures 1E, 1F, and S2). In four out of five recording sessions, many simultaneously recorded grid cells (ranging from 31 to 118 in individual modules) were obtained from three distinct modules, and in one session, such data were obtained from two distinct modules (Table 1).

Several measures indicated that the association between grid-cell activity and the position of the animal deteriorated in the sensory-deprived condition. The characteristic periodicity of grid cells was significantly disrupted in the dark trials (Figures 2A and S3A). Gridness score and information content were considerably reduced compared with the baseline light trials (Figures 2B and 2C). In addition, we decoded position from the population activity patterns and compared the magnitude of decoding errors in the light and dark trials. We have done so using two types of decoders that are used later in the manuscript and are described in STAR Methods. The mean absolute error

Table 1. Numbers of simultaneously recorded grid cells (allocated to modules) for each recording session

Recording session	Module 1	Module 2	Module 3
#25843	89	118	51
#26018a	41	74	90
#26018b	40	68	78
#26820	31	44	35
#26718	47	36	–

(MAE) of the decoded position relative to the animal's true position was substantially larger in dark trials than in baseline light trials (Figures 2D and 2E). This was consistent when the decoding of simultaneous spike trains from dark trials was performed using either light- or dark-generated rate maps (Figures S3B and S3C) and across the two decoders. For this reason, and since dark-generated rate maps are degraded, the rate maps used for decoding (both in light and dark trials) from here onward were extracted from the full extent of the light trials.

Pairwise correlations

As a first step to address the question of inter-module coordination, we considered pairwise correlations of the spiking activity, smoothed with a 50 ms Gaussian kernel (spike-rate correlations, STAR Methods), similar to previous works that were based on tetrode recordings (Almog et al., 2019; Chen et al., 2016; Fyhn et al., 2007; Gardner et al., 2019; Pérez-Escobar et al., 2016; Trettel et al., 2019; Yoon et al., 2013). Differences in the correlation structure of inter- versus intra-module pairs were expected under light conditions for the following reason: the activity of intra-module grid-cell pairs is either correlated or anti-correlated irrespective of the position of the animal, whereas the activity of inter-module grid-cell pairs can be correlated in some parts of the environment and uncorrelated in others (Figure 3A). Consequently, weaker absolute spike-rate correlations were expected on average in inter-module pairs compared with intra-module pairs. Indeed, under light conditions, intra-module pairs showed higher absolute spike-rate correlations compared with inter-module pairs (Figure 3B). The observed zero-lag correlations could be explained quite well by the cells' rate maps and the animal's trajectory, even in inter-module pairs (Figure 3C). Thus, the correlations observed in the spiking activity of inter-module pairs were weak but still driven, to a large extent, by their spatial selectivity.

Having established that spike-rate correlations are related to spatial selectivity both in intra-and inter-module pairs, we next compared pairwise spike-rate correlations in dark and light trials. We reasoned that if, in darkness, grid cells cease to consistently encode a unique spatial location, their spike-rate correlations would diminish. The absolute magnitude of the spike-rate correlations in *intra*-module pairs was similar, on average, to that observed in the light (Figure 3D), even when, in the dark, the spatial stability of single-grid cell representations was disrupted. This is in accordance with recordings performed in mice in dark environments (Chen et al., 2016; Pérez-Escobar et al., 2016), with results found in sleep (Gardner et al., 2019), and as expected based on CAN models. However, the average absolute magnitude of spike-rate correlations in *inter*-module pairs was similar in light and dark conditions as well. Furthermore, zero-lag correlations were preserved between light and dark conditions at the level of individual cell pairs both for inter- and intra-module pairs (Figure 3E).

The preservation of inter-module spike-rate correlations in the dark supports the hypothesis that modules maintain coordination even in the absence of sensory cues. However, it is difficult to interpret this result quantitatively, since inter-module spike-rate correlations are already low even in the light and may be influenced also from sources other than the correlation between their spatial receptive fields, such as co-fluctuation of firing rates in the entire population (Okun et al., 2015; Figures S4C and S4D). Analysis of our simultaneous recordings from dozens of grid cells per module (Table 1) could potentially overcome these limitations by revealing higher-order dependencies in the activity of cells from different modules that are not strongly evident in spike-rate correlations within pairs of cells. Therefore, we next analyzed the recorded simultaneous population activities using two complementary approaches.

Likelihood of the simultaneous population spike trains

In the absence of sensory cues and under the hypothesis of inter-module coordination, a unique position should be coherently represented by the joint activity of grid cells in different modules, even when spatial firing patterns of individual grid cells seem disrupted. When the joint representation is read out under these conditions, it can, however, deviate substantially from the animal's true position compared with baseline light trials. We therefore sought to identify a measure for the coherence of the joint simultaneous spike trains, which is *independent* of the animal's true position.

In the first analysis approach, we derived the likelihood of the simultaneously recorded spike trains, summed over all possible trajectories (thus, independently of the actual trajectory) under simple assumptions that are outlined below. This likelihood is written as

$$p(\boldsymbol{S}_t) = \sum_{\boldsymbol{X}_t} p(\boldsymbol{X}_t) \cdot p(\boldsymbol{S}_t | \boldsymbol{X}_t) \qquad \text{(Equation 1)}$$

where $\boldsymbol{S}_t$ represents the simultaneous spike trains emitted by all the neurons in the population from the beginning of the experiment up to time t, and $\boldsymbol{X}_t$ represents a particular trajectory of the animal. The likelihood of the spike trains conditioned on the trajectory, $p(\boldsymbol{S}_t|\boldsymbol{X}_t)$, is evaluated under the assumption of Poisson firing, with a rate that is determined by $\boldsymbol{X}_t$ and by the tuning curves of all the neurons in the population. Finally, we assumed that the trajectories are continuous (following random walk statistics for simplicity), enforced through the prior $p(\boldsymbol{X}_t)$ (see also STAR Methods).

On the right-hand side of Equation 1, a probability is assigned to each particular realizable trajectory irrespective of the spike trains, followed by a multiplication with the probability of the simultaneously recorded spike trains conditioned on this particular trajectory. This probability is subsequently averaged over all possible trajectories, weighted by the prior. The outcome $p(\boldsymbol{S}_t)$ can be interpreted as a measure that describes the likelihood

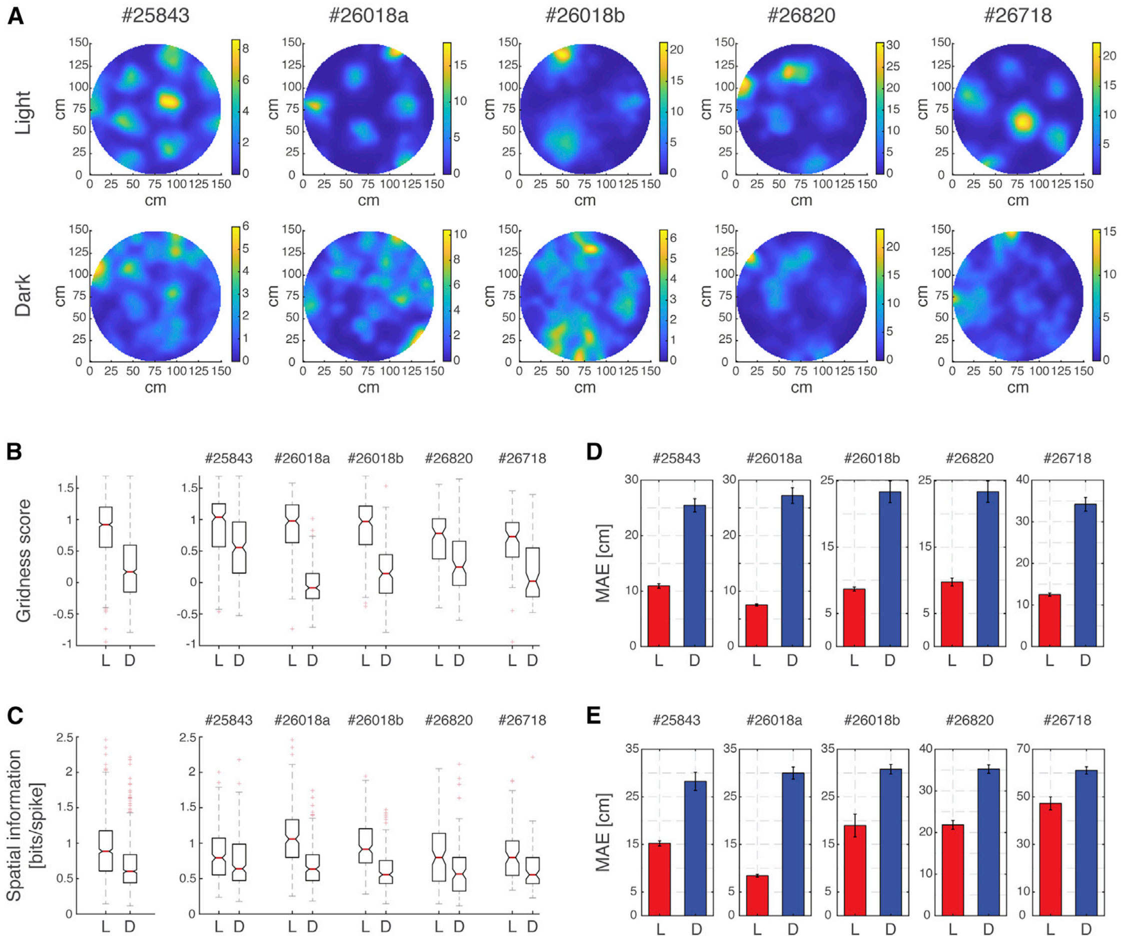

Figure 2. Grid-cell spatial responses deteriorate in darkness

(A) Example of rate maps from each session for light and darkness conditions. Three more examples from each recording session are shown in Figure S3A.

(B) Gridness scores in light and darkness for all cells across all recording sessions (left) and for all cells from single recording sessions (right). Red lines indicate median, and the lower and upper box limits indicate 1st and 3rd quartiles, respectively. Whisker lengths indicate 1.5 times the interquartile range. Red crosses show outliers that lie more than 1.5 times outside the interquartile range. Notches indicate 95% confidence interval of the median.

(C) Spatial information in light and darkness for all cells across all recording sessions (left) and for all cells from single recording sessions (right). Box plots are plotted as in (B).

(D) Mean absolute error (MAE) of the Markov decoder (STAR Methods) in light (red) and darkness (blue) for each recording session. Error bars are ±SEM.

(E) Same as (D) but for the kernel decoder (STAR Methods).

that the simultaneously recorded spike trains represent some continuous (yet unknown) trajectory, drawn from the prior distribution. Importantly, in a practical implementation there is no need to explicitly calculate the specific probabilities for each of the possible trajectories, which would be unfeasible. Instead, we derived a simpler, exact analytical expression for the average log likelihood per time bin, denoted by L (STAR Methods; Equation 3). The evaluation of L using this expression relies on the Markov property of the spiking model and the prior $p(\boldsymbol{X}_t)$ and involves decoding of the spiking activity using a *Markov decoder* (STAR Methods; Methods S1).

In the expression for the likelihood (Equation 1), it is assumed that all the neurons fire in response to the same trajectory $\boldsymbol{X}_t$, regardless of the identity of the module to which they belong. If this assumption is correct, then this trajectory, as well as nearby trajectories, will make a large contribution to the likelihood (Figure 4A). However, if different modules accrue drifts independently in the dark and thus represent internally different trajectories, there will be no single trajectory in the sum within Equation 1 that makes a large contribution to the likelihood, and we expect the sum to be significantly smaller. Thus, the likelihood introduced above (Equation 1) can serve as a measure of coherence.

To validate that the likelihood can be used to distinguish between the scenarios of coordinated and uncoordinated drifts across modules, we first analyzed simulated spike trains. All neurons in the simulated data fired according to the same recorded trajectory and based on measured tuning curves, which were taken from one of our datasets. Therefore, the different modules were precisely coordinated in the simulation. To mimic the consequences of uncoordinated drifts across modules in a way that can be applied also to recorded datasets, we introduced artificial spatial shifts during the decoding process in the neuron's rate

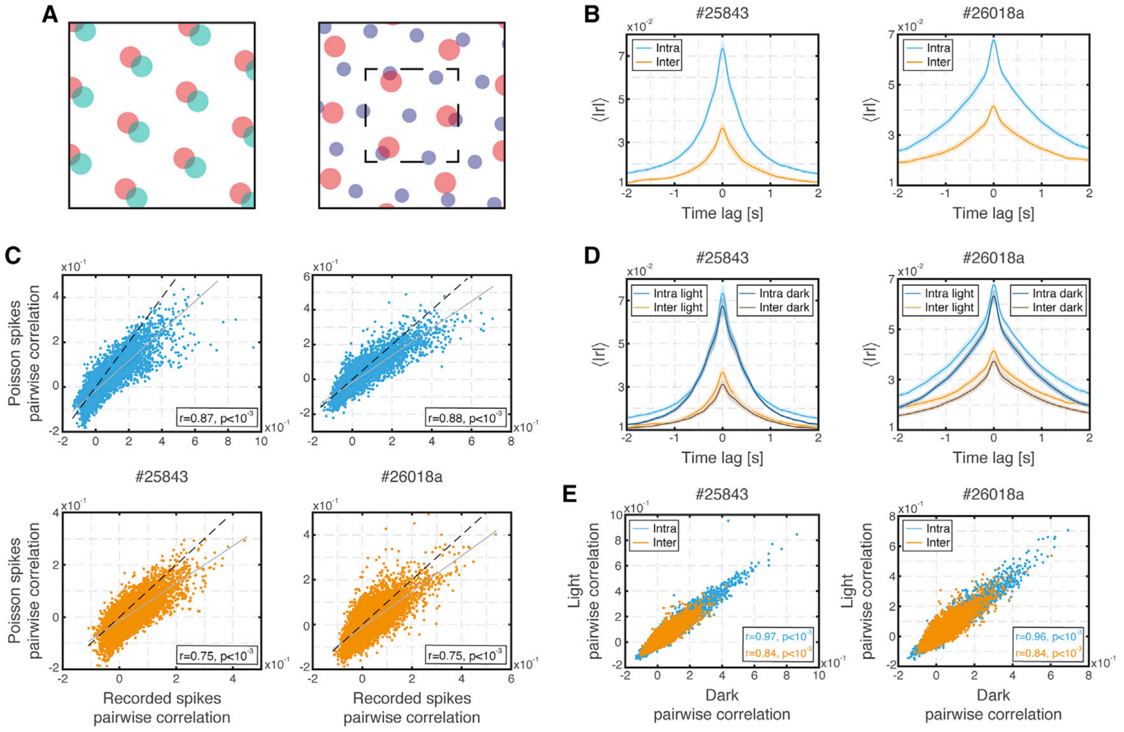

Figure 3. Spike-rate correlations of intra- and inter-module pairs

(A) Schematic illustration showing spatial tuning curves of two intra-module (left, red, and turquoise) and two inter-module (right, blue, and red) grid cells. Firing fields of intra-module pairs (left) either overlap throughout the whole environment (as shown in the figure) causing temporally correlated firing or are disjoint throughout the whole environment causing anti-correlated firing. In contrast, in inter-module pairs (right), the degree of correlation (or anti-correlation) between firing fields varies in different parts of the environment: firing fields overlap inside the small dashed square and are disjoint elsewhere. Consequently, absolute spike-rate correlations tend to be weaker in inter-module pairs than in intra-module pairs. This effect is more pronounced in large environments compared with small environments (Figures S4A and S4B).

(B) Absolute cross-correlation (Pearson coefficient) of inter- and intra-module light spiking activities, averaged over cell pairs, in the two recording sessions with the largest number of simultaneously recorded neurons (left and right panels). Shaded error bars are ±SEM.

(C) Pairwise correlations of all possible intra- (top) and inter- (bottom) module pairs from recorded light spiking activity versus Poisson generated spikes using measured rate maps and the corresponding recorded light trajectory. Correlation coefficient and p value are specified in the inset. Fit shown in solid gray and the identity line is shown for reference (dashed black line).

(D) Same as (B) but with superimposed inter- and intra-module spiking activities from darkness.

(E) Pairwise correlations of all possible inter- and intra-module pairs from recorded light spiking activity versus recorded dark spiking activity.

maps (STAR Methods) that, for simplicity, were constant throughout each simulation. Such shifts were identical within each module but drawn randomly and independently in different modules (and were thus uncoordinated across modules).

We observed that applying independent spatial shifts reduced the likelihood (Figure 4B, black trace). As expected, such shifts also increased the MAE of the decoder with respect to the true position (Figure 4C, black trace). In contrast, the likelihood was nearly unaffected when the artificial spatial shifts were identical across all modules, even though the MAE with respect to the true position increased significantly (Figures 4B and 4C, gray traces). The small reduction in the likelihood seen in Figure 4B (gray trace) is due to boundary effects and vanishes when such effects are eliminated (Figure S4E; see also Figure S6B below). The application of spatial shifts to the neuron's rate maps during decoding is analogous to application of shifts in the encoded position (Figure S4F; Note that the latter can be applied only to simulated data). The results shown in Figure 4 confirmed that the average log likelihood could be used as a measure of coherence of the simultaneous spike trains.

We next applied the likelihood-based approach to recorded datasets from the light and dark conditions (5 recordings sessions from 4 animals; Table 1) to assess whether modules remain coordinated in the dark. If, in the dark, the phases of individual modules accrued independent drifts relative to the animal's true position, a reduction in the likelihood would be expected relative to the light. To faithfully compare likelihoods between light and dark conditions, it was necessary to take into account modifications in the mean firing rates of individual neurons across the two conditions: specifically, mean firing rates were more likely to reduce than increase in darkness, resembling previous results from mice (Pérez-Escobar et al., 2016). Thus, we evaluated *rate-adjusted* likelihoods of dark and light simultaneous spike trains, obtained by down-sampling spikes to match the mean firing rates between the two trials (STAR Methods; Methods S1), henceforth referred for convenience as likelihood.

CellPress
OPEN ACCESS

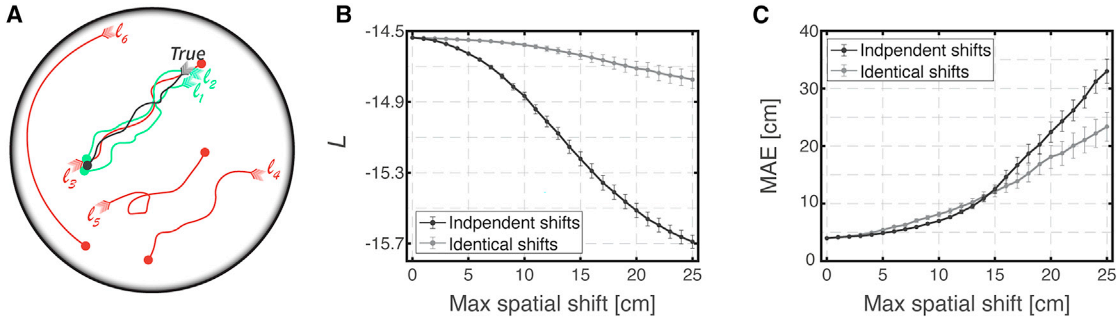

Figure 4. Likelihood of simultaneous spike trains can serve as a measure of coordination across modules

(A) Schematic illustration demonstrating the likelihood approach with a few illustrated trajectories, each starting from an arrowhead and ending in a point. The true trajectory of the animal is the black trace. The likelihood of the simultaneous spike trains is evaluated independently for each specific trajectory and is averaged across all possible trajectories. A few possible representative trajectories are illustrated: trajectories l_1 and l_2 (turquoise) are two trajectories which are close to the true trajectory and thus have a high likelihood based on the spiking activity. Trajectory l_3 is very similar to trajectories l_1 and l_2 but has low likelihood (red) since it goes in the opposite direction, thus having reversed temporal structure. Trajectory l_4 is an identical copy of trajectory l_2 but at a different part of the arena, thus also having low likelihood (red). Trajectories l_5 and l_6 are two other trajectories with low likelihood (red).

(B) Likelihood of simulated Poisson spikes using measured rate maps and the recorded light trajectory from recording session #26018b, evaluated versus varying magnitudes of spatial shifts. When shifts are applied independently in each module (black trace), the likelihood decreases significantly, but it decreases only slightly (due to boundary conditions) when these shifts are identical (gray trace). Error bars are ±SEM.

(C) The corresponding mean absolute error (MAE) of the decoder. The MAE increases significantly both for independent and for identical spatial shifts as their magnitude increases. Error bars are ±SEM.

We found that the likelihood of simultaneously recorded spike trains in light trials was slightly higher than the likelihood in dark trials, although the MAE was much larger in darkness than in baseline light trials (Figure 5A, zero spatial shift). To demonstrate that the observed similarity in likelihoods was not simply an outcome of the rate-adjustment procedure, we also considered permuted, rate-adjusted spike trains, which preserved the mean firing rates. Under such a permutation, the evaluated likelihood decreased drastically and the MAE drastically increased (Figure S5A), demonstrating the importance of temporal structure of the simultaneous spike trains. To assess the significance of the small observed likelihood differences in relation to the question of module coordination, we evaluated the expected reduction in the likelihood under independent spatial shifts of varying magnitudes, as in Figures 4B and 4C. We found that the spatial shifts required to reduce the likelihood in the light to the value observed in the dark (point p_1 in Figure 5A, left) would only generate a small increase in the MAE—of a few centimeters (point p_2 in Figure 5A, right), whereas much larger spatial shifts would be required to match the actual MAE observed in the dark when using zero spatial shifts. Thus, the small reduction in the likelihood in the dark recordings at zero spatial shifts relative to the light is consistent with only small independent shifts across modules, of a few centimeters.

Conversely, the spatial shifts required to increase the MAE in the light to the value observed in the dark (point q_1 in Figure 5A, right) would produce a substantial decrease in the likelihood, if applied in an uncoordinated manner to the spike trains from the light recordings (point q_2 in Figure 5A, left), to a value that is much smaller than the zero-shift likelihood observed in the dark recordings. Thus, the increase in the zero-shift MAE observed in the dark recordings must arise mostly from coordinated drifts across the modules. To validate that coordinated drifts across modules can increase the MAE without significantly reducing the likelihood, we introduced identical spatial shifts across all modules as in Figures 4B and 4C. As expected, the likelihoods of dark and light trials were only slightly reduced, whereas the corresponding MAE's increased substantially (Figure 5B). As in Figure 4B, the small reduction observed in the likelihoods with the introduction of coordinated spatial shifts is due to boundary conditions.

Results for additional datasets are shown in Figures 5C and S6A. Similar results were obtained when applying spatial rotations instead of spatial shifts (Figure S6B). Finally, we verified that differences in the motion statistics between light and dark trials are not expected to substantially affect the likelihood (Figure S5B). Movies showing typical examples of decoding from light and dark trials are shown in Videos S1 and S2.

Decoding from individual modules

The likelihood-based approach described above can be applied to datasets with moderate numbers of cells per module, where the decoded position is very noisy. In most of our datasets, the large number of simultaneously recorded cells enabled testing for inter-module coordination using a more direct approach, based on the decoding of position from individual modules.

In this approach, which is schematically illustrated in Figure 6A, an internal multi-module representation of position, denoted by $\hat{m}$, was first estimated by decoding spiking activity from all the grid cells. We used a *kernel decoder*, which estimates position based on spikes within a fixed time window, unlike the Markov decoder which has access to the entire spiking history (STAR Methods). As expected, the multi-module posterior typically exhibited a single prominent peak within the enclosure, which could potentially deviate from the true position of the animal (X in Figure 6A). Next, spiking activity from each module was decoded separately. As expected, single-module posteriors

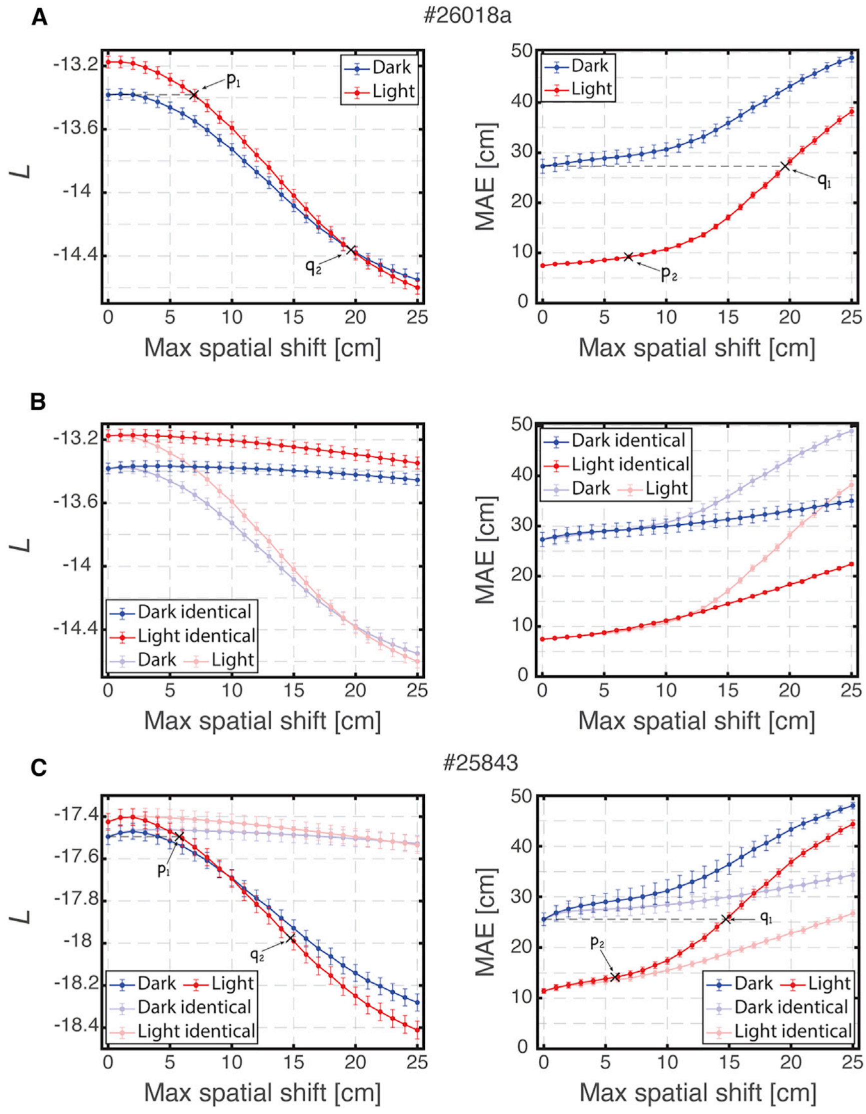

Figure 5. Analysis of likelihood of recorded simultaneous spike trains indicates coordination across modules

(A) Likelihood of simultaneously recorded spike trains (left) and corresponding mean absolute error (MAE, right) from dark and light trials, shown for varying magnitudes of independent module-wise spatial shifts applied on a single recording session (#26018a). Applying a maximal spatial shift of 6.9 cm in the light recording achieves the same likelihood as that of the dark recording with zero spatial shift (point p_1, left panel) but generates only a slight increase of less than 2 cm in the corresponding MAE of the light recording relative to its zero spatial shift value (point p_2, right panel). Conversely, applying a maximal spatial shift of 19.6 cm in the light recording achieves the same MAE as that of the dark recording with zero spatial shift (point q_1, right panel) but generates a dramatic decrease in the likelihood (point q_2, left panel). The difference in the likelihood between point q_2 and the zero spatial shift point of the dark recording is much larger than the difference between the likelihood values of light and dark zero spatial shift points. The MAEs under the null hypotheses (STAR Methods) for the light and dark trials are $\sim$ 68 and $\sim$ 66 cm, respectively. Error bars are ±SEM.

(B) Same as (A) but with identical spatial shifts in all modules (full color traces; results for independent spatial shifts, same as in (A), are superimposed using faded colors for comparison). Under identical spatial shifts the dark and light likelihoods decrease only slightly (due to boundary conditions, left), whereas the corresponding MAE increases significantly in both cases (right).

(C) Same as (A) but for another recording session (#25843) and with identical spatial shifts plotted in faded colors. The MAEs under the null hypotheses for the light and dark trials are $\sim$ 69 and $\sim$ 66 cm, respectively.

typically exhibited approximately periodic peaks. To resolve this ambiguity, the position which maximized the posterior within the local vicinity of the position $\widehat{m}$ was selected as the corresponding decoded position $\widehat{u}_i$ for each module i (STAR Methods). Finally, the distances between the position $\widehat{m}$ to each position $\widehat{u}_i$ (denoted by δ_i) and between $\widehat{u}_i$ pairs (denoted by Δ_{ij}) were evaluated and compared between dark and baseline light trials (Figure 6A right). Typical examples of decoding from light and dark trials are shown in Videos S3 and S4.

The MAE of position $\widehat{m}$, with respect to the animal's true position, was higher in darkness than in baseline light trials (one example shown in Figure 6B), indicating that the internal representation of position in the dark drifted relative to the true position. The distances δ_i and Δ_{ij} were noisy and fluctuated in time both in dark and light conditions, but their mean was only slightly higher in darkness than in light (Figure 6B). This is consistent with the results from the previous approach (Figure 5), which demonstrated slightly higher likelihoods for simultaneously recorded light spike trains compared with corresponding dark trials.

Since the single-module readout positions $\widehat{u}_i$ were restricted to a vicinity of the position $\widehat{m}$, which, by definition, best agrees with activities from all modules, it was important to verify that the similarity of distances in light and dark trials was not an inevitable consequence of the methodology. Therefore, we introduced independent spatial shifts in the rate maps of all neurons that belong to the same module during the decoding process in a similar fashion as performed in the previous likelihood approach. We found that the mean distances δ_i and Δ_{ij} increased dramatically as the magnitude of spatial shifts increased, indicating that these measured distances could have potentially been much higher than observed and did not arise simply because of the selection of positions $\widehat{u}_i$ in proximity to $\widehat{m}$ (Figures 6C and 6D). Therefore, the preservation of these small distances in the dark, while the position $\widehat{m}$ deviated substantially from the animal's true position (Figure 6E), is an explicit indication of tight

CellPress
OPEN ACCESS

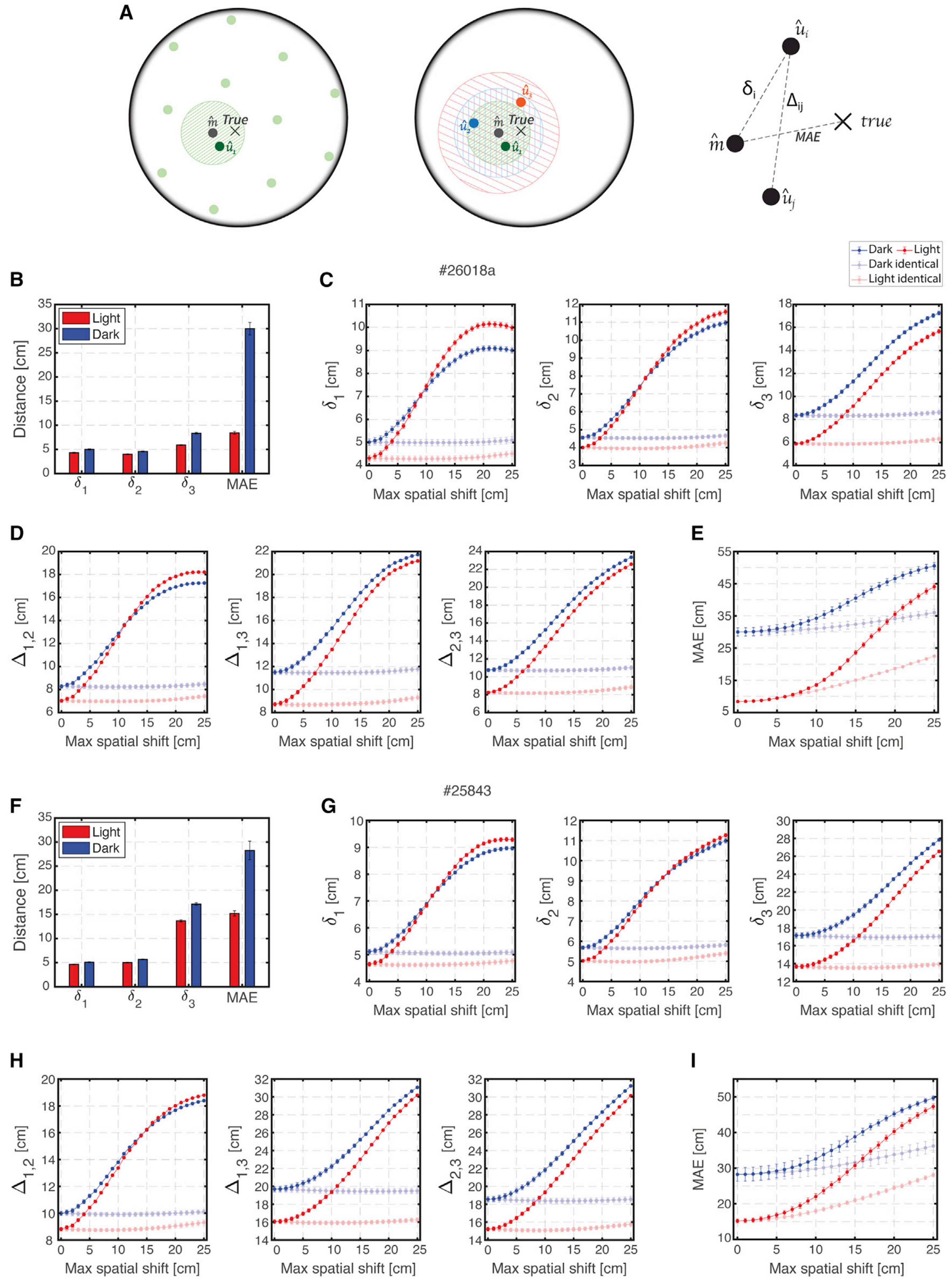

Figure 6. Decoding of population activity from individual modules reveals tight coordination of phases across modules in darkness

(A) Schematic illustration of the uni-module decoding approach. Left: decoding the spiking activity from all grid cells typically produces a posterior with a unique blob in the arena, and the position of its maximum is chosen as the estimate of the multi-module internal representation (gray circle $\hat{m}$). This estimation can deviate from the true position of the animal (black X symbol). Decoding the spiking activity from a single module typically produces a periodic posterior (faded green circles). The blob that is nearest to position $\hat{m}$ is selected (dark green circle), and the position of its maximum is chosen as the estimate of the uni-module internal

(legend continued on next page)

coordination between the modules. As expected, identical spatial shifts did not affect δ_i and Δ_{ij}, although they dramatically increased the MAE (faded traces in Figures 6C–6E). Similar results from additional datasets are shown in Figures 6F–6I and S7.

We finally tested whether module coordination remained tight in darkness, specifically during periods in which the multi-module readout $\hat{m}$ deviated substantially from the true position of the animal. To address this question, we focused on non-overlapping continuous segments of the dark recordings in which the MAE was particularly high and on non-overlapping continuous segments within the same recording in which the MAE was particularly low (STAR Methods). Although the deviation of the internal representation from the true position was in the order of $\sim$ 30 cm in the high-MAE segments (compared with order of $\sim$ 5 cm in the low-MAE segments), the distances δ_i remained small as in the low-MAE segments (Figure 7A). Importantly, these distances δ_i could have potentially been much higher, as demonstrated above (Figures 6C and 6D). This result indicates that representations within individual modules did not accrue any additional, significant relative drifts even when the multi-module representation of position deviated substantially from the animal's true position.

Furthermore, we considered all time points from dark trials and examined the joint distributions of δ_i and the MAE and the joint distributions of Δ_{ij} and the MAE. We expected that if modules are coordinated then the conditioned distributions of δ_i and Δ_{ij} will be nearly independent of the MAE and in particular remain narrowly distributed even when the MAE is large. Figure 7B demonstrates that, indeed, the distances δ_i and Δ_{ij} were distributed nearly identically for different values of the MAE. This is yet another explicit indication that coordination between modules remains tight, even when the internal representation of position deviates from the true position of the animal. Results for additional datasets are shown in Figures 7C–7H.

DISCUSSION

In contrast to the rigid relationships in activity of cells within a module, activity in different modules spans diverse phase combinations, allowing them to represent a large range of positions and environments. Nevertheless, here we showed that dynamically, the phases of different modules are coupled. Even when the internal representation of position in the MEC deviates substantially from the true position of the animal, updates to the phases remain coordinated across the different modules, thus maintaining a coherent representation of a two-dimensional trajectory.

The likelihood-based approach and the uni-module decoding approach both led to the conclusion, consistently across different animals and sessions, that the inter-module phases are dynamically coupled. Both methods also pointed to a small increase in the mismatch between modules in darkness compared with light conditions, indicating that sensory inputs help coordinate the states of different modules. The picture that emerges from these results is that when sensory inputs are poor or absent, small mismatches can develop in the phases of different modules, but internal brain mechanisms prevent these mismatches from accruing over time, thus maintaining a coordinated and coherent representation across the full grid-cell population. It has been previously hypothesized that such internal mechanisms may exist (Burak, 2014; Welinder et al., 2008), possibly supported by recurrent synaptic connectivity within the MEC (Kang and Balasubramanian, 2019; Mosheiff and Burak, 2019) or by the reciprocal synaptic connectivity of the MEC with the hippocampus (Agmon and Burak, 2020; Sreenivasan and Fiete, 2011; Welinder et al., 2008). It will be of great interest to explore these underlying mechanisms in future studies, for example, by testing whether inputs from the hippocampus are required to maintain phase coordination.

The analyses of the recorded spike trains relied on simplifying assumptions. Grid cells are modeled as independent units, which emit Poisson spike trains that are solely dictated by their spatial selectivity, alongside a random walk prior (in the likelihood approach). However, the actual mechanisms that govern grid-cell activities are more complex. Therefore, the decoders are not optimal (or tailored) for the actual recorded data. Note, however, that we do not compare decoding performance of simulated and recorded spike trains, but instead compare the decoding performance of recorded light and dark spike trains. This enables us to reliably quantify and compare the coherence of recorded population activities across the dark and light conditions. Our spatial shift controls, which induce inter-module incoordination, are directly generated from the recorded data and thus do not require a comparison of the quantified coherence from the recorded data with that obtained from simulated spike trains. Altogether, these analyses indicate that internal brain mechanisms enforce coordination between distinct grid-cell modules.

representation, denoted by $\hat{u}_1$. Middle: this procedure is repeated for each individual module (green, orange, and blue) producing an estimated uni-module position for each module ($\hat{u}_1$, $\hat{u}_2$, and $\hat{u}_3$). Right: the distance from position $\hat{m}$ to the true position of the animal is averaged over time to produce the mean absolute error (MAE). The mean distance between position $\hat{m}$ to each position $\hat{u}_i$ is defined as δ_i, and the mean distance between each pair of positions $\hat{u}_i$ and $\hat{u}_j$ is defined as Δ_{ij}.

(B) The measured MAE and distances δ_i for dark and light from a single recording session (#26018a). The distances δ_i in darkness are only $\sim$ 1 cm larger than those in baseline light trials. Error bars are ±SEM.

(C) The distances δ_i for varying magnitudes of module-wise independent spatial shifts and for identical spatial shifts. Increasing the magnitude of module-wise independent spatial shifts increases δ_i dramatically, indicating that these measured distances δ_i could potentially be much higher. The same magnitude of identical spatial shifts has no effect. The δ_is under the null hypotheses (STAR Methods) for the light and dark trials are $\sim$ (13.7, 18.2, 25.5) cm and $\sim$ (13.8, 18.3, 25.7) cm, respectively. Error bars are ±SEM.

(D) Same as (C) but for the corresponding distances Δ_{ij}.

(E) Similar as (C) but for the corresponding MAE. The MAE increases for both module-wise independent and identical spatial shifts.

(F–I) Same as (B)–(E) but for another recording session (#25843). The δ_is under the null hypotheses for the light and dark trials are $\sim$ (16.2, 25.6, 32.7) cm and $\sim$ (16.4, 25.7, 32.8) cm, respectively.

CellPress
OPEN ACCESS

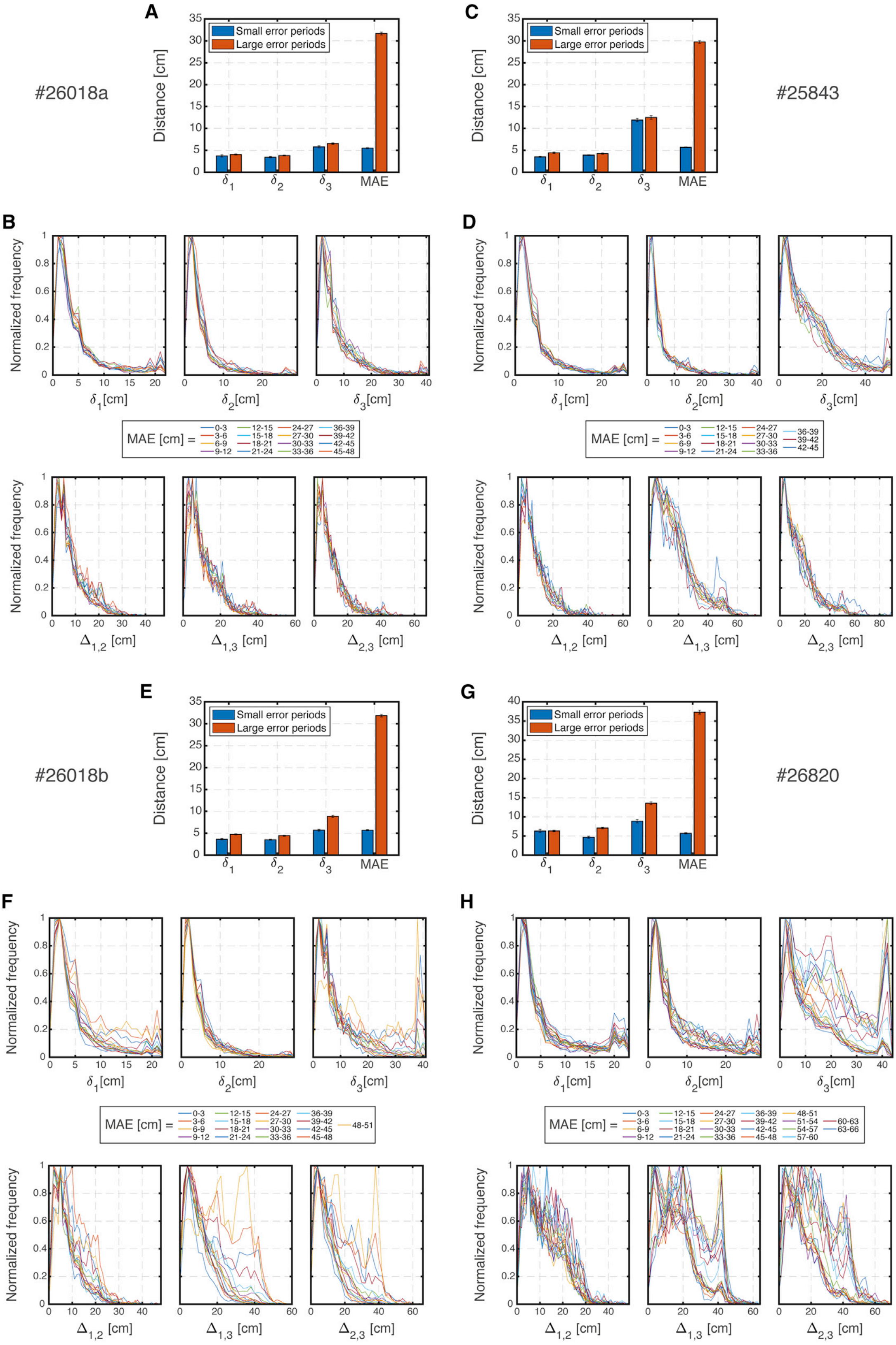

(legend on next page)

Despite a major reduction of sensory cues, their elimination was probably not complete. Although the arena was carefully cleaned during and between trials, leftover olfactory cues may have provided some spatial information. The rare encounters with the walls could also provide limited spatial information (Hardcastle et al., 2015); yet, it is unlikely that absolute position could be inferred from such encounters, as the arena was rotationally symmetric. Although the elimination of sensory cues was probably imperfect, the disruption of the spatial rate maps and the increased MAE of decoded population activities in darkness (Figure 2) demonstrated that a large mismatch between internal representation and true position was typical under these conditions. Importantly, the typical mismatch was in the order of tens of centimeters, whereas the misalignment between modules was in the order of a few centimeters, even during periods in which the MAE was particularly large (Figure 7). The likelihood approach (Figure 5) points to similar conclusions because uncoordinated spatial shifts of more than a few centimeters applied onto the dark recording have led to a large decrease in its likelihood, relative to the baseline difference between the light and dark likelihoods.

Previous studies that examined grid-cell activity in mice in dark environments (Chen et al., 2016; Pérez-Escobar et al., 2016) have shown that even when the periodic firing patterns of grid cells lose their spatial stability, grid cells within a module preserve the pairwise spiking correlations that they exhibited in the light. However, these works did not test whether the coactivation patterns of distinct modules are coordinated during darkness. Due to the recording technique (classical tetrodes), simultaneously recorded cell pairs from distinct modules were rare, and the data collected did not enable analysis based on population decoding as performed in the present work.

The spatial response patterns of individual grid cells in the mouse studies (Chen et al., 2016; Pérez-Escobar et al., 2016) were more strongly degraded in darkness than those observed in rats in our study (Figures 2B and 2C). These differences might possibly emerge from different dead-reckoning capabilities of rats and mice: unlike mice, rats have been shown to exhibit stable grid fields in darkness (Hafting et al., 2005). Consequentially, it was necessary in our study to take extreme measures (large circular arena, removing odors) to obtain strong degradation of the grid response patterns. The decoding results (Figures 2D and 2E) confirmed that our rats were indeed disoriented in darkness, even when the dark data were decoded from dark-generated rate maps (Figures S3B and S3C). Previous work in the hippocampus (Bjerknes et al., 2018) has demonstrated excellent ability of place cells to express precisely localized fields on a linear track in darkness, under conditions that ensured absence of any relevant external sensory information. Therefore, it is possible that with scarce remaining sensory cues, rats can maintain spatial maps—in hippocampus and MEC—more accurately than mice. Another possibility is that differences in the degradation of grid responses result from subtle differences in the environments or training protocols used by the mouse and rat studies. However, the origin of these differences in the precision of spatial representation during darkness is not the subject of this study. What is crucial for the study of coordination between grid-cell modules is that substantial disorientation was achieved.

Previous work (Stensola et al., 2012) pointed to a functional independence in the response of modules to an abrupt environmental deformation (Barry et al., 2007) in which the enclosure was compressed by moving one of the walls. A key finding was that this manipulation resulted in compression of the rate maps that occurred in some modules but not in others. Thus, the distinct responses of different modules to the environmental deformation were indicative of functional independence in their dynamics. Nevertheless, the implications of this result for the dynamics of module coordination are not yet sufficiently clear: one possibility is that even shortly after the environmental deformation, grid-cell firing remains anchored to position. In this case, the rates of phase updates, as a function of position, are modified compared with baseline conditions. Under this interpretation of the experiment, the dynamical coordination of modules is disrupted everywhere within the enclosure, possibly transiently. Alternatively, it has been suggested (Keinath et al., 2018; Ocko et al., 2018) that module phases are updated abruptly upon encounters with the walls due to interactions with border cells. Under this interpretation of the experiment, rate maps are altered due to spatial shifts of the grid firing fields that depend on recent encounters with the walls. Yet, the phase update rates remain largely unmodified within the interior of the environment, and dynamical module coordination remains intact between encounters with the walls. Based on the tetrode recordings that were available in the deformation experiment (Stensola et al., 2012), it is difficult to conclusively distinguish between these possibilities. To do so, it will be beneficial to decode module phases from population activity patterns and analyze their joint dynamics, utilizing large numbers of simultaneously recorded grid cells from different modules.

The analysis in this work relied on the ability to simultaneously record spikes from dozens to hundreds of grid cells and our results demonstrate the power of this technique in elucidating dynamics within large neural circuits (Chaudhuri et al., 2019; Gallego et al., 2018; Gardner et al., 2022; Pfeiffer and Foster, 2013). With several dozens of cells from each module, decoding of phases from single modules was sufficient to obtain strong measures of coordination between the modules, based on statistics that were collected across long periods of

Figure 7. Module population activity patterns are tightly coordinated even during high-MAE periods

(A) The measured mean absolute error (MAE) and distances δ_i during large- and small-MAE periods from a single recording session (#26018a) during darkness. Error bars are ±SEM.

(B) Top: corresponding normalized distributions of the distances δ_i for varying values of the MAE. Bottom: same as top but for distances Δ_{ij}.

(C and D) Same as (A) and (B) but for another recording session (#25843). Note that outliers in (D) are traces with largest MAE.

(E–H) Same as (A) and (B), for two additional recording sessions (#26018b and #26820) in which recordings were obtained from three modules. Note that, although recordings from three modules were available in these datasets, the numbers of simultaneously recorded grid cells were relatively small (especially in recording session #26820; Table 1), leading to inaccurate decoding.

motion. Future studies, with even larger numbers of simultaneously recorded cells, may enable more precise dynamical tracking of the states of individual modules over single trials. Such analysis may include extraction of toroidal coordinates from the joint activity in each module independently of the spatial selectivity (Gardner et al., 2022), which requires more simultaneously recorded cells than was available for most modules in the present study. With such finer temporal and spatial resolution, it may be possible to characterize more precisely how the small mismatch that does exist between modules evolves over time, in relation to behavior or external stimuli. Such analysis may further elucidate the mechanisms that underlie coordination between attractor networks in the entorhinal cortex and the hippocampus.

STAR★METHODS

Detailed methods are provided in the online version of this paper and include the following:

- KEY RESOURCES TABLE
- RESOURCE AVAILABILITY
  - Lead contact
  - Materials availability
  - Data and code availability
- METHOD DETAILS
  - Subjects
  - Electrode implantation surgery
  - Electrophysiological recordings
  - Behavioural tracking
  - Behavioural procedures
  - Open-field foraging trials in darkness
  - Open-field foraging task in light
  - Perfusion and histology
  - Spike sorting and single-unit selection
  - Module classification
  - Clustering validation
  - Rate map analysis
  - Gridness score
  - Information content
  - Pairwise correlations
  - Markov decoder
  - Likelihood of simultaneously recorded spike trains
  - Rate-adjusted likelihood
  - Mean absolute error (MAE)
  - Spatial shifts
  - Rotational Shifts
  - Idealized grid cell tuning curves
  - Kernel decoder
  - Uni-module decoding
  - Small and large dark error periods
  - Null hypotheses of MAEs and δ_is
  - SEM of correlated time series

SUPPLEMENTAL INFORMATION

Supplemental information can be found online at https://doi.org/10.1016/j.neuron.2022.03.011.

ACKNOWLEDGMENTS

Y.B. is the incumbent of the William N. Skirball Chair in Neurophysics. This study was supported by a Synergy Grant to Y.B. and E.I.M. from the European Research Council (“KILONEURONS,” grant agreement no. 951319), by grants to Y.B. from the Israel Science Foundation (grant nos. 1319/13, 1978/13, and 1745/18), by a grant to Y.B. from the German-Israeli Foundation for Scientific Research and Development, a FRIPRO grant to E.I.M., a Centre of Excellence grant to M.-B.M. and E.I.M., and a National Infrastructure grant to E.I.M. and M.-B.M., all from the Research Council of Norway (FRIPRO, grant number 286225; Centre of Neural Computation, grant number 223262; NORBRAIN, grant number 295721), the Kavli Foundation (M.-B.M. and E.I.M.), and a direct contribution to M.-B.M. and E.I.M. from the Ministry of Education and Research of Norway. Y.B. acknowledges support from the Gatsby Charitable Foundation. The authors are grateful to Christine Lykken for performing the Neuropixels implantation on one of the animals.

AUTHOR CONTRIBUTIONS

E.I.M. and Y.B. conceived the study. T.W., M.-B.M., and E.I.M. designed the experiments. T.W., R.J.G., V.A.N., and A.N. performed the experiments (surgeries, recordings, and spike sorting). R.J.G. developed Neuropixels data analysis pipelines. H.A. and Y.B. conceived and developed the theory and the computational methodology. T.W. and H.A. performed single-cell analyses. T.W., R.J.G., and V.A.N. performed module clustering (UMAP). H.A. performed the simulations. H.A. analyzed module coordination. H.A., E.I.M., and Y.B. interpreted module coordination results with help from all authors. T.W. and H.A. visualized the data. H.A., E.I.M., and Y.B. wrote the paper with contributions from T.W. and inputs from all authors. R.J.G., M.-B.M., E.I.M., and Y.B. supervised the project. M.-B.M., E.I.M., and Y.B. obtained funding.

DECLARATION OF INTERESTS

E.I.M. is on the Advisory Board of *Neuron*. All other authors declare no competing interests.

Received: September 14, 2021
Revised: January 25, 2022
Accepted: March 9, 2022
Published: April 5, 2022

SUPPORTING CITATIONS

The following reference appears in the supplemental information: Schmitzer-Torbert et al. (2005).

REFERENCES

Agmon, H., and Burak, Y. (2020). A theory of joint attractor dynamics in the hippocampus and the entorhinal cortex accounts for artificial remapping and grid cell field-to-field variability. Elife *9*, e56894.

Almog, N., Tocker, G., Bonnevie, T., Moser, E.I., Moser, M.B., and Derdikman, D. (2019). During hippocampal inactivation, grid cells maintain synchrony, even when the grid pattern is lost. Elife *8*, e47147.

Barry, C., Hayman, R., Burgess, N., and Jeffery, K.J. (2007). Experience-dependent rescaling of entorhinal grids. Nat. Neurosci. *10*, 682–684.

Bjerknes, T.L., Dagslott, N.C., Moser, E.I., and Moser, M.B. (2018). Path integration in place cells of developing rats. Proc. Natl. Acad. Sci. USA *115*, E1637–E1646.

Burak, Y. (2014). Spatial coding and attractor dynamics of grid cells in the entorhinal cortex. Curr. Opin. Neurobiol. *25*, 169–175.

Burak, Y., and Fiete, I.R. (2009). Accurate path integration in continuous attractor network models of grid cells. PLoS Comput. Biol. *5*, e1000291.

Chaudhuri, R., Gerçek, B., Pandey, B., Peyrache, A., and Fiete, I. (2019). The intrinsic attractor manifold and population dynamics of a canonical cognitive circuit across waking and sleep. Nat. Neurosci. *22*, 1512–1520.

Chen, G., Manson, D., Cacucci, F., and Wills, T.J. (2016). Absence of visual input results in the disruption of grid cell firing in the mouse. Curr. Biol. *26*, 2335–2342.

Ester, M., Kriegel, H.-P., Sander, J., and Xu, X. (1996). A density-based algorithm for discovering clusters in large spatial databases with noise. In Proceedings of the 2nd International Conference on Knowledge Discovery and Data Mining, pp. 226–231.

Fiete, I.R., Burak, Y., and Brookings, T. (2008). What grid cells convey about rat location. J. Neurosci. *28*, 6858–6871.

Fuhs, M.C., and Touretzky, D.S. (2006). A spin glass model of path integration in rat medial entorhinal cortex. J. Neurosci. *26*, 4266–4276.

Fyhn, M., Hafting, T., Treves, A., Moser, M.B., and Moser, E.I. (2007). Hippocampal remapping and grid realignment in entorhinal cortex. Nature *446*, 190–194.

Gallego, J.A., Perich, M.G., Naufel, S.N., Ethier, C., Solla, S.A., and Miller, L.E. (2018). Cortical population activity within a preserved neural manifold underlies multiple motor behaviors. Nat. Commun. *9*, 4233.

Gardner, R.J., Hermansen, E., Pachitariu, M., Burak, Y., Baas, N.A., Dunn, B.A., Moser, M.-B., and Moser, E.I. (2022). Toroidal topology of population activity in grid cells. Nature *602*, 123–128.

Gardner, R.J., Lu, L., Wernle, T., Moser, M.B., and Moser, E.I. (2019). Correlation structure of grid cells is preserved during sleep. Nat. Neurosci. *22*, 598–608.

Ghosh, K.K., Burns, L.D., Cocker, E.D., Nimmerjahn, A., Ziv, Y., Gamal, A.E. El, and Schnitzer, M.J. (2011). Miniaturized integration of a fluorescence microscope. Nat. Methods *8*, 871–878.

Guanella, A., Kiper, D., and Verschure, P. (2007). A model of grid cells based on a twisted torus topology. Int. J. Neural Syst. *17*, 231–240.

Hafting, T., Fyhn, M., Molden, S., Moser, M.B., and Moser, E.I. (2005). Microstructure of a spatial map in the entorhinal cortex. Nature *436*, 801–806.

Hardcastle, K., Ganguli, S., and Giocomo, L.M. (2015). Environmental boundaries as an error correction mechanism for grid cells. Neuron *86*, 827–839.

Jun, J.J., Steinmetz, N.A., Siegle, J.H., Denman, D.J., Bauza, M., Barbarits, B., Lee, A.K., Anastassiou, C.A., Andrei, A., Aydın, Ç., et al. (2017). Fully integrated silicon probes for high-density recording of neural activity. Nature *551*, 232–236.

Kang, L., and Balasubramanian, V. (2019). A geometric attractor mechanism for self-organization of entorhinal grid modules. Elife *8*, e46687.

Keinath, A.T., Epstein, R.A., and Balasubramanian, V. (2018). Environmental deformations dynamically shift the grid cell spatial metric. Elife *7*, e38169.

Kim, S.S., Rouault, H., Druckmann, S., and Jayaraman, V. (2017). Ring attractor dynamics in the *Drosophila* central brain. Science *356*, 849–853.

Kropff, E., Carmichael, J.E., Moser, M.B., and Moser, E.I. (2015). Speed cells in the medial entorhinal cortex. Nature *523*, 419–424.

Langston, R.F., Ainge, J.A., Couey, J.J., Canto, C.B., Bjerknes, T.L., Witter, M.P., Moser, E.I., and Moser, M.B. (2010). Development of the spatial representation system in the rat. Science *328*, 1576–1580.

Mathis, A., Herz, A.V., and Stemmler, M.B. (2012). Resolution of nested neuronal representations can be exponential in the number of neurons. Phys. Rev. Lett. *109*, 018103.

Mazor, O., and Laurent, G. (2005). Transient dynamics versus fixed points in odor representations by locust antennal lobe projection neurons. Neuron *48*, 661–673.

McInnes, L., Healy, J., and Melville, J. (2018). Umap: uniform manifold approximation and projection for dimension reduction. Preprint at arXiv, 1802.03426.

McNaughton, B.L., Battaglia, F.P., Jensen, O., Moser, E.I., and Moser, M.B. (2006). Path integration and the neural basis of the "cognitive map". Nat. Rev. Neurosci. *7*, 663–678.

Mosheiff, N., Agmon, H., Moriel, A., and Burak, Y. (2017). An efficient coding theory for a dynamic trajectory predicts non-uniform allocation of entorhinal grid cells to modules. PLoS Comput. Biol. *13*, e1005597.

Mosheiff, N., and Burak, Y. (2019). Velocity coupling of grid cell modules enables stable embedding of a low dimensional variable in a high dimensional neural attractor. Elife *8*, e48494.

Moulavi, D., Jaskowiak, P.A., Campello, R.J.G.B., Zimek, A., and Sander, J. (2014). Density-based clustering validation. In Proceedings of the 14th SIAM International Conference on Data Mining (SDM) (SDM Press), pp. 839–847.

Ocko, S.A., Hardcastle, K., Giocomo, L.M., and Ganguli, S. (2018). Emergent elasticity in the neural code for space. Proc. Natl. Acad. Sci. USA *115*, E11798–E11806.

Okun, M., Steinmetz, N.A., Cossell, L., Iacaruso, M.F., Ko, H., Barthó, P., Moore, T., Hofer, S.B., Mrsic-Flogel, T.D., Carandini, M., and Harris, K.D. (2015). Diverse coupling of neurons to populations in sensory cortex. Nature *521*, 511–515.

Pérez-Escobar, J.A., Kornienko, O., Latuske, P., Kohler, L., and Allen, K. (2016). Visual landmarks sharpen grid cell metric and confer context specificity to neurons of the medial entorhinal cortex. Elife *5*, e16937.

Pfeiffer, B.E., and Foster, D.J. (2013). Hippocampal place-cell sequences depict future paths to remembered goals. Nature *497*, 74–79.

Rubin, A., Sheintuch, L., Brande-Eilat, N., Pinchasof, O., Rechavi, Y., Geva, N., and Ziv, Y. (2019). Revealing neural correlates of behavior without behavioral measurements. Nat. Commun. *10*, 4745.

Rybakken, E., Baas, N., and Dunn, B. (2019). Decoding of neural data using cohomological feature extraction. Neural Comput. *31*, 68–93.

Schmitzer-Torbert, N., Jackson, J., Henze, D., Harris, K., and Redish, A.D. (2005). Quantitative measures of cluster quality for use in extracellular recordings. Neuroscience *131*, 1–11.

Seelig, J.D., and Jayaraman, V. (2015). Neural dynamics for landmark orientation and angular path integration. Nature *521*, 186–191.

Skaggs, W.E., McNaughton, B.L., Wilson, M.A., and Barnes, C.A. (1996). Theta phase precession in hippocampal neuronal populations and the compression of temporal sequences. Hippocampus *6*, 149–172.

Sreenivasan, S., and Fiete, I.R. (2011). Grid cells generate an analog error-correcting code for singularly precise neural computation. Nat. Neurosci. *14*, 1330–1337.

Steinmetz, N.A., Aydin, C., Lebedeva, A., Okun, M., Pachitariu, M., Bauza, M., Beau, M., Bhagat, J., Böhm, C., Broux, M., et al. (2021). Neuropixels 2.0: a miniaturized high-density probe for stable, long-term brain recordings. Science *372*, eabf4588.

Stensola, H., Stensola, T., Solstad, T., Frøland, K., Moser, M.B., and Moser, E.I. (2012). The entorhinal grid map is discretized. Nature *492*, 72–78.

Stringer, C., Pachitariu, M., Steinmetz, N., Carandini, M., and Harris, K.D. (2019). High-dimensional geometry of population responses in visual cortex. Nature *571*, 361–365.

Trettel, S.G., Trimper, J.B., Hwaun, E., Fiete, I.R., and Colgin, L.L. (2019). Grid cell co-activity patterns during sleep reflect spatial overlap of grid fields during active behaviors. Nat. Neurosci. *22*, 609–617.

Welinder, P.E., Burak, Y., and Fiete, I.R. (2008). Grid cells: the position code, neural network models of activity, and the problem of learning. Hippocampus *18*, 1283–1300.

Yoon, K., Buice, M.A., Barry, C., Hayman, R., Burgess, N., and Fiete, I.R. (2013). Specific evidence of low-dimensional continuous attractor dynamics in grid cells. Nat. Neurosci. *16*, 1077–1084.

Zong, W., Wu, R., Li, M., Hu, Y., Li, Y., Li, J., Rong, H., Wu, H., Xu, Y., Lu, Y., et al. (2017). Fast high-resolution miniature two-photon microscopy for brain imaging in freely behaving mice. Nat. Methods *14*, 713–719.

CellPress
OPEN ACCESS

STAR★METHODS

KEY RESOURCES TABLE

REAGENT or RESOURCE	SOURCE	IDENTIFIER
Experimental Models: Organisms/Strains		
Rat: Long-Evans	Bred inhouse at KISN	Kavli Institute for Systems Neuroscience
Software and Algorithms		
Matlab 2020a	MathWorks	https://mathworks.com/products/matlab.html RRID: SCR_001622
UMAP	MathWorks	https://www.mathworks.com/matlabcentral/fileexchange/71902
DBSCAN	MathWorks	https://mathworks.com/help/stats/dbscan.html
SpikeGLX	Jun et al., 2017	https://billkarsh.github.io/SpikeGLX/
Kilosort	Steinmetz et al., 2021	https://github.com/MouseLand/Kilosort
Phy	Jun et al., 2017	https://github.com/cortex-lab/phy
DBCV	Moulavi et al., 2014	https://imada.sdu.dk/~zimek/publications/SDM2014/
OptiTrack Motive	OptiTrack	https://optitrack.com/software/motive/
Other		
Neuropixels Probes	Neuropixels	https://www.neuropixels.org/
Neuropixels Control System	Neuropixels	https://www.neuropixels.org/control-system
Kintex-7 FPGA board	Xilinx	https://www.xilinx.com/products/boards-and-kits/device-family/nav-kintex-7.html
Optitrack Flex 13 USB cameras	OptiTrack	https://optitrack.com/cameras/
Zeiss AxioImager	Zeiss	N/A
Deposited Data		
Datasets	This paper	https://doi.org/10.5281/zenodo.6200517

RESOURCE AVAILABILITY

Lead contact

Further information and requests for resources should be directed to and will be fulfilled by the lead contact, Prof. Yoram Burak (yoram.burak@elsc.huji.ac.il).

Materials availability

This study did not generate new unique reagents.

Data and code availability

The data are available at Zenodo (https://doi.org/10.5281/zenodo.6200517).

The code is available at Zenodo (https://doi.org/10.5281/zenodo.6208720).

METHOD DETAILS

Subjects

Experimental testing took place at the Kavli Institute for Systems Neuroscience, NTNU, Norway. Data were obtained from 4 male Long Evans rats (300-500 grams when implanted, at ages P 73-107 days old at day of recording). After weaning at three weeks, the rats were group-housed with their siblings until the implantation date. After implantation, each rat was housed alone in a large two-story enriched metal cage (95 x 63 x 61 cm). The rats were kept in temperature and humidity controlled rooms on a

12 hr light / 12 hr dark schedule. Experiments took place in the dark phase of the schedule. All procedures were performed in accordance with the Norwegian Animal Welfare Act and the European Convention for the Protection of Vertebrate Animals used for Experimental and Other Scientific Purposes.

Electrode implantation surgery

The rats were implanted with single-shank 384-site Neuropixels probes (Jun et al., 2017) targeting the medial entorhinal cortex (MEC) in either one or both hemispheres. Rat #26018 was implanted only in the right hemisphere, while rats #25843 and #26820 were implanted bilaterally with prototype Neuropixels 'phase 3A' probes. Rat #26718 was implanted with a Neuropixels 1.0 probe in the right hemisphere. Before implantation, the rats were anaesthetized with isoflurane in an induction chamber and given subcutaneous injections of buprenorphine (Temgesic) and Meloxicam (Metacam). They were then fixed in a Kopf stereotaxic frame with continuous isoflurane administered through a mask. Local analgesic bupivacaine (Marcaine) was injected subcutaneously before making the incision. Craniotomies were drilled above the MEC area. The probes were inserted at a maximum depth of 5-6 mm from the brain surface, 4.4-4.6 mm lateral to the midline suture, 0.1-0.3 mm anterior to the transverse sinus, at angles between 25-26 degrees from the vertical plane, with the tip of the probe pointing in the anterior direction. A single jewellers screw was secured through the skull above the cerebellum and connected to the probe ground with an insulated silver wire. The implants were secured in place with dental adhesive (Optibond from Kerr) and Venus composite (Kulzer) and protected by fitting a modified falcon tube. Postoperative analgesia (meloxicam and buprenorphine) was administered during the surgical recovery period.

Electrophysiological recordings

Electrophysiological signals were recorded with a Neuropixels acquisition system as described previously (Gardner et al., 2022; Jun et al., 2017). The spike band signal was recorded and amplified with a gain of 500, filtered to keep a bandwidth from 0.3 to 10 kHz and then digitized at 30 kHz on the probe circuit board. The signal was further multiplexed and transmitted to a Xilinx Kintex 7 FPGA board ('phase 3A') or a Neuropixels PXIe acquisition module (1.0) via a 5 m tether cable before being streamed via ethernet connection to a local computer. Rat #26018 had two recording sessions: recording session #26018a was performed 4 days before recording session #26018b, with partial overlap of recorded cells between the two sessions.

Behavioural tracking

During recording, a rigid body with five retroreflective markers was attached to the rat's implant and tracked with a 3D motion capture system (six OptiTrack Flex 13 cameras and Motive software) at ~ 120 Hz. To synchronise the timestamps of the two recording systems, randomized sequences of digital pulses generated by an Arduino microcontroller were sent to both the Neuropixels acquisition system as direct TTL input and to the OptiTrack system via infrared LEDs placed on the edge of the arena.

Behavioural procedures

The rat's movement was tracked as it moved freely in a circular open field arena. The recording arena was a 150 cm diameter, matt black plastic cylinder with 50 cm high walls and a matt black hard rubber floor, surrounded by floor-to-ceiling dark blue blackout curtains on all sides ~ 1m from the arena edge. Three additional layers of blackout curtains separated the recording arena from the part of the room with the recording computer. The same behavioural arena was used in both darkness and light recordings.

Open-field foraging trials in darkness

Complete darkness was ensured by turning off all potential sources of light in the recording room. Light sources which could not be turned off were masked with aluminium foil and electrical tape and/or blackout curtains. Before starting the experiment, the arena and floors were thoroughly cleaned with soap water and dried. The rat's Neuropixels probe was connected to the recording system outside the closed curtains before the final lights were shut off, and the rat was introduced to the recording arena at an arbitrary position and direction. For 50-60 minutes, the rat was left to freely explore the arena and forage small pieces of corn foam snack thrown into the arena during the trial by an experimenter wearing night-vision goggles (Armasight Nyx-7 pro). To avoid delivery of systematic orientational cues, the experimenter accessed and left the ring of curtains from random locations, and quickly dropped food pellets and removed excrement and urine using a paper towel while the rat was in a different location in the arena. Light conditions were not changed during these times.

Open-field foraging task in light

After the open foraging task in darkness, while the rat was still foraging in the arena, or with a short break to untwist the Neuropixels tether cables, the experimenter turned on the light and continued the recording for another 30-60 minutes. During the light task, a single white textile cue card (~ 45 cm wide, ~ 150 cm high) hanging on the blue curtains outside the arena was visible from within the arena. The only light source in the light task was a single LED strip (6 m, 120 LEDs, 2800K color temp) placed as a uniform ~ 2 m diameter ring directly above the arena at a height of ~ 2.8 m, evenly illuminating the arena and ensuring no shadows were cast on the floor.

Perfusion and histology

The rats were anaesthetized with isoflurane in an induction box and given a lethal injection of pentobarbital. When unresponsive, the rats were perfused transcardially with 0.9% saline, followed by 4% Formalin solution. The brain was extracted and stored in 4% Formalin solution for at least 24 hours before being sliced in 30 μm sagittal sections on a cryostat. The brain sections were stained with Cresyl Violet, and photomicrographs were taken through a Zeiss Axio Imager.

Spike sorting and single-unit selection

Spike sorting was performed with a version of KiloSort 2.5 (Steinmetz et al., 2021), optimised for MEC/PaS recordings as described in Gardner et al. (2022), including manual supervision of cluster split and merge processes. Single units were excluded from further analysis if more than 1% of intervals in their interspike interval distribution were shorter than 2 ms or if they had less than 500 total spikes in the light task.

Module classification

Grid cell and module classification was done by vectorizing the spatial autocorrelation from the rate map of every cell and adding them as feature columns in a matrix used as input to the UMAP (Uniform Manifold Approximation and Projection) dimensionality reduction algorithm (McInnes et al., 2018), before DBSCAN (Ester et al., 1996) was used to assign cluster identities to the resulting 2D point clouds, as in Gardner et al. (2022) (Figures 1E, 1F, and S2A–S2C). Briefly, for each recorded cell, rate maps were generated by dividing the arena into 8-10 cm bins and counting the number of spikes within each bin divided by the time spent in that bin. Autocorrelograms of the rate maps were calculated, and values from the bins in a circular area within a 3-bin radius from the center bin were removed along with the bins outside a radius defined by the edge of the matrix. The autocorrelograms were then vectorized and used as features in UMAP used to project the values down to a point cloud in 2 dimensions. DBSCAN was used to cluster the points, which yielded a single large cluster with non-grid cells and single clusters for each module (identified by a clear grid pattern and high gridness score in the mean autocorrelogram of each cluster); grid modules could be ordered by the grid spacing and orientation calculated from the mean autocorrelogram of each cluster. Of all recorded cells, only those from clusters with a clear grid pattern in the mean autocorrelogram, and a validity index (see clustering validation section) close to 1.0 were used for further analysis. Grid pattern classification was defined as follows: for each cluster, we took the mean of the gridness scores of all cells in the cluster. We considered the clusters which had a high within-cluster mean gridness score of order 1, as grid clusters (M1, M2, M3), and clusters with a low within cluster mean gridness score (close to 0) as non-grid. The lowest mean gridness score in a cluster classified as a grid-cell cluster was 0.66 (M2 in session #26718) and the highest mean gridness score in a cluster classified as "non grid-cell" was 0.13 (session #26018b). The mean, SD, and SEM of the gridness scores for each cluster and for all recording sessions are shown in Figure S2F.

Clustering validation

The grid cell and module classification results were validated by calculating a density-based clustering validation (DBCV) index (Moulavi et al., 2014) for each DBSCAN-assigned cluster identity in the 2D UMAP point cloud (Figures 1E, 1F, and S2A–S2C). The DBCV index has a range of -1 to 1, where a cluster gets a positive value if the lowest density region inside the cluster is higher than the highest density in the region that separates it from other clusters (Figures S2G–S2K).

Rate map analysis

The firing rate λ_i [Hz] of grid cell i at each position $\vec{x}$ in the arena was generated as follows:

$$\lambda_i(\vec{x}) = \frac{\sum_{j=1}^{\nu_i} g\left(\vec{x}_i^{\,j} - \vec{x}\right)}{\Delta t \sum_{t=1}^{T} g\left(\vec{y}_t - \vec{x}\right)}$$

where ν_i is the total number of spikes emitted by neuron i, and $\vec{x}_i^{\,j}$ is the animal's position when spike j was emitted. The position of the animal at time t is denoted by $\vec{y}_t$, and g is a two-dimensional Gaussian kernel with diagonal covariance matrix $\sum_{ii} = 25\ \mathrm{cm}^2$. Spike trains and tracking data were binned at $\Delta t = \frac{1}{120}$ s resolution, and only time bins where the animal was moving at a speed greater or equal to 3 $\frac{\mathrm{cm}}{\mathrm{s}}$ were used for spatial analyses.

Gridness score

The gridness score was computed to measure the degree of hexagonal spatial periodicity, as in Langston et al. (2010). For each cell, an autocorrelogram was calculated from its rate map and rotated in five steps of 30 degrees, correlating each rotated matrix with the original autocorrelation in the following manner: First, the values correlated were restricted to a ring of bin indexes around the center peak of the autocorrelogram; then, this ring of bins was expanded stepwise until its outer edge reached the edge of the autocorrelogram matrix. For each step, a score was calculated as the difference between the lowest correlation at [60, 120] degrees and the

highest correlation at [30, 90, 150] degrees. The gridness score was taken as the mean of the three scores surrounding and including the step with the maximum score, resulting in a theoretical score range of [-2, 2].

Information content

The spatial information content [bits/spike] (Skaggs et al., 1996) of neuron i is defined as

$$\sum_{i=1}^{N} p_i \frac{\lambda_i}{\lambda} \log_2\left(\frac{\lambda_i}{\lambda}\right)$$

where λ_i is the unit's mean firing rate in the i-th bin of the rate map, λ is the overall mean firing rate and p_i is the probability of the animal being in the i-th bin (time spent in the i-th bin divided by the duration of recording).

Pairwise correlations

Spike trains were binned at $\Delta t = \frac{1}{120}$ s resolution and smoothed using a Gaussian kernel with $\sigma = 50$ ms. Pearson correlation coefficients were then calculated for pairs of spike trains from simultaneously recorded grid cells.

In Figures 3B and 3D neurons were divided into ten equally sized groups with equal distributions of neurons from each module and cross-correlations were calculated across inter- and intra-module pairs that belong to the same group to obtain independent evaluations. Cross-correlations were calculated by iteratively lagging one of the spike trains relative to the other. To avoid cancellations of positive and negative contributions from intra-modular cell pairs with different phase relationships, we averaged the absolute magnitude of the correlations over pairs within each of the independent groups.

In Figures 3C, 3E, and S4B, the Pearson correlation coefficients were calculated for all possible inter- and intra-module pairs without lagging any of the spike trains.

Markov decoder

The Markov decoder updates its posterior likelihood for position r as follows:

$$p(r;t+\Delta t) = \frac{1}{Z(t)}\left[\int p(r';t)p_D(r|r')\mathrm{d}r'\right]p_S(r;t) \quad \text{(Equation 2)}$$

where $p_D(r|r')$ describes the animal's probability to run from location r' to location r during time interval Δt. The term $p_S(r;t)$ is the probability for all the neurons to emit the observed spikes within the time interval Δt, given the position r. The posterior likelihood is iteratively normalized by $Z(t)$.

Explicitly, $p_D(r|r')$ is a two-dimensional Gaussian distribution centered around position r' with diagonal covariance matrix $\sum_{ii} = 4\ \mathrm{cm}^2$. The time step, $\Delta t = \frac{1}{120}$ s, is equal to the sampling rate of the data. Thus, the diffusion coefficient is $D = 480\ \frac{\mathrm{cm}^2}{\mathrm{s}}$, which was chosen to roughly minimize the MAE in the light recording sessions.

The posterior extracted from spiking activity was evaluated assuming independent Poisson firing, namely

$$p_S(r;t) = \prod_{i=1}^{N} \frac{1}{n_i!}(f_i\Delta t)^{n_i}\exp(-f_i\Delta t)$$

where $f_i(r)$ is the tuning curve and n_i is the spike count of the i'th neuron during the time interval Δt.

Finally, the estimate of position is the maximum likelihood estimate

$$\hat{r} = \underset{r}{\operatorname{argmax}}\, p(r;t)$$

Likelihood of simultaneously recorded spike trains

The probability for observed spike trains $p(\boldsymbol{S}_t)$ is given in Equation 1. By exploiting the Markov decoder's properties, the likelihood for simultaneous observed spikes turns out to be simply proportional to the multiplication of its iterative normalization factors presented above,

$$p(\boldsymbol{S}_t) = \prod_{i=1}^{t} Z_i$$

A detailed analytical derivation is given in Methods S1.

We present in Figures 4 and 5 the average log likelihood per time unit defined as

$$L = \langle \log(Z) \rangle_t \quad \text{(Equation 3)}$$

Since decoding was Markovian we decoded all the data, but only time bins where the animal was moving at a speed greater or equal to 3 $\frac{\mathrm{cm}}{\mathrm{s}}$ were used for further analyses.

Rate-adjusted likelihood

In order to evaluate the likelihood using the Markov decoder and faithfully compare this quantity across light and dark conditions, it is necessary to make concrete assumptions on the neural tuning curves. Since individual grid cells differed in their firing rates in these two conditions, it was necessary to analyze how the firing rate influences the likelihood, and then compensate for this influence. Counter intuitively, the evaluated likelihood decreases as the number of total spikes in the recording increases (see Methods S1 for an analytical derivation which elucidates this finding).

Our assumption is that neurons maintain the same underlying structure of tuning curves in light and dark conditions, up to a scaling factor that adjusts the firing rate (see also Methods S1; Figure S8). Therefore, we matched the mean firing rates by randomly omitting spikes in the condition in which the firing rate was higher, yielding spike trains from each neuron with the same mean firing rate in the dark and light conditions. This was done separately for time bins where the animal was moving at a speed smaller than 3 $\frac{cm}{s}$, and for time bins where the animal was moving at a speed greater or equal to 3 $\frac{cm}{s}$ since these time bins were used for further analysis.

Mean absolute error (MAE)

The MAE is defined as the average Euclidean distance between the decoded position and the true position of the animal.

Spatial shifts

For each module, a spatial shift was chosen at random from the range $[-\alpha, \alpha]$, independently for each of the two spatial dimensions (horizontal and vertical). These shifts were fixed throughout the duration of each simulation. The variable α is plotted as the 'Max spatial shift' axis in the figures throughout this article. Rate maps of all neurons that belong to the same module were shifted according to their corresponding random shifts, thus producing shifted rate maps. Rate maps were set to zero in positions outside of the arena. Firing rates at new positions that were included within the arena boundaries only after the shift but were outside of the arena boundaries before the shift were set to zero. Identical spatial shifts were applied in a similar procedure, but only a single set of two-dimensional shifts were chosen at random and were applied to the rate maps of all neurons as described above, regardless of the module they belong to. Thirty such independent realizations were simulated for each 'Max spatial shift' value.

Rotational Shifts

For each module, a spatial rotation angle was chosen independently and at random from the range $[-\alpha, \alpha]$. These shifts were fixed throughout the duration of each simulation. The variable α is plotted as the 'Max spatial rotation' axis in Figure S6B. Rate maps of all neurons that belong to the same module were rotated with respect to the arena's origin, according to their corresponding random angle, and thus producing rotated rate maps. Identical rotational shifts were applied in a similar procedure, but only a single angle was chosen at random and was used to rotate the rate maps of all the neurons as described above, regardless of the module they belong to. Thirty such independent realizations were simulated for each 'Max rotational shift' value.

Idealized grid cell tuning curves

The idealized grid cells had the same allocation to modules as in the recording sessions (Table 1) mentioned in Figures S4B and S4F captions, with corresponding grid spacings of $\vec{\lambda} = [45, 65, 95]$ cm. Tuning curves were modeled as a sum of Gaussian blobs whose peaks lie on a perfect hexagonal lattice. In each module, the Gaussian blobs had a diagonal covariance matrix $\sum_{ii} = 0.015 \cdot \lambda^2$ cm^2. The peak firing rate was 30 Hz. Phases of cells from the same module were uniformly distributed, and the angular orientation of each module was independently and randomly chosen.

Kernel decoder

The kernel decoder updates its posterior likelihood for position r based on recent emitted spikes, weighted exponentially (Mosheiff et al., 2017). It is straightforward to express the log likelihood of spike counts ν_i, observed within a temporal window of duration Δt, as a function of the position r:

$$\log p(\{\nu_i\}|r) = \sum_i f(r - r_i)\Delta t + \sum_i \nu_i \log\{f(r - r_i)\Delta t\} - \sum_i \nu_i! = c + \sum_i \nu_i \log f(r - r_i) \qquad \text{(Equation 4)}$$

where c is a constant that does not depend on r. The term $\sum_i f(r - r_i)$ contributes only to this constant because of the assumption of dense, transnationally invariant receptive fields with uniform distribution. The index i runs over grid cells: either all the cells to produce the multi module posterior, or on all the cells within a module to produce the corresponding uni-module posterior. A maximum likelihood estimator for r (assuming uniform prior) will chose

$$\hat{r}(\{\nu_i\}) = \underset{r}{\operatorname{argmax}} \sum_i \nu_i \log f(r - r_i).$$

The spikes from recent history are weighted with a temporal kernel $h(t)$. Thus, we generalize ν_i to:

$$\nu_i = \int_{-\infty}^{t} h(t-t')\xi_i(t')\mathrm{d}t'.$$

Here, $\xi_i(t)$ is a series of delta functions that represents the spike from neuron i, and

$$h(t) = \exp\left(-\frac{t}{\tau}\right)$$

where we used $\tau = 100$ ms. In Equation 4 Δt is replaced by τ.

Only time bins where the animal was moving at a speed greater or equal to 3 $\frac{\mathrm{cm}}{\mathrm{s}}$ were used for analyses.

Uni-module decoding

As expected, the posterior was approximately periodic when activity was decoded from single modules. To remove ambiguity, the decoded position $\widehat{u}_i$ of each module i was defined as the position that maximized the posterior within a circular area. The circular area had a diameter equal to $\sim$ 90% of the corresponding module spacing and was centered around the multi-module position $\widehat{m}$ (Figure 6A). Thus, only a single blob was included within the circular area around the multi-module represented position $\widehat{m}$.

Small and large dark error periods

In Figures 7A, 7C, 7E, and 7G the error between the joint kernel decoded position and true position during darkness was first temporally smoothed using a Gaussian kernel with $\sigma = 50$ ms. Periods with particularly small error are defined as continuous non-overlapping segments spanning at least 1 s with a maximal smoothed error (SE) of 10 cm. Periods with particularly large error are defined as continuous non-overlapping segments spanning at least 1 s with a minimal SE of 20 cm, and a maximal SE which was determined as follows: the maximal SE was chosen as the value corresponding to 80% of the cumulative distribution of the error between the joint kernel decoded position and true position. Time points with errors larger than this cutoff were discarded from the analysis in accordance with the cutoff used in Figures 7B, 7D, 7F, and 7H (largest MAE value shown in legend) due to sparseness of the joint distribution with the distance between $\widehat{u}_i$ and $\widehat{m}$ (= δ_i). Recording session #25843 had 257 large-error segments and 305 small-error segments. Recording session #26018a had 426 large-error segments and 126 small-error segments. Recording session #26018b had 460 large-error segments and 156 small-error segments. Recording session #26820 had 384 large-error segments and 92 small-error segments.

Null hypotheses of MAEs and δ_is

The null hypothesis for the MAE was evaluated for the light and dark trajectories in each recording session (as specified in Figures 5 and S6A captions). It is defined as the mean of Euclidean distance between a randomly chosen position and the true position of the animal in the arena. Thirty realizations have been simulated for each recording session and illumination condition, yielding SEMs in the order of 10^{-2} cm.

The null hypotheses for the δ_i s (as specified in Figures 6 and S7 captions) are defined similarly, but the distances were calculated between the multi-module decoded position and a randomly chosen position within the corresponding module's circular area as defined above (*Uni-module decoding*). Thirty realizations have been simulated for each recording session and illumination condition, yielding SEMs in the order of 10^{-3} cm.

SEM of correlated time series

Whenever the standard error of the mean (SEM) of a single time series signal (S) was evaluated, correlations were taken into account by updating the signal's variance based on the auto-correlation function $(ACF(S))$.

For an independent signal the variance is simply ACF_0. However, for a temporally correlated stationary signal, the actual variance is written as

$$\mathrm{Var}(S) = ACF_0 + \sum_{i=1}^{\infty} 2 \cdot ACF_i$$

where in practice the cutoff point of the sum remains to be determined. We trimmed the sum at a point corresponding to an auto-correlation value satisfying $ACF_i \leq 0.15 \cdot ACF_0$. Finally, the SEM is defined as $\mathrm{SEM} = \sqrt{\mathrm{Var}(S)/n}$ where n is the total number of points in the signal.

Neuron, Volume *110*

Supplemental information

Grid-cell modules remain coordinated when neural activity is dissociated from external sensory cues

Torgeir Waaga, Haggai Agmon, Valentin A. Normand, Anne Nagelhus, Richard J. Gardner, May-Britt Moser, Edvard I. Moser, and Yoram Burak

Supplemental Information

Grid-cell modules remain coordinated when neural activity is dissociated from external sensory cues

Torgeir Waaga[†,1], Haggai Agmon[†,2,4], Valentin A. Normand[1], Anne Nagelhus[1], Richard J. Gardner[1], May-Britt Moser[1,5], Edvard I. Moser[1,4,5] & Yoram Burak[2,3,4,5,6]

[1] Kavli Institute for Systems Neuroscience and Centre for Neural Computation, Norwegian University of Science and Technology, Trondheim, Norway.

[2] Edmond and Lily Safra Center for Brain Sciences, The Hebrew University of Jerusalem, Jerusalem, Israel.

[3] Racah Institute of Physics, The Hebrew University of Jerusalem, Jerusalem, Israel.

[4] Corresponding author (haggai.agmon@mail.huji.ac.il, edvard.moser@ntnu.no, yoram.burak@elsc.huji.ac.il).

[5] Senior author.

[6] Lead contact.

[†] These authors contributed equally.

Supplementary Figures:

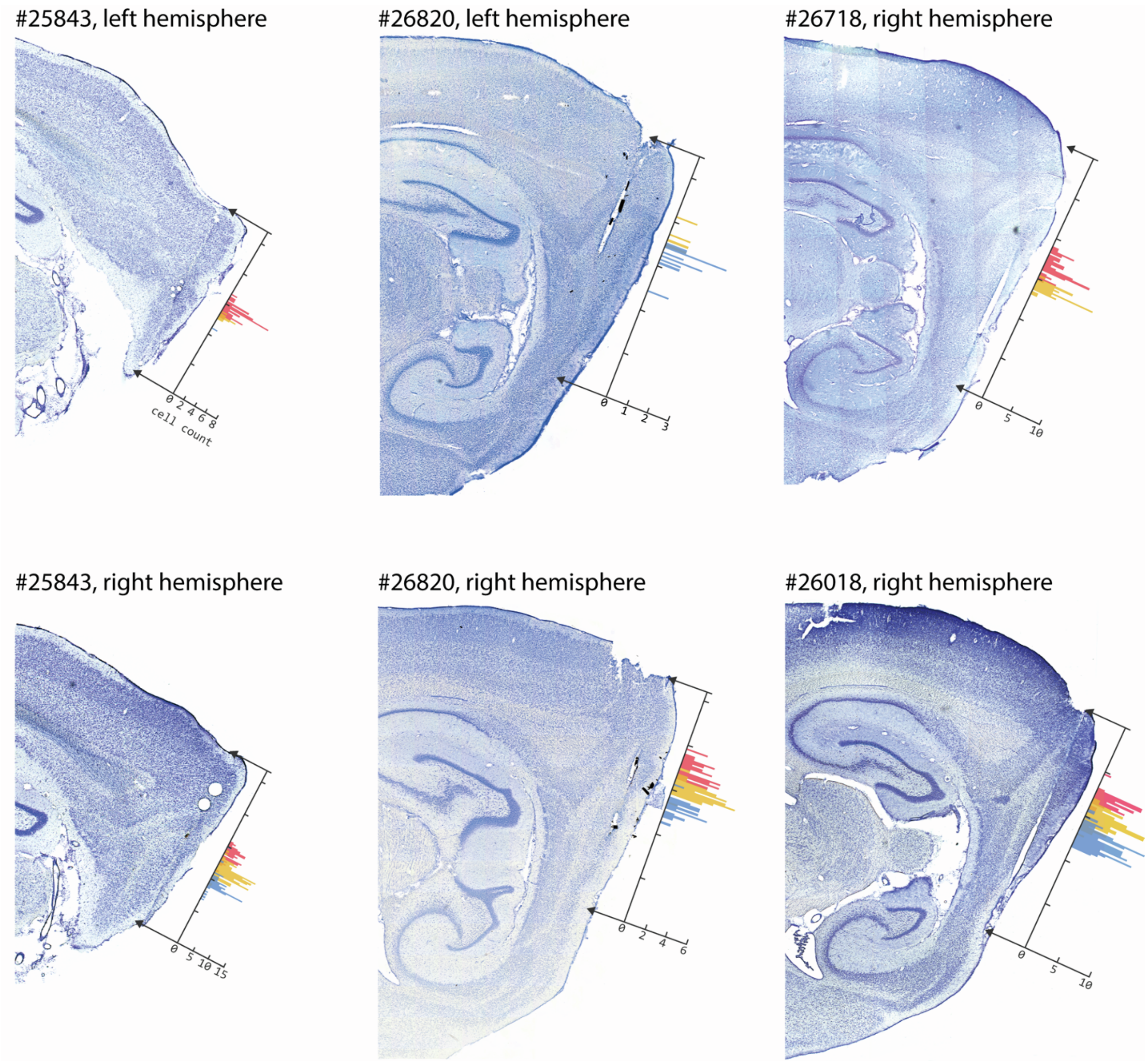

Figure S1. Histology and estimated recording sites. Related to Figure 1.
One cresyl violet-stained sagittal section is shown for each rat's neuropixels probe, showing the probe track left in the brain tissue. Estimated entering sites in the brain as well as probe tip locations are marked with arrows. The histogram shows the grid cell count across dorso-ventral recording depths from different modules (color coded). The distance between two adjacent ticks along the probe shank axis corresponds to 1 mm.

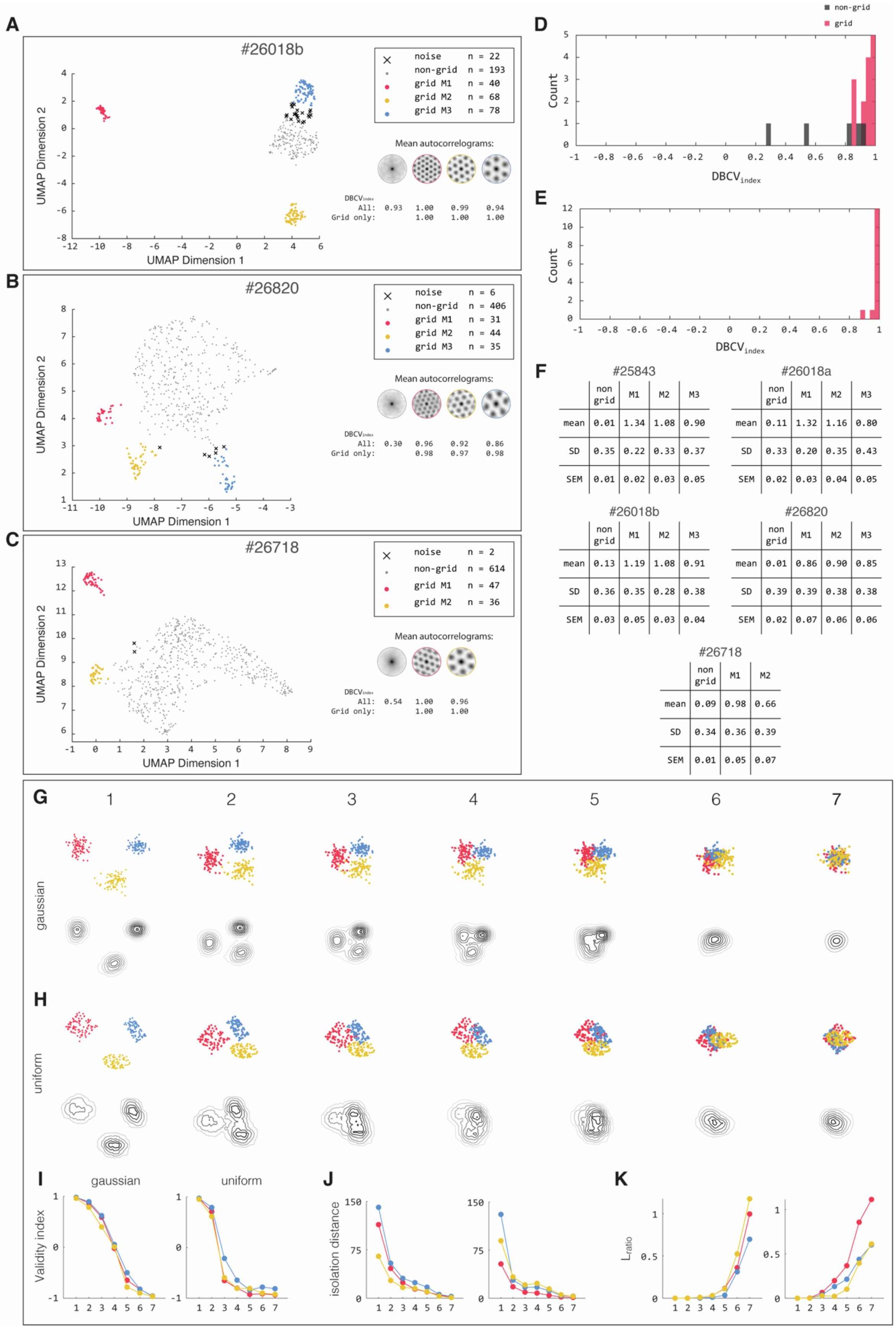

#25843

	non grid	M1	M2	M3
mean	0.01	1.34	1.08	0.90
SD	0.35	0.22	0.33	0.37
SEM	0.01	0.02	0.03	0.05

#26018a

	non grid	M1	M2	M3
mean	0.11	1.32	1.16	0.80
SD	0.33	0.20	0.35	0.43
SEM	0.02	0.03	0.04	0.05

#26018b

	non grid	M1	M2	M3
mean	0.13	1.19	1.08	0.91
SD	0.36	0.35	0.28	0.38
SEM	0.03	0.05	0.03	0.04

#26820

	non grid	M1	M2	M3
mean	0.01	0.86	0.90	0.85
SD	0.39	0.39	0.38	0.38
SEM	0.02	0.07	0.06	0.06

#26718

	non grid	M1	M2
mean	0.09	0.98	0.66
SD	0.34	0.36	0.39
SEM	0.01	0.05	0.07

Figure S2. Grid cell classification by UMAP-DBSCAN. Related to Figure 1.
A-C, Same as Figure 1E-F but for three additional recording sessions. **D,** Distribution of validity index with non-grid cluster included for all rats and recordings. **E,** Distribution of validity index with non-grid cluster excluded for all rats and recordings. **F,** Tables of gridness scores (mean, SD, and SEM) for clusters corresponding to grid modules as well as non-grid clusters (UMAP-DBSCAN clusters for all recording sessions). **G-K,** Clustering quality validation: examples of DBCV index on varying clustering quality. Synthetic data points in clusters with varying degree of separation, either random two-dimensional gaussian distributions (G) or random uniform distributions with different shapes (H). All plots have the same scale. **G-H,** Top: synthetic data scatterplot with color coded cluster assignment. Bottom: contour plots illustrate the density. **I,** Density based clustering validity (DBCV) index of the clusters in (G, left) and (H, right), with color corresponding to cluster. Well separated clusters have a DBCV index above zero. **J-K,** We include the isolation distance and L-Ratio measures (Schmitzer-Torbert et al., 2005) commonly used for comparison of clusters of tetrode-recorded spikes from the hippocampus. The two measures were developed for measuring clustering quality for spike sorting tetrode data and assume the clusters form a gaussian distribution in feature space; they might not be ideal for quantifying density-based clustering results in UMAP space.

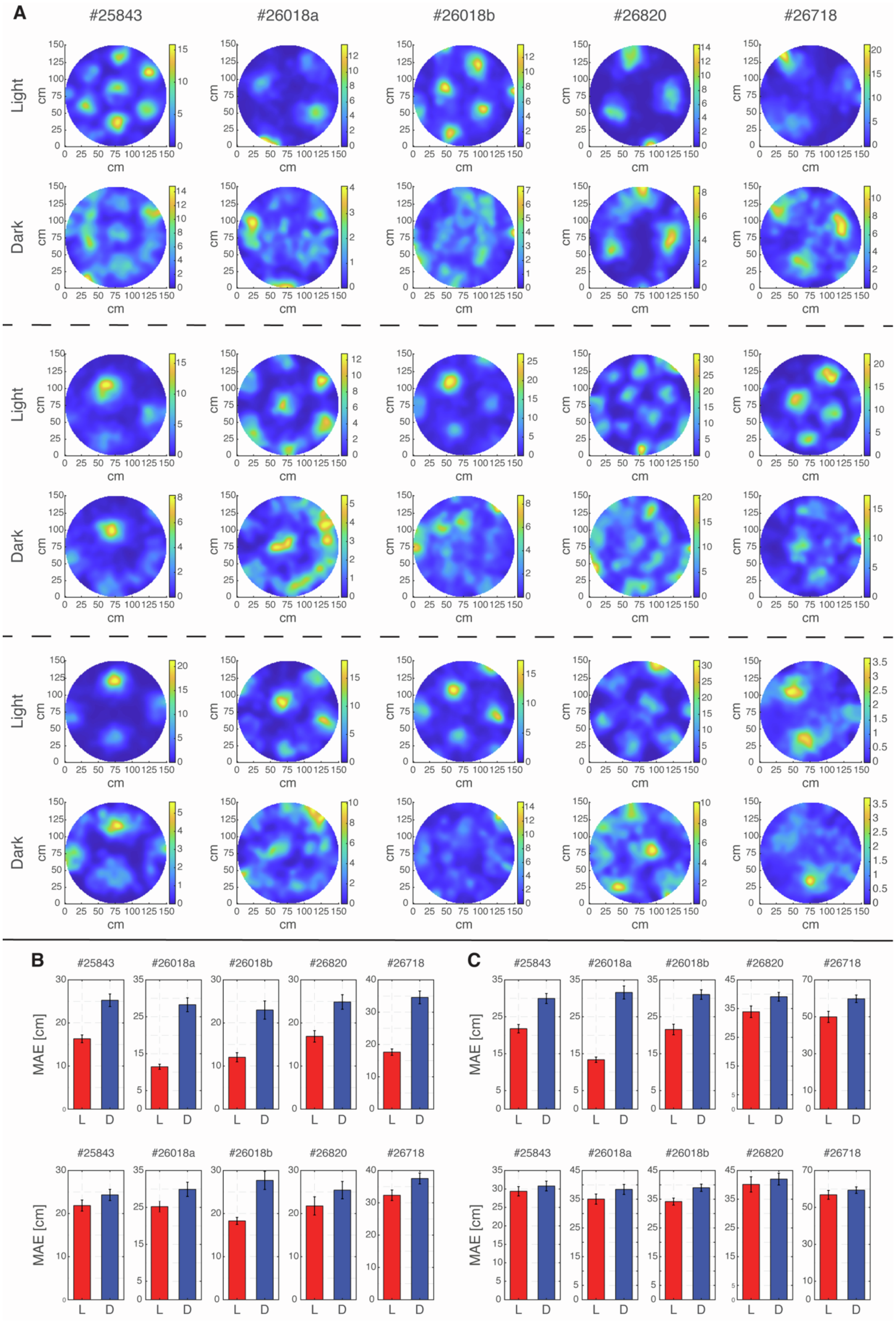
A
#25843
#26018a
#26018b
#26820
#26718
Light
Dark
cm
B
C
MAE [cm]
L
D

Figure S3. Additional individual grid cell rate maps examples from light and dark and unimproved decoding performance even when using dark generated rate maps. Related to Figure 2.
A, Same as Figure 2A, but for another three examples from each recording session. **B,** Top: Mean Absolute Error (MAE) of the Markov decoder applied on the light and dark recorded spiking activity for all cells from single recording sessions and when rate maps were constructed only from one half of the light data (and used to decode the other half of data). Bottom: same as top, but when rate maps were constructed only from one half of the dark data (and used to decode the other half of data). As expected, using the dark-generated rate maps leads to an increase of the MAE both in light and dark conditions, relative to decoding using light-generated rate maps. Importantly, the MAE of decoded position in the dark recording sessions is still larger than in light sessions, even when dark-generated maps are used. Error bars are ±SEM. **C,** Same as (B) but for the kernel decoder.

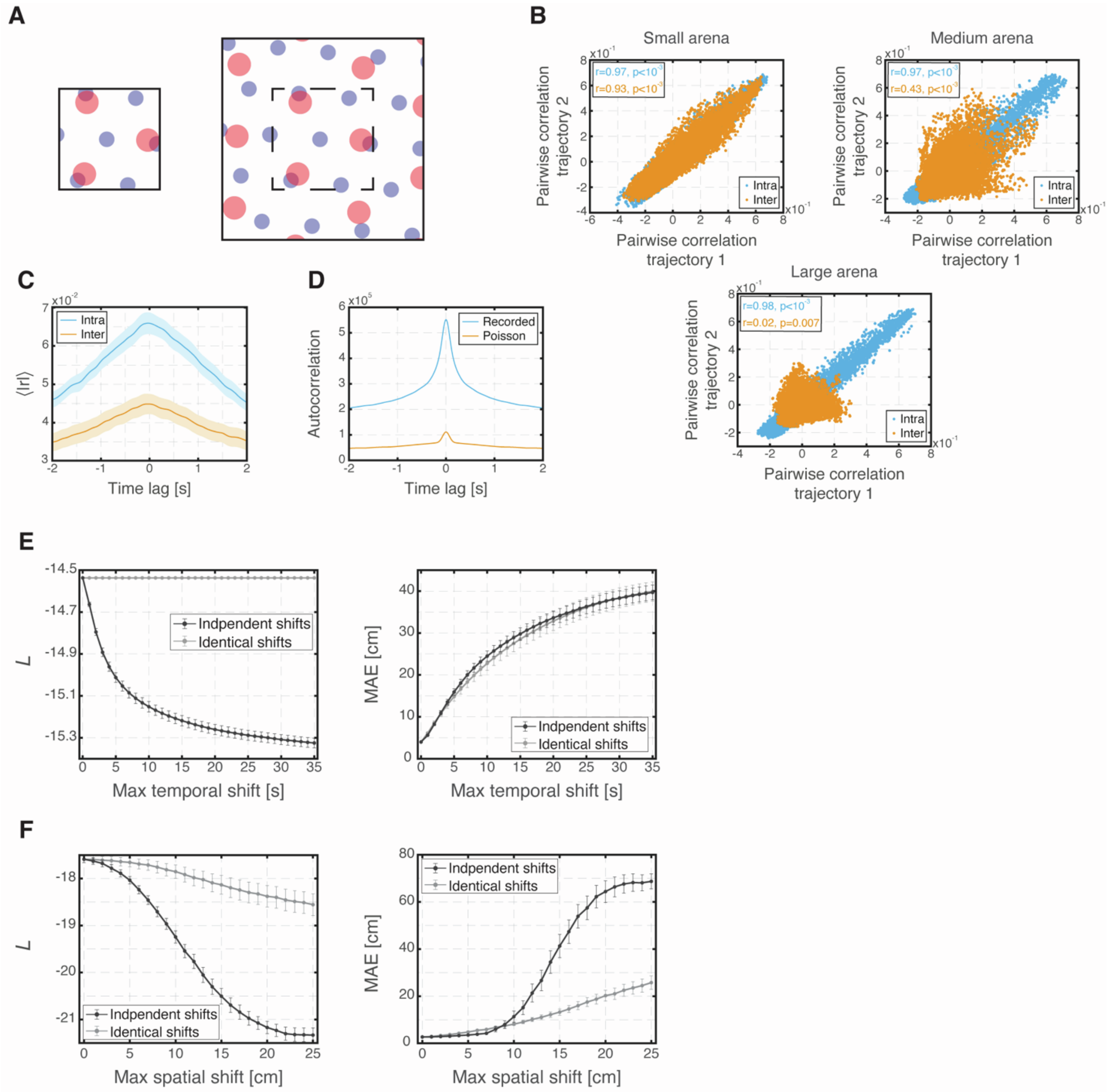

Figure S4. Additional pairwise correlation analyses and controls of the likelihood approach using simulated data. Related to Figure 3 and Figure 4.

A-B, *Inter-module pairwise correlations diminish with increased environment Size.* **A,** Schematic illustration showing spatial tuning curves of two inter-module grid cells (blue and red). The mean correlation between the tuning curves is higher when the environment is small (left) than when it is large (right). In larger environments, the spike rate correlations are expected to be more narrowly distributed around Zero. **B,** Pairwise correlations of all possible intra- (cyan) and inter- (orange) module pairs from two simulated trajectories in small (radius=30 cm), medium (radius=60 cm) and large (radius=150 cm) circular arenas. Correlation coefficients and p-values are specified in the insets. As expected, inter-module spike rate correlations became narrowly distributed around zero with an increase in the size of the environment, whereas intra-module spike rate correlations remained unaffected. Simulated grid cells share the spacings and module allocation as recorded in session #26018a and emit Poisson spikes which

are determined by their idealized tuning curves (*Methods*). **C-D,** *Sources of variability other than spatial selectivity, contribute to the spike rate correlations.* **C,** Absolute cross-correlation (Pearson coefficient) of inter- and intra-module spiking activities, averaged over cell pairs, obtained from Poisson spike trains which were simulated in response to the light trajectory from recording session #26018a. Shaded error bars are ±SEM. Note that the spatial selectivity on its own is predicted to generate cross correlations that decay slowly in time, but there are notable differences when compared with the actual data. First, the temporal structure of the decay predicted by the Poisson model differs from the structure observed in the data (Figure 3B). Second, the Poisson model does not fully explain the magnitude of cross correlations at the zero-lag (Figure 3C). **D,** Temporal autocorrelation of the population activity from the light trial in recording session #26018a (blue trace). The population activity signal was evaluated as a sum over all cells of their temporally smoothed spiking activity. The orange trace shows the predicted temporal autocorrelation obtained from Poisson spike trains which were simulated in response to the same trajectory. As expected, the autocorrelation predicted by the Poisson model is weak, because the summed firing rate of all the neurons is only weakly modulated by the position of the animal, and in the Poisson model this is the only determinant of the cell activity. The difference between the traces suggests that network-wide fluctuations in the activity of cells, of non-spatial origin, contribute to the correlations in addition to the spatial selectivity, but note that much of the zero-lag correlations is well predicted by the Poisson model (Figure 3C). **E,** *Likelihood slightly decreases under identical spatial shifts due to boundary conditions.* In order to demonstrate that the slight decrease in the likelihood when using identical spatial shifts is due to boundary conditions, temporal shifts were applied to the same data as in Figure 4B-C. Independent temporal shifts were applied by shifting the timing of spike trains of all neurons that belong to the same module, and identical temporal shifts were applied in a similar fashion but identically for all neurons regardless of the module they belong to. Rate maps were unaffected during this procedure thus their boundaries were not trimmed as in the spatial shift procedure (see also Figure S6B for a similar result using rotational shifts). **Left:** Likelihood of simulated Poisson spikes using measured rate maps and recorded light trajectory from session #26018b for varying magnitudes of temporal shifts. When shifts are applied independently for each module, the likelihood decreases significantly while remaining precisely fixed when temporal shifts are identical. Error bars are ±SEM. **Right:** The Mean Absolute Error (MAE) increases significantly both for independent and for identical temporal shifts as their magnitude increases. Error bars are ±SEM. **F,** *Applying spatial shifts during spike generation is equivalent to spatial shift implementation during decoding.* Similar analysis as in Figure 4. Instead of applying spatial shifts to the rate maps in the decoding process, Poisson spike trains were generated using idealized grid cell tuning curves (*Methods*) under varying magnitudes of spatial shifts that were applied directly to the position encoded by each module. This represents more closely the type of shifts that may occur in the recorded data. Note, however, that it is impossible to apply spatial shifts directly to the recorded spike trains. The goal of these simulations is to demonstrate that the two procedures yield similar outcomes. **Left:** Likelihood of simulated Poisson spikes using idealized grid cell tuning curves and recorded light trajectory from recording session #26018b, evaluated versus varying magnitudes of spatial shifts which were applied to the rate maps during the simulated spike generation. Compare with Figure 4B (values of the likelihood differ because of the idealized tuning curves used here, vs. the measured rate maps used in Figure 4B-C). Error bars are ±SEM. **Right:** The corresponding MAE of the decoder. The MAE increases significantly both for independent and for identical spatial shifts as their magnitude increases. It increases more rapidly for independent shifts due to global decoding errors. This effect is stronger than in Figure 4C due to the use of idealized tuning curves, which allows to complete the tuning curves outside the extent of the arena after applying the shift, whereas in Figure 4C rate maps were completed to zero in regions that were outside of the arena boundaries before the shift (*Methods*). Error bars are ±SEM.

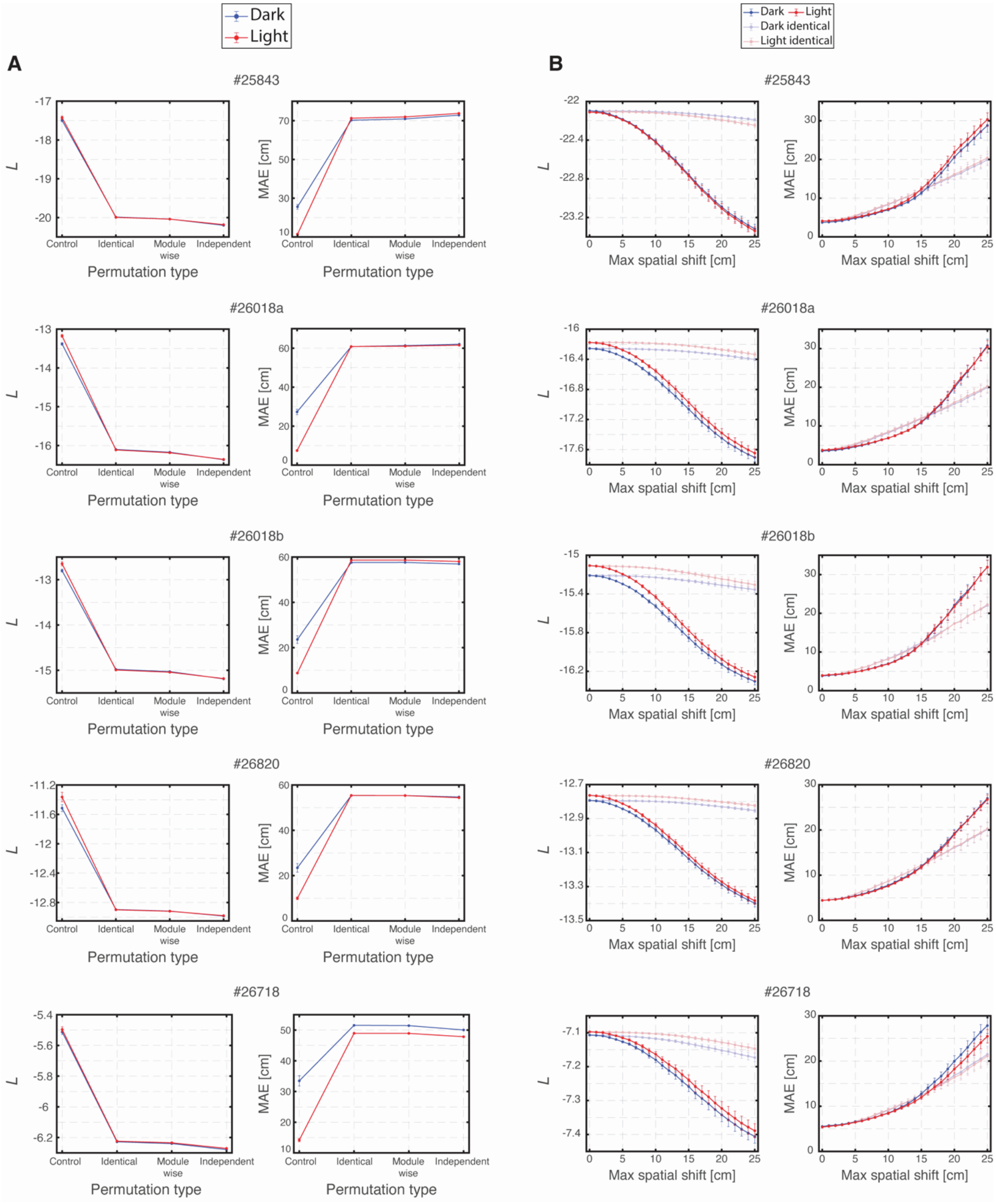

Figure S5. Additional controls of the likelihood approach using recorded data. Related to Figure 5.
A, *The temporal structure of simultaneously recorded spike trains, and not simply their mean firing rate, is necessary to account for the likelihood and MAE results.*

Three types of permutations, which preserve the mean firing rates, were applied to the simultaneous recorded spike trains. 'Identical' describes a permutation type in which the exact permutation was applied to all neurons, 'Module-wise' describes a permutation type in which identical permutations were applied but only to neurons that belong to the same module, and 'Independent' describes a permutation type in which an independent permutation was applied to each neuron. Likelihood of simultaneous recorded spike trains (left column) and corresponding Mean Absolute Error (MAE, right column) of dark and light trials from all recording sessions are shown for the different permutation types. Error bars are ±SEM.
B, *Motion statistics differences between the light and dark trajectories are not expected to substantially affect the likelihood.*
Simulated Poisson spikes were generated for trajectories taken either from the light or dark trials. Rate maps and all other parameters were identical in the two sets of simulations. Rate adjusted likelihood (left column) and Mean Absolute Error (MAE, right column) using the light and dark trajectories for all recording sessions are shown for varying magnitudes of independent module-wise spatial shifts. The differences between the zero-shift light and dark likelihoods are smaller than their corresponding differences shown in Figure 5B-C and Figure S6A, indicating that the difference in motion statistics between light and dark trajectories does not substantially affect the likelihood. As expected, the MAE is nearly identical for both light and dark trajectories. Identical spatial shifts in all modules are superimposed using faded colors for reference. Error bars are ±SEM.

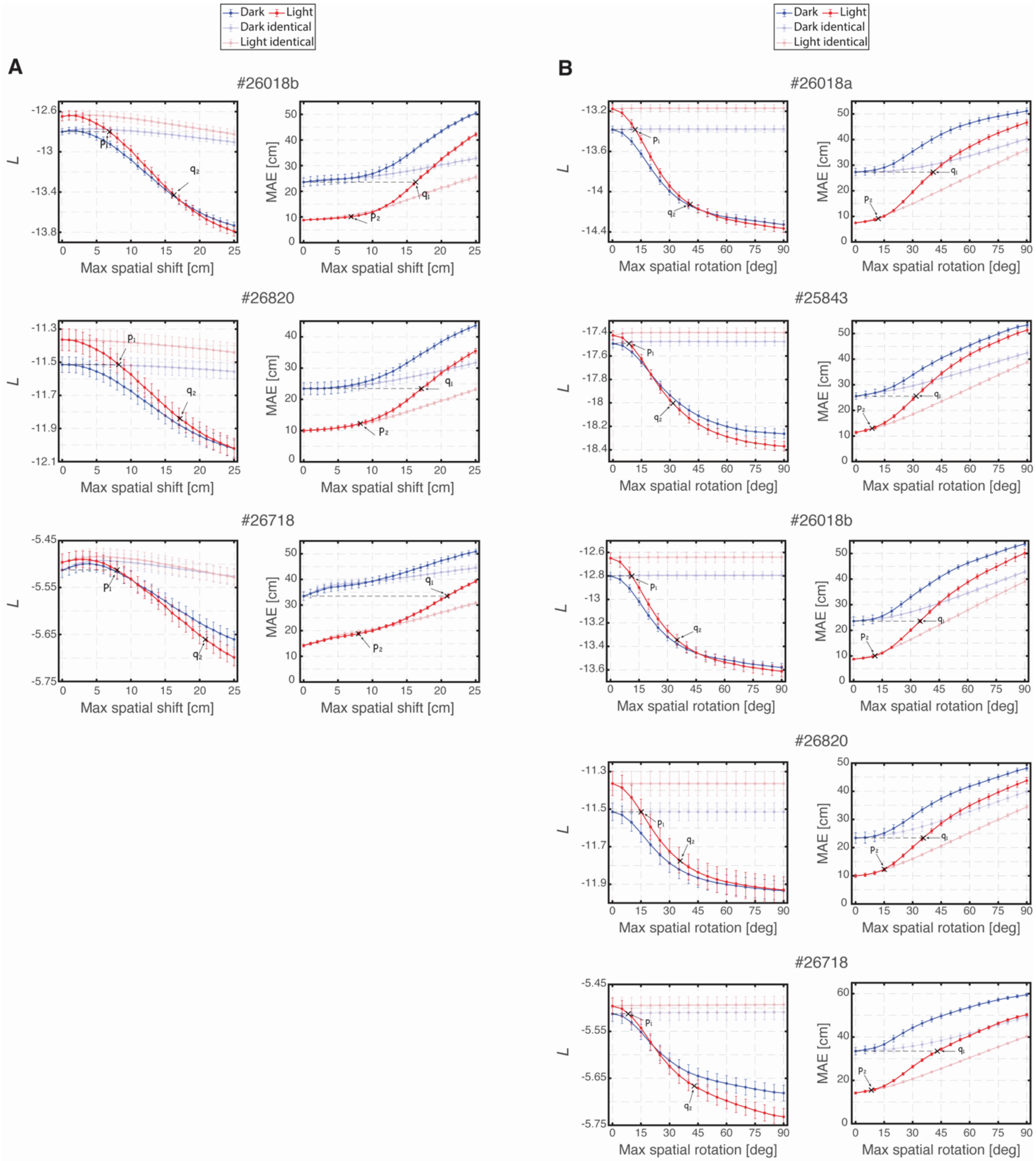

Figure S6. Additional recorded data analyses using the likelihood-based approach. Related to Figure 5.
A, Same as Figure 5C, for three additional recording sessions. The Mean Absolute Errors (MAEs) under the null hypotheses (*Methods*) for the light and dark trials are ~71 cm and ~70 cm correspondingly in recording session #26018b, ~66 cm and ~66 cm respectively in recording session #26820, and ~70 cm and ~72 cm respectively in recording session #26718. Error bars are ±SEM.
B, *Analysis of simultaneously recorded spike trains using rotational shifts.*

Same as Figure 5 but with application of rotational shifts (*Methods*) instead of spatial shifts. Likelihood of simultaneously recorded spike trains (left column) and corresponding Mean Absolute Error (MAE, right column) of dark and light trials from all recording sessions are shown for varying magnitudes of independent module-wise rotational shifts. Recording session #26018a results are explained as an example (top panels): applying a maximal spatial rotation of 12 degrees in the #26018a light recording achieves the same likelihood as that of the dark recording with zero spatial rotation (point p_1, top left panel), but generates only a slight increase of less than 2 cm in the corresponding MAE of the light recording relative to its zero spatial rotation value (point p_2, top right panel). Conversely, applying a maximal spatial rotation of 41 degrees in the #26018a light recording achieves the same MAE as that of the dark recording with zero spatial rotation (point q_1, top right panel), but generates a dramatic decrease in the likelihood (point q_2, top left panel). The difference in the likelihood between point q_2 and the zero spatial rotation point of the dark recording is much larger than the difference between the likelihood values of light and dark zero spatial rotation points. Identical rotational shifts in all modules are superimposed using faded colors. Under identical rotational shifts the dark and light likelihoods are completely unaffected (left) while the corresponding MAE increases significantly in both cases (right). Error bars are ±SEM.

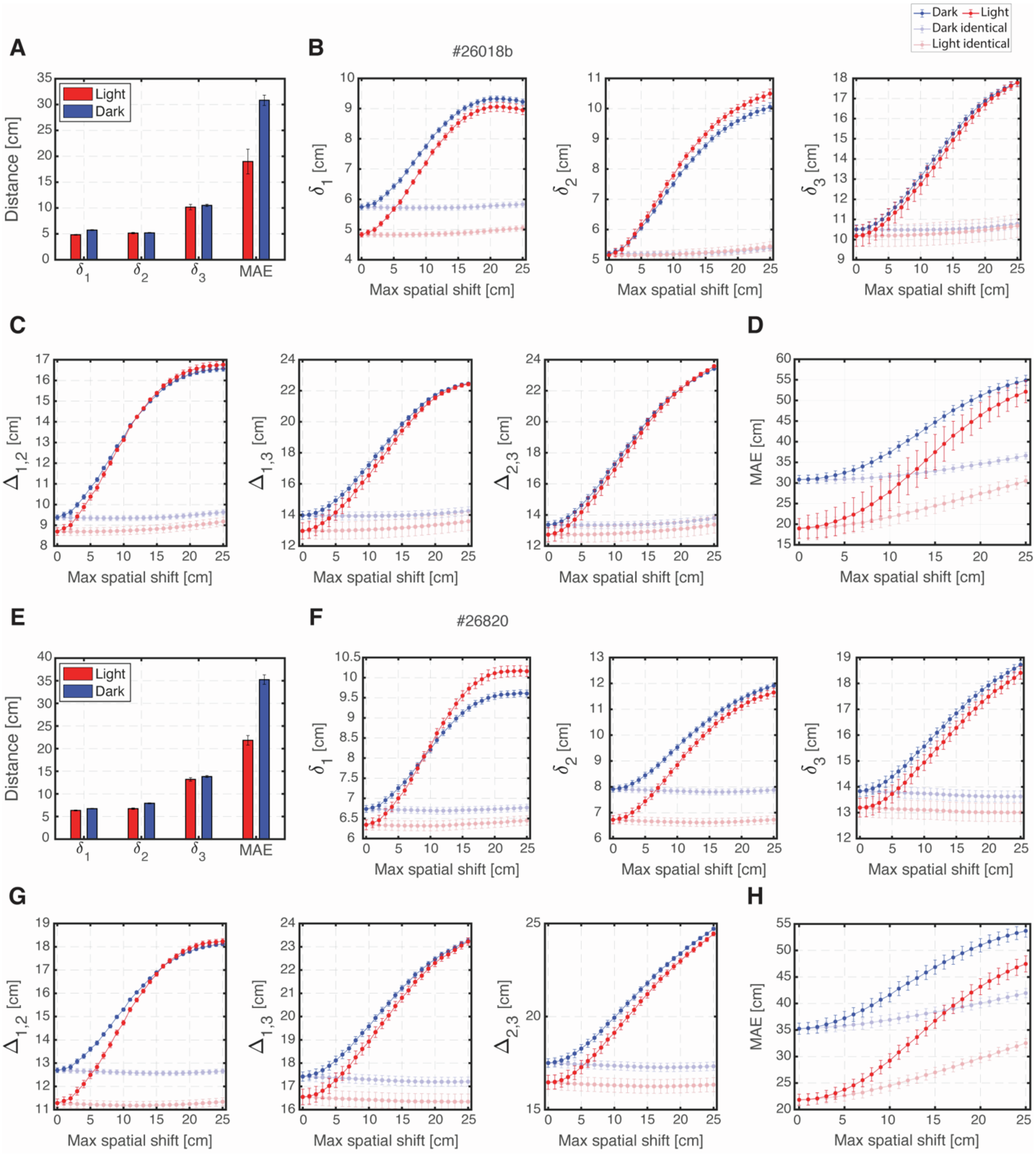

Figure S7. Results for additional datasets using the uni-module decoding approach. Related to Figure 6. A-D and **E-H,** Same as Figure 6B-E for two additional recording sessions (#26018b and #26820) in which recordings were obtained from three modules. Note that even though recordings from three modules were available in these datasets, the numbers of simultaneously recorded grid cells were relatively small (especially in recording session #26820; Table 1), leading to inaccurate decoding. The δ_is under the null hypotheses (*Methods*) for the light and dark trials are ~[13.7,18.2,25.7] cm and ~[13.8,18.2,25.6] cm respectively in (A), and ~[14.4,18.2,27.3] cm and ~[14.4,18.2,27.3] cm respectively in (E). Error bars are ±SEM.

Methods S1

Analytic derivations of the likelihood-based approach. Related to Figure 4, Figure 5, and STAR Methods.

a. Likelihood of simultaneously recorded spike trains

In this subsection we mathematically derive the likelihood of simultaneously recorded spike trains, independently from the animal's true position. To address the inter-module coordination question, we sought to derive a measurement which can quantify the coherence of the simultaneously recorded spike trains even if the represented position in the brain is dissociated from the animal's true position.

Denote by $\boldsymbol{X}_t \equiv \{\vec{x}_0, \vec{x}_1, \ldots, \vec{x}_t\}$ a particular realizable two-dimensional trajectory up to time t, and by $p(\boldsymbol{X}_t)$ the probability for the trajectory $\boldsymbol{X}_t$ to be realized. Since the prior on the trajectory is Markovian, $p(\boldsymbol{X}_t)$ satisfies

$$p(\boldsymbol{X}_t) = p(\vec{x}_t|\vec{x}_{t-1}) \cdot p(\vec{x}_{t-1}\,|\vec{x}_{t-2}) \cdots p(\vec{x}_1|\vec{x}_0) \cdot p(\vec{x}_0) \tag{S1}$$

where $p(\vec{x}_0)$ is the probability of the initial position.

The probability to observe the simultaneous recorded spike trains, averaged over all possible trajectories up to time t, is written as

$$p(\boldsymbol{S}_t) = \sum_{\boldsymbol{X}_t} p(\boldsymbol{X}_t) \cdot p(\boldsymbol{S}_t|\boldsymbol{X}_t) \tag{S2}$$

where the sum is over all possible trajectories, weighted by their corresponding priors $p(\boldsymbol{X}_t)$.
Extending Eq. S2 for the consecutive time step $t+1$,

$$p(\boldsymbol{S}_{t+1}) = \sum_{\boldsymbol{X}_{t+1}} p(\boldsymbol{X}_{t+1}) \cdot p(\boldsymbol{S}_{t+1}|\boldsymbol{X}_{t+1}) \tag{S3}$$

Due to the Markov properties $p(\boldsymbol{X}_{t+1}) = p(\vec{x}_{t+1}|\vec{x}_t) \cdot p(\boldsymbol{X}_t)$, and due to the relationship $p(\boldsymbol{S}_{t+1}|\boldsymbol{X}_{t+1}) = p(\boldsymbol{S}_t|\boldsymbol{X}_t) \cdot p(s_{t+1}|\vec{x}_{t+1})$, Eq.S3 can be written as follows:

$$p(\boldsymbol{S}_{t+1}) = \sum_{\boldsymbol{X}_t} \sum_{x_{t+1}} p(\boldsymbol{X}_t) \cdot p(\boldsymbol{S}_t|\boldsymbol{X}_t) \cdot p(s_{t+1}|\vec{x}_{t+1}) \cdot p(\vec{x}_{t+1}|\vec{x}_t) \tag{S4}$$

Using Bayes law and rearranging yields

$$p(\boldsymbol{S}_{t+1}) = p(\boldsymbol{S}_t) \cdot \sum_{\boldsymbol{X}_t} \sum_{\vec{x}_{t+1}} p(\boldsymbol{X}_t|\boldsymbol{S}_t) \cdot p(s_{t+1}|\vec{x}_{t+1}) \cdot p(\vec{x}_{t+1}|\vec{x}_t) \tag{S5}$$

Re-writing the Markov decoder from the *Methods* (Eq. 2) using this notation,

(S6)

$$p(x_{t+1}|\boldsymbol{S}_{t+1}) = \frac{1}{Z_{t+1}} \cdot \sum_{\boldsymbol{X}_t} [p(\boldsymbol{X}_t|\boldsymbol{S}_t) \cdot p(\vec{x}_{t+1}|\vec{x}_t)] \cdot p(s_{t+1}|\vec{x}_{t+1})$$

where the normalization factor Z_{t+1} satisfies the demand

(S7)

$$\sum_{\vec{x}_{t+1}} p(\vec{x}_{t+1}|\boldsymbol{S}_{t+1}) \overset{!}{=} 1$$

Summing over $\sum_{\vec{x}_{t+1}}$ on both sides of Eq. S6 and rearranging we obtain

(S8)

$$Z_{t+1} = \sum_{\boldsymbol{X}_t} \sum_{\vec{x}_{t+1}} p(\boldsymbol{X}_t|\boldsymbol{S}_t) \cdot p(\vec{x}_{t+1}|\vec{x}_t) \cdot p(s_{t+1}|\vec{x}_{t+1})$$

which is identical to the expression in Eq. S5. Thus, and in recursion,

(S9)

$$p(\boldsymbol{S}_{t+1}) = p(\boldsymbol{S}_t) \cdot Z_{t+1} = p(\boldsymbol{S}_{t-1}) \cdot Z_t Z_{t+1} = \cdots = p(\boldsymbol{S}_{t_0}) \cdot \prod_{i=1}^{t+1} Z_i$$

Finally, applying natural logarithm to Eq. S9 while neglecting the border term $p(\boldsymbol{S}_{t_0})$, which is justified once sufficient iterations have been made, yields

(S10)

$$\log[p(\boldsymbol{S}_t)] = \sum_{i=1}^{t} \log(Z_i)$$

Qualitatively, the likelihood is accrued in a linear fashion over time, indicating that $\log(Z)$ was drawn from an approximately stationary distribution within each recording session (Figure S8A). Therefore, to compare results between light and dark trials, we define the likelihood of simultaneous recorded spike trains as the mean of $\log(Z)$ over time, which can be thought as the amount of likelihood per time unit,

(S11)

$$L \equiv \langle \log(Z) \rangle_t$$

b. *Rate-adjusted likelihood*

In this subsection we elucidate analytically why the posterior likelihood depends on the mean firing rate, and counter-intuitively decreases with increased spiking activity.

Denote by ξ a series of time binned spike trains which can take only $\{0,1\}$ values in each time bin Δt. Under the assumption of Poisson firing and in the limit of small Δt, the posterior likelihood is written as

(S12)

$$\xi_i \cdot \log[\lambda(x_i)\Delta t] + (1 - \xi_i) \log[1 - \lambda(x_i)\Delta t] \cong -\int \lambda(x_t) \mathrm{d}t + \sum_{i=1}^{T} \xi_i \cdot [\log[\lambda(x_i)\Delta t] + \lambda(x_i)\Delta t]$$

Since $0 < \lambda(x_i)\Delta t \ll 1, \forall\, i$, we conclude (counter intuitively) that the addition of each spike can only decrease the posterior likelihood. This can be interpreted as arising from the fact that in the limit of small

Δt, the update of the likelihood with the addition of each spike is proportional to the probability that this particular spike will fall exactly within the relevant time bin.

Thus, to faithfully compare likelihoods between light and dark trials, it was essential to take into account mean firing rates modifications. The underlying assumption in the likelihood approach is that emitted spikes in darkness were generated from the same tuning curves as observed in light up to a multiplicative scaling factor. We down-sampled spike trains by randomly omitting spikes until the mean firing rates matched between the two trials, thus producing rate-adjusted spike trains. To demonstrate that the likelihood indeed depends on the mean firing rate and to justify that such random down sampling is appropriate, we varied a multiplicative factor which controlled the firing rate which accounts for the generation of simulated Poisson spike trains. As expected, the evaluated likelihood decreased as the mean firing rate increased but was completely unaffected if spike trains were subsequently down-sampled randomly using the corresponding multiplicative factor (Figure S8B).

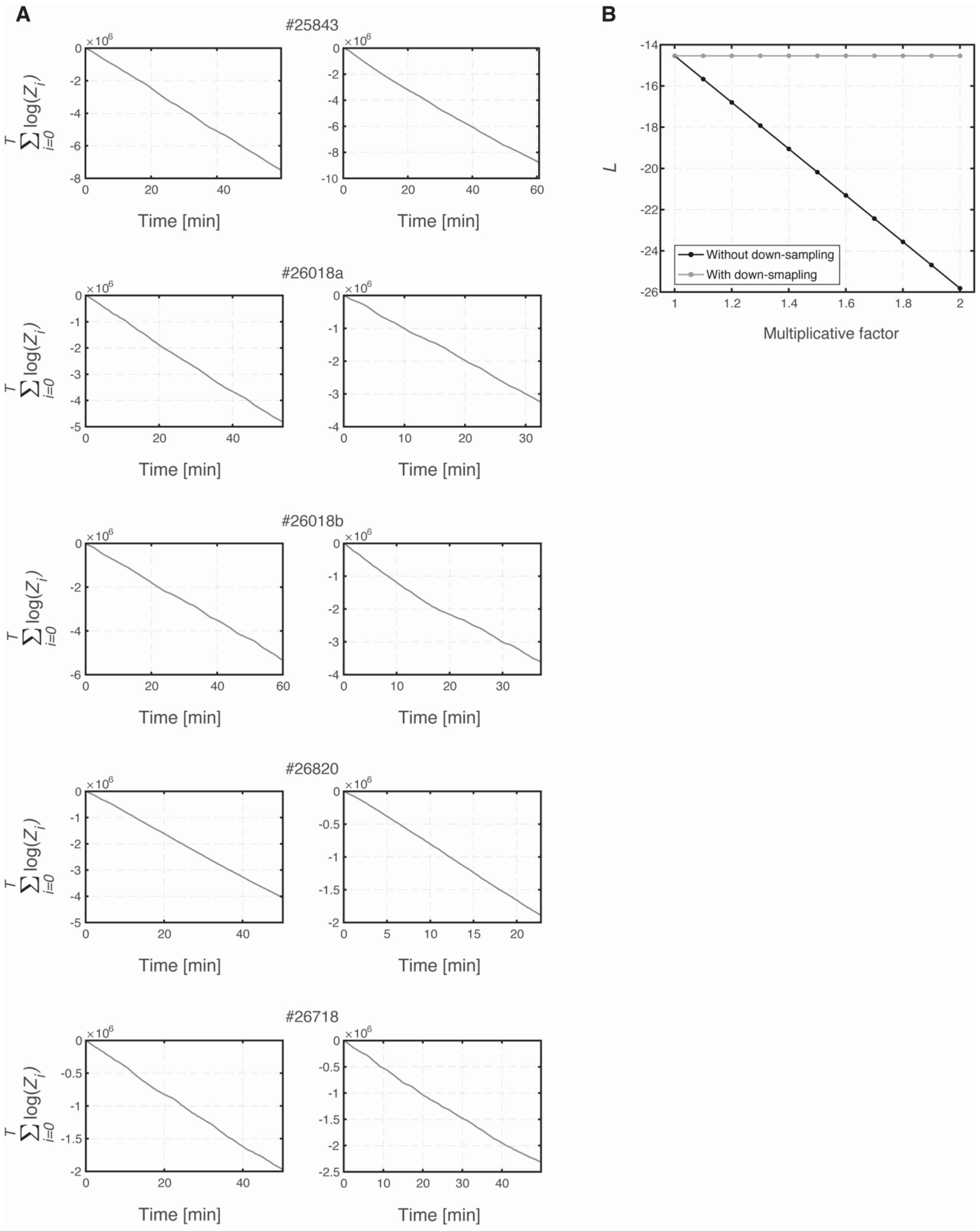
A
#25843
#26018a
#26018b
#26820
#26718
$\sum_{i=0}^{T} \log(Z_i)$
×10^6
Time [min]
B
L
Without down-sampling
With down-smapling
Multiplicative factor

Figure S8. Log likelihood versus time and the likelihood dependence on the mean firing rate. Related to STAR Methods and Methods S1.

A, Cumulative sum over time of $\log[p(\boldsymbol{S}_t)]$ in dark (left column) and in light (right column) trials from all recording sessions. Linear dependence (R^2>0.99 for both light and dark and across all sessions, fit not shown) is evident, indicating a steady accumulation of the log likelihood per time bin. **B,** The likelihood L was evaluated when using rate maps and light trajectory from recording session #26018b but with simulated Poisson spikes. Rate maps were first scaled by a multiplicative factor (x axis) thus leading to a constant decrease in the likelihood as the firing rate increases (black trace, as expected by Eq. S12). However, when the spike trains that were generated using the scaled rate maps were randomly down-sampled to match their original mean firing rate, the original likelihood value was precisely restored (gray trace).
